# CLEAR report 1: a scoping review and meta-analysis for definitions, imaging metrics, and functional correlates of photoreceptor integrity in AMD

**DOI:** 10.3389/fmed.2026.1887548

**Published:** 2026-07-06

**Authors:** Kimberly L. Spooner, Anjali Gaston, Anushka Irodi, Alicia Lim, Omer Trivizki, Livia Faes, Sobha Sivaprasad, Dun Jack Fu

**Affiliations:** 1 Save Sight Institute, The University of Sydney, Sydney, NSW, Australia; 2 The Graduate School of Health, The University of Technology Sydney, Sydney, NSW, Australia; 3 NIHR Biomedical Research Centre at Moorfields Eye Hospital NHS Foundation Trust, UCL Institute of Ophthalmology, London, United Kingdom; 4 Imperial College London, London, United Kingdom; 5 University of Cambridge, Cambridge, United Kingdom; 6 Department of Ophthalmology, Bascom Palmer Eye Institute, University of Miami Miller School of Medicine, Miami, FL, United States; 7 Department of Ophthalmology, Tel Aviv Medical Center, University of Tel Aviv, Tel Aviv, Israel; 8 University of Zurich, Institute of Epidemiology, Biostatistics and Prevention, Zurich, Switzerland; 9 Medignition Inc. Research Consultants Zurich, Zurich, Switzerland; 10 Cantonal Hospital Winterthur, Winterthur, Switzerland

**Keywords:** age related macular degeneration, biomarkers, ellipsoid zone (EZ), external limiting membrane, outer nuclear layer, photoreceptor, reliability, scoping review

## Abstract

**Purpose:**

Advanced age-related macular degeneration (AMD) is a leading cause of irreversible vision loss worldwide, with photoreceptor degeneration representing the final common pathway of functional impairment. Optical coherence tomography (OCT) enables noninvasive, layer-resolved quantification of photoreceptor integrity using biomarkers of the ellipsoid zone (EZ), the outer nuclear layer (ONL), and the external limiting membrane (ELM). However, substantial heterogeneity in definitions, measurement protocols, and reporting practices limits cross-study comparability and the adoption of clinical trials. This scoping review with nested meta-analyses maps how axial photoreceptor biomarkers are operationalized, segmented, validated, and related to functional and multimodal endpoints in AMD.

**Methods:**

MEDLINE, Embase, and Scopus were searched from January 2015 to December 2025 following PRISMA-ScR guidelines. Eligible studies reported OCT-based photoreceptor biomarkers in AMD populations. Data extraction captured boundary definitions, imaging platforms, segmentation approaches, analytic domains, ROI strategies, phenotypic context, reliability statistics, structure–function correlations, and OCT–FAF agreement.

**Results:**

Ninety-four studies met inclusion criteria, spanning spectral-domain and swept-source OCT platforms and diverse AMD phenotypes. Marked variability was observed in boundary definitions (e.g., EZ–RPE vs. EZ–Bruch’s membrane), measurement strategies (thickness, area, volume, reflectivity), and spatial sampling (central 1 mm, ETDRS subfields, lesion-centric masks). When segmentation boundaries and analytic conventions were explicitly defined and consistently applied, reliability for EZ and ONL metrics was high (ICC > 0.90), with automated and deep-learning methods performing comparably to expert manual grading. Structure–function analyses demonstrated moderate-to-strong correlations between photoreceptor loss and microperimetry sensitivity (*r* = 0.50–0.80) and best-corrected visual acuity (*r* = 0.40–0.70), particularly in advanced disease stages. OCT–FAF agreement for geographic atrophy detection was similarly high (κ > 0.80), although early atrophic features showed greater definitional variability. Phenotypic stratification and topographic reporting were inconsistently documented.

**Conclusion:**

OCT-derived photoreceptor biomarkers show high analytical reliability and clinically meaningful structure–function associations when segmentation boundaries, analytic domains, and reporting conventions are clearly specified. However, the review identifies substantial construct heterogeneity that complicates cross-study comparability and endpoint interpretation. Adoption of consensus boundary definitions, minimum reporting standards, and harmonized validation frameworks is necessary to ensure reproducibility, facilitate regulatory evaluation, and enable integration of photoreceptor metrics into AMD prevention and early-intervention trials. These findings provide the conceptual foundation for the CLEAR initiative (Consensus Layer Evaluation for AI Algorithm Reporting).

## 1 Introduction

Advanced age-related macular degeneration (AMD) [namely geographic atrophy (GA) and neovascular AMD (nAMD)] is associated with major functional disability, reduced quality of life, loss of independence, increased risk of depression and falls (1), and critically is a leading cause of irreversible vision loss in older adults worldwide, and its prevalence is expected to increase substantially (2, 3). Divergent regulatory decisions on complement inhibitors for GA across regulatory bodies have introduced confusion to the therapeutic landscape (4, 5), intensifying the need for sensitive, reproducible structural endpoints that capture disease activity earlier than conventional visual acuity or GA area alone.

Visual loss in AMD is most directly attributable to photoreceptor degeneration and dysfunction, which, although often preceded by RPE and choriocapillaris compromise, represents the proximate structural correlate of irreversible functional impairment (6, 7). Histologic and *in vivo* OCT studies show that thinning and disruption of the photoreceptor biomarkers ellipsoid zone (EZ) and outer nuclear layer (ONL) can precede clinically evident atrophy and progress alongside established lesions, suggesting both early-marker and disease-tracking potential (8, 9). These structural changes are closely linked to functional impairment, particularly under low-luminance or scotopic conditions, which are not captured by standard high-contrast acuity (10, 11). Microperimetry and psychophysical studies consistently demonstrate that local EZ or ONL loss corresponds to reduced retinal sensitivity and scotomata (12, 13).

Optical coherence tomography (OCT) has enabled non-invasive, layer-resolved imaging of these outer retinal changes. Advances from time-domain to spectral-domain and swept-source OCT have improved axial resolution, depth penetration, and volumetric acquisition, supporting quantitative assessment of EZ, ONL, external limiting membrane (ELM), and related metrics of photoreceptor integrity (14–16). Quantitative measures now include thickness, area, volume loss, reflectivity-derived indices, and can be analyzed in B-scan, *en face*, or fully volumetric domains (17, 18). However, several of these measures, particularly reflectivity- and continuity-based EZ metrics, are sensitive to acquisition quality, segmentation conventions, and analytic choices, introducing potential ambiguity between biological change and measurement artifact. In parallel, automated and deep learning-based segmentation has made large-scale, reproducible quantification feasible in multicenter trials (19–21). Despite these advances, most OCT-derived photoreceptor biomarkers rely on axial thickness or reflectivity-based surrogates whose interpretation depends on how anatomical boundaries are defined and operationalized. The extent to which these measurements reflect stable biological constructs rather than boundary-dependent analytical conventions has not been systematically examined. Critically, AI performance and biomarker validity cannot be interpreted independently of the anatomical construct definition used as the reference standard.

The Classification of Atrophy Meeting (CAM) consensus established OCT-based definitions for incomplete and complete RPE and outer retinal atrophy (iRORA and cRORA), providing a standardized framework for GA assessment that complements fundus autofluorescence (FAF) (22–24). However, photoreceptor-specific biomarkers, which may precede complete atrophy and offer earlier intervention windows, remain heterogeneously defined and inconsistently reported across studies. This heterogeneity limits cross-study synthesis, regulatory acceptance, and clinical trial implementation. In response to these challenges, the Consensus Layer Evaluation for AI Algorithm Reporting (CLEAR) initiative was established to support harmonized evaluation of OCT-derived photoreceptor biomarkers and AI-enabled imaging endpoints in AMD. The present manuscript represents CLEAR Report 1 and focuses on the definitional, anatomical, and methodological foundations underpinning photoreceptor biomarker interpretation, reproducibility, and regulatory readiness.

Despite growing interest in photoreceptor biomarkers, the literature remains characterized by diverse operational definitions, measurement protocols, segmentation approaches, degrees of operability and robustness, and reporting conventions. Traditional systematic reviews with strict inclusion criteria may exclude valuable methodological insights, while purely narrative reviews lack quantitative synthesis of evidence on reliability and validity. A scoping review methodology, designed to map heterogeneous evidence and identify knowledge gaps, is ideally suited to this landscape (25, 26). By incorporating structured quantitative synthesis within the scoping framework, we summarize reliability metrics and structure–function associations while preserving the breadth necessary to capture definitional variability and methodological diversity.

This scoping review was designed to address five interrelated domains: (1) how axial photoreceptor OCT biomarkers are anatomically defined and operationalized; (2) segmentation methodology and reliability; (3) phenotypic context and reporting completeness; (4) structure–function relationships; and (5) concordance between OCT- and FAF-based atrophy assessment.

By integrating these domains, the review aims to delineate the current evidentiary foundation for photoreceptor biomarkers as candidate trial endpoints, identify areas of methodological convergence and persistent heterogeneity, and inform ongoing efforts toward consensus standardization, AI validation, and regulatory readiness within the CLEAR framework.

## 2 Methods

### 2.1 Methodological framework

This scoping review was designed and conducted according to the Preferred Reporting Items for Systematic Reviews and Meta-Analyses extension for Scoping Reviews (PRISMA-ScR) (27). For domains in which pooling was performed, we evaluated the consistency of definitions and measurement conditions but did not apply formal quality scores, as these are not validated for the heterogeneous methodological landscape addressed by this review. We selected a scoping approach to comprehensively map the heterogeneous landscape of photoreceptor biomarker definitions, measurement protocols, and supporting evidence across the full spectrum of age-related macular degeneration. This broad mapping strategy was complemented by a structured quantitative synthesis of reliability and agreement metrics in domains with sufficient methodological comparability.

### 2.2 Eligibility criteria and outcomes

We included original clinical studies that reported quantitative axial measures of photoreceptor integrity or outer retinal structure, assessed using OCT or OCT-derived metrics, in eyes with AMD. Eligible study designs comprised randomized controlled trials, prospective and retrospective cohort studies, and case–control studies. Case series with fewer than 10 eyes, narrative reviews, editorials, conference abstracts without full text, and non-human studies were excluded.

Studies were required to report at least one predefined structural photoreceptor-related outcome corresponding anatomically to the photoreceptor unit, including metrics reflecting the photoreceptor axon (Henle fiber layer), cell body [outer nuclear layer (ONL)], inner segments [e.g., ellipsoid zone (EZ)], outer segments [e.g., interdigitation zone (IZ) or EZ–RPE thickness], or composite outer retinal biomarkers incorporating multiple photoreceptor substructures. For eligibility, imaging biomarkers were categorized based on the anatomical photoreceptor component they purported to measure (axon, cell body, inner segment, outer segment, or composite). Studies reporting integrity, thickness, reflectivity, or attenuation of these structures were included. Functional outcomes were extracted when available; however, they were not required for inclusion. Studies were not excluded based on OCT platform, scan density, or segmentation approach, reflecting the aim of mapping real-world methodological variability rather than enforcing artificial uniformity. Studies reporting only global retinal thickness measures without photoreceptor-specific assessment were excluded.

Where possible, we sought to extract phenotypic disease characteristics with established biological relevance, including drusen subtype, presence of SDD, and MNV subtype, as these features have been shown to reflect distinct pathophysiological pathways and progression patterns in retinal disease. However, no study was excluded for incomplete phenotypic reporting.

### 2.3 Literature search strategy

A comprehensive literature search was performed in MEDLINE (via PubMed), Embase, and Scopus. Searches were restricted to studies published between 1 January 2015 and 31 December 2025 and to English-language articles. The 2015 threshold was selected a priori to capture contemporary OCT imaging technologies, segmentation methodologies, and photoreceptor biomarker definitions relevant to current clinical and research practice. The search strategy combined controlled vocabulary and free-text terms relating to photoreceptor integrity, outer retinal structure, OCT imaging, and the disease of interest.

The full electronic search strategy for each database is provided in Supplementary methods. Reference lists of included studies and relevant reviews were manually screened to identify additional eligible articles. The search strategy was designed to prioritize sensitivity over specificity, with subsequent filtering at the screening stage.

### 2.4 Study selection

All records identified through the database searches were imported into a reference management software, and duplicates were removed. Two reviewers independently screened titles and abstracts for eligibility. Full-text articles were retrieved for all potentially relevant records and assessed independently by the same reviewers against the predefined inclusion and exclusion criteria. Discrepancies at any stage were resolved through discussion, with adjudication by a third reviewer when necessary.

**FIGURE 1:**
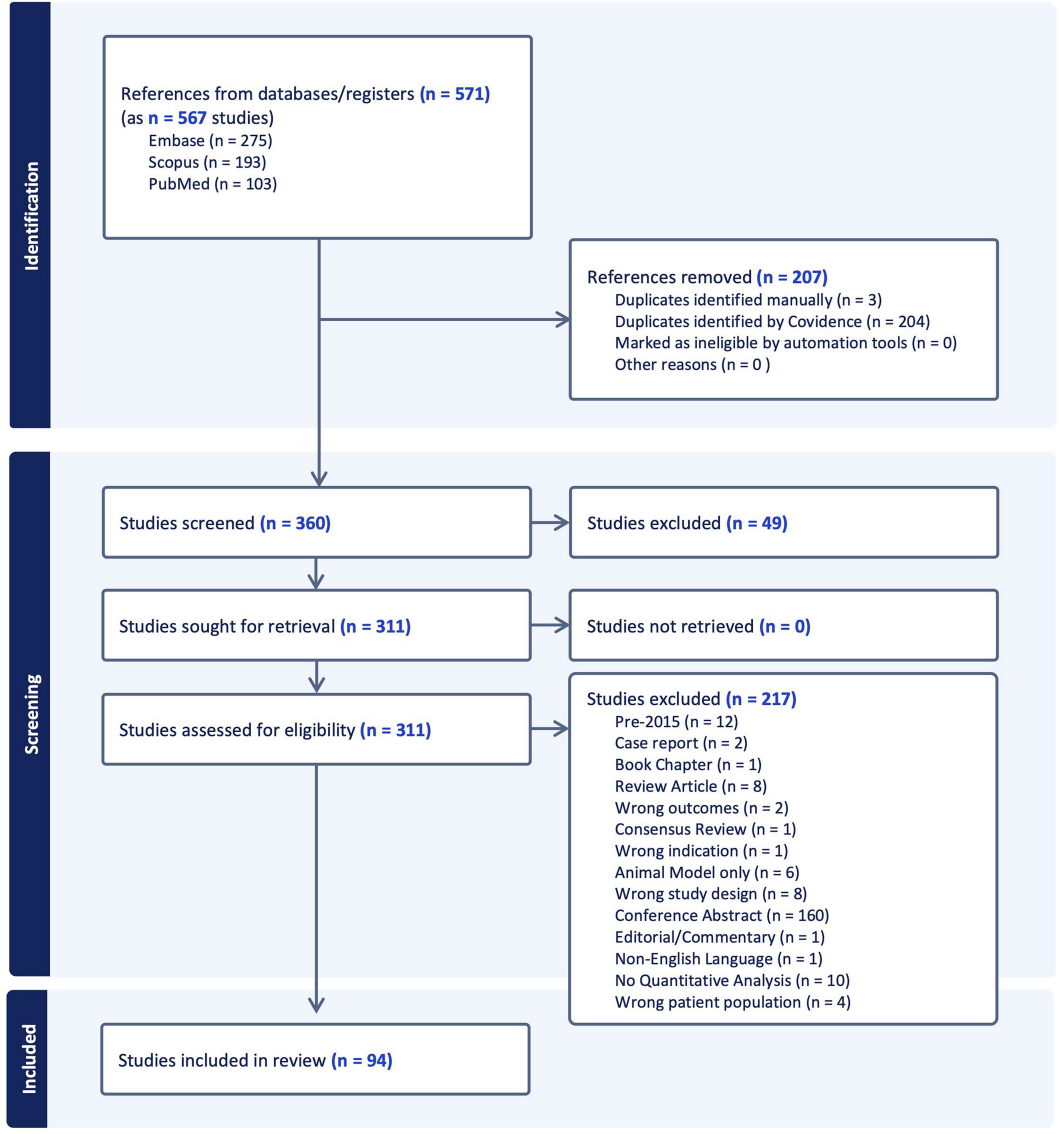
Definitions, segmentation methodology, and inter-grader reliability of photoreceptor-related SD-OCT biomarkers across retinal diseases: a scoping review.

The study selection process is summarized using a PRISMA flow diagram, detailing the number of records identified, screened, excluded, and included (Figure 1).

### 2.5 Data extraction and phenotype classification

Data extraction was performed independently by two reviewers using a standardized, pilot-tested extraction form. Extracted data included study design, sample size, demographic characteristics, disease indication and stage, imaging modality and acquisition parameters, photoreceptor-related structural outcomes, and statistical estimates relevant to reliability and agreement assessment. Detailed technical measurement parameters underpinning each photoreceptor biomarker, including segmentation boundaries, spatial scale, and region of interest, thresholding or normalization logic, analytic domain, and reported acquisition or grading constraints affecting operability, were systematically extracted and are summarized in Table 1.

**TABLE 1:** Technical measurement parameters of OCT-derived photoreceptor biomarkers in AMD.

Study	Photoreceptor term used in study	Inner boundary	Outer boundary	Boundary specification adequacy*	Measurement domain	Spatial Scale/ROI	Quantification Type	Thresholding/ Normalization
Abraham et al. (31) (Ophth Retina)	EZ–RPE thickness	EZ (border not specified; Cole RC)/EZ posterior surface (Duke RC implied)	RPE (surface not specified; Cole RC)/OS tip–inner RPE boundary (Duke RC)	Full	Volumetric + en face	ETDRS subfields	Thickness; area	EZ–RPE = 0 μm; ≤ 20 μm
Abraham et al. (31) (Ophth Retina)	ONL–RPE thickness	ONL posterior boundary (Cole RC)/OPL–ONL boundary (Duke RC)	RPE (unspecified)/OS tip–inner RPE boundary	Full	Volumetric + en face	ETDRS subfields	Thickness; area	EZ–RPE = 0 μm; ≤ 20 μm
Abraham et al. (31) (Ophth Retina)	Photoreceptor layer (ELM–OS/RPE)	ELM	OS tip/inner RPE boundary	Full	Volumetric + en face	ETDRS subfields	Thickness; area	EZ–RPE = 0 μm; ≤ 20 μm
Abraham et al. (31) (Ophth Retina)	Outer retina (OPL/ONL–OS/RPE)	OPL/ONL boundary	OS tip/inner RPE boundary	Full	Volumetric + en face	ETDRS subfields	Thickness; area	EZ–RPE = 0 μm; ≤ 20 μm
Bell et al. (32)	EZ integrity	EZ centreline (segmented through centre of band)	Not a thickness construct	Full	Volumetric	Central 2 mm	Binary/thickness	None
Bell et al. (32)	EZ–RPE thickness	EZ centreline	RPE segmentation line	Full	Volumetric	Central 2 mm	Binary/thickness	None
Bell et al. (32)	ILM–RPE thickness	ILM	RPE segmentation line	Full	Volumetric	Central 2 mm	Binary/thickness	None
Bell et al. (32)	Sub-RPE compartment (RPE–BM)	RPE segmentation line	BM	Full	Volumetric	Central 2 mm	Binary/thickness	None
Birner et al. (33)	EZ thickness	Inner boundary of EZ band	Outer boundary of IZ	Full	B-scan	∼70 μm radius at MP loci	Thickness	None
Birner et al. (33)	ONL thickness	Outer border of OPL	ELM	Full	B-scan	∼70 μm radius at MP loci	Thickness	None
Birner et al. (33)	Drusen volume	BM	Outer border of RPE	Full	B-scan	∼70 μm radius at MP loci	Volume	None
Birner et al. (33)	HRF volume	Not defined by fixed interfaces (manual annotation)	Not defined by fixed interfaces	Full	B-scan	∼70 μm radius at MP loci	Volume	None
Birner et al. (33)	SDD volume	RPE surface	BM band	Full	B-scan	∼70 μm radius at MP loci	Volume	None
Birner et al. (34)	EZ thickness	Inner boundary of EZ	Outer boundary of IZ	Full	B-scan + volumetric	MP-aligned loci	Thickness; area	EZL vs. EZL + RPEL
Birner et al. (34)	EZ loss	Inner boundary of EZ	Outer boundary of IZ	Full	B-scan + volumetric	MP-aligned loci	Area	EZL vs. EZL + RPEL
Birner et al. (34)	RPE loss	RPE band ( ≥ 250 μm attenuation; CAM)	Not thickness-defined (DL loss map)	Full	B-scan + volumetric	MP-aligned loci	Area	EZL vs. EZL + RPEL
Birner et al. (34)	Drusen volume	BM	Outer border of RPE	Full	B-scan + volumetric	MP-aligned loci	Volume	EZL vs. EZL + RPEL
Bogunovic et al. (35)	ONL thickness	Algorithm-defined ONL inner boundary (Iowa)	Algorithm-defined ONL outer boundary	Partial	Volumetric	Macular grid	Thickness	None
Bogunović et al. (35)	ORB thickness	Lower ONL boundary	RPE surface	Partial	Volumetric	Macular grid	Thickness	None
Bogunović et al. (35)	Drusen thickness	RPE surface	BM surface	Partial	Volumetric	Macular grid	Thickness	None
Borrelli et al. (36)	EZ volume	ELM	IZ	Full	Volumetric	Macular ROIs	Volume	None
Borrelli et al. (36)	OS volume	EZ	IZ	Full	Volumetric	Macular ROIs	Volume	None
Borrelli et al. (37)	EZ normalized reflectivity	45 μm above RPE reference (geometric slab)	21 μm slab thickness (45–66 μm above RPE)	Partial	En face	Macular slab	Normalised intensity	EZ ÷ reference
Brandl et al. (17)	ONL thickness	OPL outer border	ELM	Full	Volumetric	ETDRS 1/3/6 mm	Thickness	None
Brandl et al. (17)	ORL thickness	ELM	BrM	Full	Volumetric	ETDRS 1/3/6 mm	Thickness	None
Brandl et al. (17)	RPE/BrM thickness	RPE	BrM	Full	Volumetric	ETDRS 1/3/6 mm	Thickness	None
Brandl et al. (17)	PR-IS/OS thickness	ELM	RPE	Full	Volumetric	ETDRS 1/3/6 mm	Thickness	None
Carvajal et al. (38)	iRORA	Not interface-defined (CAM criteria: RPE attenuation + hypertransmission + overlying PR degeneration incl. EZ/ELM/ONL)	Not interface-defined	Insufficient	B-scan	ETDRS grid	Binary; area	CAM GLD ≥ 250 μm
Carvajal et al. (38)	cRORA	Not interface-defined (CAM morphologic definition)	Not interface-defined	Insufficient	B-scan	ETDRS grid	Binary; area	CAM GLD ≥ 250 μm
Cedro et al. (39)	cRORA area	Not interface-defined (manual IR delineation + OCT confirmation; CAM-based)	Not interface-defined	Insufficient	B-scan	Lesion-centric	Area Ordinal Ordinal	Square-root transform
Cedro et al. (39)	ELM disruption score	Qualitative grading	Qualitative grading	Insufficient	B-scan	Lesion-centric	Area Ordinal Ordinal	Square-root transform
Cedro et al. (39)	IS/OS disruption score	Qualitative grading	Qualitative grading	Insufficient	B-scan	Lesion-centric	Area Ordinal Ordinal	Square-root transform
Cheung et al. (40)	EZ sinuosity	EZ centreline (single line through hyperreflective band)	BM centreline (reference denominator)	Partial	B-scan	Central macula	Ratio R	EZ > 1.01
Cheung et al. (40)	RPE sinuosity	RPE centreline	BM centreline	Partial	B-scan	Central macula	Ratio R	RPE > 1.03
Coulibaly et al. (46)	EZ loss (EZL)	EZ segmentation line (AI-derived; inner/outer interface not explicitly specified)	Not thickness-defined (loss map)	Partial	Volumetric	Lesion-centric	Area (√mm) Area Ar	Square-root growth rate
Coulibaly et al. (46)	RPE loss (RPEL)	RPE segmentation line	Not thickness-defined	Partial	Volumetric	Lesion-centric	Area (√mm) Area Ar	Square-root growth rate
Coulibaly et al. (46)	ELM loss (ELML)	ELM segmentation line	Not thickness-defined	Partial	Volumetric	Lesion-centric	Area (√mm) Area Ar	Square-root growth rate
Ehlers et al. (47)	OCT-GA (RPE loss)	RPE segmentation line	Bruch’s membrane (GA defined as RPE–BM = 0 μm)	Partial	Volumetric	Whole lesion	Area	FAF cross-confirmation
Etheridge et al. (49)	ONL thickness	OPL/ONL boundary (device-defined)	ELM	Partial	Volumetric	ETDRS rings	Thickness	Age-adjusted models
Etheridge et al. (49)	Outer retinal thickness	ELM	BM	Partial	Volumetric	ETDRS rings	Thickness	Age-adjusted models
Etheridge et al. (49)	PR layer	Device-defined PR compartment	Device-defined	Partial	Volumetric	ETDRS rings	Thickness	Age-adjusted models
Farinha et al. (50)	ONL thickness	OPL → ELM (device autosegmentation)	ELM	Partial	Volumetric	ETDRS 1/3/6 mm	Thickness	Bonferroni correction
Farinha et al. (50)	PRL (photoreceptor layer)	ELM	RPE/BrM complex	Partial	Volumetric	ETDRS 1/3/6 mm	Thickness	Bonferroni correction
Farinha et al. (50)	ORL	ELM	BrM	Partial	Volumetric	ETDRS 1/3/6 mm	Thickness	Bonferroni correction
Fasih-Ahmad et al. (51)	IZ thickness	Inner IZ boundary (hyperreflective pixel subjacent to EZ)	Outer IZ boundary (hyperreflective pixel above RPE)	Full	En face + volumetric	ETDRS outer ring	Thickness	None
Fasih-Ahmad et al. (51)	EZ area	EZ inner boundary	IZ outer boundary	Full	En face + volumetric	ETDRS outer ring	Area	None
Flynn et al. (53)	EZ integrity category	Qualitative EZ band continuity	Qualitative absence/presence	Insufficient	B-scan	Functional loci	Ordinal	Intact/attenuated/absent
Futterknecht et al. (56)	EZ/IZ loss	EZ/IZ disruption zone (no explicit anterior/posterior interface specification)	Hypertransmission region	Insufficient	Volumetric + targeted MP	Lesion-targeted grids	Sensitivity; lesion area	Distance- and size-adjusted error models
Gallagher et al. (57)	ONL thickness	OPL	ELM	Full	Volumetric	Central macula	Thickness	None
Gallagher et al. (57)	OSL (ELM–EZ–IZ band)	ELM	Outer border of EZ–IZ complex	Full	Volumetric	Central macula	Thickness	None
Gallagher et al. (57)	RPE–BrM thickness	RPE	BrM	Full	Volumetric	Central macula	Thickness	None
Ghoshal et al. (58)	ONL thickness	OPL	ELM	Partial	Volumetric	ETDRS central + inner ring	Thickness	None
Ghoshal et al. (58)	ORT (outer retina thickness)	ELM	BM	Partial	Volumetric	ETDRS central + inner ring	Thickness	None
Ghoshal et al. (58)	RPE–BM thickness	RPE	BM	Partial	Volumetric	ETDRS central + inner ring	Thickness	None
Goerdt et al. (59)	EZ band visibility	Qualitative band identification	Qualitative	Insufficient	B-scan	Eccentricity-specific loci	Visibility (%)	None
Goerdt et al. (59)	OSIZ-1/2 visibility	Qualitative band identification	Qualitative	Insufficient	B-scan	Eccentricity-specific loci	Visibility (%)	None
Griffin et al. (60)	EZ reflectivity	EZ reflectivity peak (no explicit inner/outer band definition)	Not interface-defined	Partial	B-scan	Pixel-level ROIs	Normalized intensity	EZ ÷ IPL
Hanna et al. (61)	Photoreceptor (PR) layer	Inner/outer segment junction (device-defined)	RPE segmentation line	Partial	Volumetric	ETDRS central	Thickness	Paired change vs. baseline
Hanna et al. (61)	ONL thickness	Device-defined ONL boundary	Device-defined ONL boundary	Partial	Volumetric	ETDRS central	Thickness	Paired change vs. baseline
Hanna et al. (61)	Sub-RPE thickness	RPE	Bruch’s membrane (BM)	Partial	Volumetric	ETDRS central	Thickness	Paired change vs. baseline
Heckenlaible et al. (62)	OS thickness	EZ	IZ	Partial	Volumetric	ETDRS subfields	Thickness	Coefficient of variation
Heckenlaible et al. (62)	EZ thickness	Algorithm-defined ELM–EZ interface	Algorithm-defined EZ–IZ interface	Partial	Volumetric	ETDRS subfields	Thickness	Mean reflectivity
Heckenlaible et al. (62)	EZ reflectivity	EZ band (reflectivity-based)	Not thickness-defined	Partial	Volumetric	ETDRS subfields	Reflectivity	Internal normalisation
Heiferman et al. (63)	SDD presence	Slab above RPE (manually positioned)	Slab below ELM	Insufficient	En face + volumetric	Lesion-centric	Counts; distance metrics	None
Ho et al. (64)	EZ absence	Qualitative EZ band presence	Qualitative absence	Insufficient	B-scan + perimetry	Pointwise loci	Binary + sensitivity	None
Ho et al. (64)	RPE disruption	Qualitative RPE morphology	Qualitative	Insufficient	B-scan + perimetry	Pointwise loci	Binary + sensitivity	None
Hong et al. (65)	ONL thickness	OPL	ELM	Full	Volumetric	Macular ROIs	Thickness	None
Hong et al. (65)	EZ degeneration (GA component)	Qualitative EZ loss	Qualitative	Full	Volumetric	Macular ROIs	Binary	None
Itoh et al. (66)	EZ–RPE thickness	EZ segmentation line (algorithm-identified)	RPE “floor” segmentation line	Partial	Volumetric + en face	Macula-wide	Volume; area	EZ–RPE ≤ 20 μm
Itoh et al. (66)	EZ loss area	EZ absent relative to RPE	Not thickness-defined	Partial	Volumetric + en face	Macula-wide	Area	EZ–RPE ≤ 20 μm
Kalra et al. (67)	EZ + RPE loss concurrence	EZ segmentation line	RPE segmentation line	Partial	B-scan + en face	Whole lesion	Area	CAM-aligned HT thresholds
Kalra et al. (67)	Hypertransmission defect	RPE attenuation region	BM	Partial	B-scan + en face	Whole lesion	Area	CAM-aligned HT thresholds
Kalra et al. (68)	EZ At-Risk area	EZ segmentation line	RPE segmentation line	Partial	Volumetric	Macula-wide	Area (%)	EZ–RPE ≤ 10 μm
Kar et al. (69)	EZ–RPE texture compartment	EZ segmentation line	RPE segmentation line	Partial	Volumetric	Central macula	Radiomics	Z-score standardization
Kar et al. (69)	Sub-RPE fractal features	RPE	BM	Partial	Volumetric	Central macula	Texture features	Z-score standardisation
Liermann et al. (70)	rEZR (EZ/ELM ratio)	EZ reflectivity peak	ELM reflectivity peak	Partial	Raw B-scan	Central ETDRS	Ratio	Internal reflectivity normalization
Mahmoudi et al. (71)	EZ disruption width	EZ band interruption	EZ band reappearance	Insufficient	B-scan	Lesion margins	Length Length	None
Mahmoudi et al. (71)	ELM disruption width	ELM band interruption	ELM band reappearance	Insufficient	B-scan	Lesion margins	Length Length	None
Mahmoudi et al. (71)	Hypertransmission width	Hypertransmission start	Hypertransmission end	Insufficient	B-scan	Lesion margins	Length Length	None
Mai et al. (72)	EZ loss area	EZ segmentation line	IZ outer boundary	Partial	Volumetric	Whole lesion	Area	DL probability mask
Mai et al. (72)	RPE loss area	RPE segmentation line	Not thickness-defined	Partial	Volumetric	Whole lesion	Area	DL probability mask
Mai et al. (73)	EZ thickness	Top of EZ (algorithm-defined)	Outer boundary of IZ	Partial	Volumetric	Lesion-centric	Thickness Ratio	EZ ≤ 4 μm
Mai et al. (73)	EZ/RPE loss ratio	EZ loss map	RPE loss map	Partial	Volumetric	Lesion-centric	Thickness Ratio	Quartile stratification
Morelle et al. (74)	EZ height map	EZ predicted height surface (CNN)	RPE predicted surface	Partial	Volumetric	Macula-wide	Height Volume	None
Morelle et al. (74)	Drusen volume	RPE predicted surface	BM predicted surface	Partial	Volumetric	Macula-wide	Height Volume	None
Müller et al. (75)	EZ loss	Qualitative EZ absence	Qualitative	Insufficient	B-scan	ETDRS grid	Binary	None
Müller et al. (75)	RPE loss	Qualitative RPE absence	Qualitative	Insufficient	B-scan	ETDRS grid	Binary	None
Pfau et al., (80) (IOVS)	Residual HFL + ONL within GA	OPL/ONL boundary (structural identification at stimulus loci)	External limiting membrane (ELM)	Insufficient	Volumetric	Intra-lesional loci	Thickness (presence/absence persistence)	None
Pfau et al. (80) (IOVS)	ELM descent	ELM band position	Bruch’s membrane (BM) reference	Insufficient	Volumetric	Intra-lesional loci	Positional classification	None
Pfau et al. (80) (AJO)	ONL thickness (incl. HFL)	OPL/ONL boundary	ELM	Full	Volumetric	GA contour lines	Thickness	Eccentricity-matched norms
Pfau et al. (80) (AJO)	Inner segment (IS) thickness	ELM	EZ	Full	Volumetric	GA contour lines	Thickness	Eccentricity-matched norms
Pfau et al., (80) (AJO)	Outer segment (OS) thickness	EZ	IZ	Full	Volumetric	GA contour lines	Thickness	Eccentricity-matched norms
Pfau et al. (80) (AJO)	RPEDC thickness	Inner RPE boundary	Bruch’s membrane (BM)	Full	Volumetric	GA contour lines	Thickness	Eccentricity-matched norms
Pfau et al. (78) (JAMA Ophthalmol)	ONL thickness	Algorithm-defined ONL compartment	Algorithm-defined ONL compartment	Partial	Volumetric	GA contour lines	Thickness	Eccentricity-normalised
Pfau et al. (78) (JAMA Ophthalmol)	IS thickness	Algorithm-defined IS compartment	Algorithm-defined IS compartment	Partial	Volumetric	GA contour lines	Thickness	Eccentricity-normalised
Pfau et al. (78) (JAMA Ophthalmol)	OS thickness	Algorithm-defined OS compartment	Algorithm-defined OS compartment	Partial	Volumetric	GA contour lines	Thickness	Eccentricity-normalised
Pfau et al. (78) (JAMA Ophthalmol)	EZ-loss distance	EZ loss margin	GA edge	Partial	Volumetric	GA contour lines	Distance	Eccentricity-normalised
Pfau et al. (76) (TVST)	ONL variability (SD)	Algorithm-defined ONL boundary	Algorithm-defined ONL boundary	Partial	Volumetric	Macula-wide	Variability (SD)	None
Pfau et al. (76) (TVST)	IS thickness variability	Algorithm-defined IS	Algorithm-defined IS	Partial	Volumetric	Macula-wide	Variability (SD)	None
Pfau et al. (76) (TVST)	OS thickness variability	Algorithm-defined OS	Algorithm-defined OS	Partial	Volumetric	Macula-wide	Variability (SD)	None
Pfau et al. (77) (Sci Rep)	ONL thickness (outside GA)	Algorithm-defined ONL boundary	Algorithm-defined ONL boundary	Partial	Volumetric	0.43°, 2.58°, 5.16° contours	z-score thickness	Baseline-adjusted
Pfau et al. (77) (Sci Rep)	IS thickness	Algorithm-defined IS	Algorithm-defined IS	Partial	Volumetric	0.43°, 2.58°, 5.16° contours	z-score thickness	Baseline-adjusted
Pfau et al. (77) (Sci Rep)	OS thickness	Algorithm-defined OS	Algorithm-defined OS	Partial	Volumetric	0.43°, 2.58°, 5.16° contours	z-score thickness	Baseline-adjusted
Prenner et al. (81)	PR thickness	Outer boundary of IZ	Top of EZ	Full	Volumetric	ETDRS 1/3 mm	Thickness	Device comparison
Prenner et al. (81)	RPE thickness	Outer boundary of RPE	Outer boundary of IZ	Full	Volumetric	ETDRS 1/3 mm	Thickness	Device comparison
Prenner et al. (81)	ELM loss	ELM band absence	Not thickness-defined	Full	Volumetric	ETDRS 1/3 mm	Binary	Device comparison
Qu et al. (82) (RETINA)	Photoreceptor layer thickness	EZ	Inner surface of RPE	Full	En face + volumetric	Lesion margins	Thickness; defect area	None
Qu et al. (82) (RETINA)	RPE layer thickness	Inner surface of RPE	Outer RPE/BM complex	Full	En face + volumetric	Lesion margins	Thickness	None
Riedl et al. (83)	EZ integrity	Qualitative EZ band continuity	Qualitative	Insufficient	B-scan	ETDRS subfields	% intact	None
Riedl et al. (84) (IOVS)	PR thickness	Inner border of EZ	Inner border of RPE	Full	Volumetric	Drusen-centric ROIs	Thickness	None
Riedl et al. (84) (IOVS)	ONL thickness	CNN-derived ONL upper boundary	CNN-derived ONL lower boundary	Full	Volumetric	Drusen-centric ROIs	Thickness	None
Rogala et al. (85)	PR-layer thickness	Posterior border of RPE	Midpoint of ELM	Partial	Volumetric	Drusen-overlying loci	Thickness	None
Rogala et al. (85)	ONL thickness	Posterior OPL	Midpoint of ELM	Partial	Volumetric	Drusen-overlying loci	Thickness	None
Russakoff et al. (86)	PR complex thickness	Algorithm-defined PR complex	Algorithm-defined PR complex	Partial	Volumetric + DL	Whole volume	Prediction score	Network normalization
Russakoff et al. (86)	RPE–BM thickness	RPE	BM	Partial	Volumetric + DL	Whole volume	Thickness	Network normalization
Sadigh et al. (87)	ONL^+^ thickness	OPL	Outer limiting membrane (OLM)	Full	Volumetric	Drusen-overlying loci	Thickness	None
Sadigh et al. (87)	IS + OS thickness	OLM	OS tip/apical RPE interface	Full	Volumetric	Drusen-overlying loci	Thickness	None
Saeed et al. (88)	EZ disruption	Qualitative EZ band loss	Qualitative	Insufficient	B-scan	Functional loci	Binary	None
Saeed et al. (2025)	ONL thickness	Not explicitly interface-defined	Not explicitly interface-defined	Insufficient	B-scan	Functional loci	Thickness	None
Sarici et al. (89)	EZ–RPE thickness	EZ segmentation line	RPE segmentation line	Partial	Volumetric	Central macula	Thickness	AUROC-optimised cut-offs
Sarici et al. (89)	RPE–BM thickness	RPE	BM	Partial	Volumetric	Central macula	Thickness	AUROC-optimized cut-offs
Saßmannshausen et al. (91)	ONL thickness (z-score)	ONL boundary (device-defined)	ONL boundary (device-defined)	Partial	Volumetric	Functional loci	z-score	Age-matched normalization
Saßmannshausen et al. (90)	rEZR	EZ reflectivity peak	ELM reflectivity peak	Partial	Volumetric	ETDRS subfields	Ratio	None
Savastano et al. (93)	ONL thickness	Device-defined ONL	Device-defined ONL	Partial	Volumetric	Lesion-centric	Thickness	SRI ≥ 1.5 mm^2^
Savastano et al. (93)	EZ interruption	Qualitative band loss	Qualitative	Partial	Volumetric	Lesion-centric	Binary	SRI ≥ 1.5 mm^2^
Sayegh et al. (94)	GA area	Hypertransmission region	GA border on FAF	Partial	Volumetric + FAF	1-mm and 3-mm zones	Area	Square-root GA growth
Schaal et al. (95)	Outer retinal tubulation (ORT)	Tubular structure based on ELM/IS morphology	Tubular structure boundary	Insufficient	B-scan + histology	Lesion margins	Presence; morphology	None
Schmidt-Erfurth et al. (96)	EZ loss area	DL-derived EZ loss map (segmentation interface not anatomically specified as inner/outer border)	Not thickness-defined (area map)	Partial	Volumetric	Whole lesion	Area	None
Schmidt-Erfurth et al. (96)	RPE loss area	DL-derived RPE loss map (segmentation interface not anatomically specified)	Not thickness-defined (area map)	Partial	Volumetric	Whole lesion	Area	None
Schmitz-Valckenberg et al. (97)	Hypertransmission length	Hypertransmission start (caliper-defined)	Hypertransmission end	Partial	B-scan	Lesion margins	Length	None
Schmitz-Valckenberg et al. (97)	EZ disruption length	EZ band interruption start	EZ band interruption end	Partial	B-scan	Lesion margins	Length	None
Schmitz-Valckenberg et al. (97)	ELM disruption length	ELM band interruption start	ELM band interruption end	Partial	B-scan	Lesion margins	Length	None
Schweighofer et al. (98)	MP repeatability vs. SDD	Not interface-defined (SDD presence on OCT; functional repeatability endpoints)	Not interface-defined	Insufficient	Functional + OCT	Test–retest loci	CoR; ICC	Device-specific
Song et al. (99)	DL probability of EZ/ELM disruption + fluid features	DL-derived feature maps (EZ/ELM disruption probabilities; no explicit interface borders reported)	Not thickness-defined	Partial	Volumetric + DL	Macular volume	Prediction probability	Feature scaling
Steinberg et al. (100)	Partial outer retinal thickness	OPL	RPE	Full	Volumetric	Functional loci	Thickness	None
Sulzbacher et al. (101)	Fluid phenotype vs. sensitivity (SRF/IRF/PED)	Not interface-defined (fluid compartment classification)	Not interface-defined	Insufficient	B-scan + MP	Pointwise loci	Sensitivity change	None
Inanc Tekin et al. (102)	ONL thickness	OPL	ELM	Full	Volumetric	Central macula	Thickness	None
Inanc Tekin et al. (102)	RPE thickness	RPE	BM	Full	Volumetric	Central macula	Thickness	None
Tepelus et al. (103)	RPE + OS volume	RPE + OS slab inner boundary (device-defined)	RPE + OS slab outer boundary (device-defined)	Partial	Volumetric	Central 8°	Volume	None
Tepelus et al. (103)	ONL volume	ONL slab inner boundary (device-defined)	ONL slab outer boundary (device-defined)	Partial	Volumetric	Central 8°	Volume	None
Thiele et al. (104)	rEZR (automated vs. manual)	EZ reflectivity peak	ELM reflectivity peak	Partial	Volumetric	Macular ROIs	Ratio	None
Thiele et al. (105)	rEZR (longitudinal decline)	EZ reflectivity peak	ELM reflectivity peak	Partial	Volumetric	ETDRS subfields	Ratio	None
Trinh et al. (108)	ONL + HFL thickness deviation	Normative atlas deviation (layer-wise)	Normative atlas deviation (layer-wise)	Full	Volumetric	Macula-wide	Z-score deviation	Normative atlas
Trinh et al. (107)	ONL thickness	Layer boundary (segmented)	Layer boundary (segmented)	Full	Volumetric	ETDRS subfields	Thickness	None
Trinh et al., 2022	IS/OS thickness	Layer boundary (segmented)	Layer boundary (segmented)	Full	Volumetric	ETDRS subfields	Thickness	None
Trinh et al., 2022	RPE–BM thickness	RPE	BM	Full	Volumetric	ETDRS subfields	Thickness	None
van Romunde et al. (109)	ELM integrity; PR disruption	Qualitative ELM/EZ grading	Qualitative	Insufficient	B-scan	Central fovea	Binary	None
Vogl et al. (110)	ONL thinning	Iowa-based ONL boundary (algorithm-defined)	Iowa-based ONL boundary (algorithm-defined)	Partial	Volumetric	ETDRS-style rings + central 3 mm	Thickness	FDR correction
Vogl et al. (110)	ORB thinning	Iowa-based ORB compartment boundary	Iowa-based ORB compartment boundary	Partial	Volumetric	ETDRS-style rings + central 3 mm	Thickness	FDR correction
Vogl et al. (110)	Choroid thickness	Choroid inner boundary	Choroid-scleral boundary	Partial	Volumetric	ETDRS-style rings + central 3 mm	Thickness	FDR correction
von der Emde et al. (111)	ONL thickness	Auto-segmentation boundary (with manual correction; not explicitly mapped to named interfaces)	Auto-segmentation boundary	Partial	Multimodal (OCT + FAF + IR)	MP-aligned loci/macular ROIs	Thickness + multimodal features	Feature scaling
Wang et al. (112)	Layer thickness (ONL/PR/RPE etc.)	Manually segmented layer interfaces (study-specific)	Manually segmented layer interfaces	Full	Multimodal	Lesion-/quartile-based ROIs	Thickness + spatial correlation	FAF quartile mapping; intensity trimming
Weber et al. (113)	SDD lesion ROIs (FLIO metrics + OCT confirmation)	OCT-confirmed SDD region (ROI-based; not interface thickness)	ROI-based	Partial	Multimodal (FLIO + OCT)	Lesion ROIs	FLIO lifetime metrics + phenotype	Device-/software-specific fitting; ROI QC
Woronkowicz et al. (114)	En-face EZ/ELM loss area mapping	ELM segmentation line (multilayer segmentation)	EZ segmentation line/loss map definition	Full	En face + volumetric	ETDRS-centred analysis	Area (loss maps)	None explicitly stated
Wu et al. (116)	Complete ELM/EZ/RPE loss area (band-loss endpoints)	DL-derived band-loss maps (ELM/EZ/RPE; “complete loss” endpoint defined per band)	Not thickness-defined (area map)	Full	Volumetric	Sector-based regions	Area	Complete loss operationalised as ≤ 5 μm
Wu et al. (115)	Seven early atrophy OCT features (manual)	Manual feature measurement start (caliper-defined)	Manual feature measurement end	Full	B-scan	Lesion-focused	Length/width/ presence	Standardized reader rules + adjudication
Yang et al. (117)	Drusen morphology + overlying RPE/EZ integrity (categorical)	Qualitative grading (HEYEX)	Qualitative grading	Insufficient	B-scan	Macular ROIs	Ordinal/categorical	Categorical scales
Yordi et al. (120)	BLD; EZ attenuation; CST; SRF/IRF; SHRM volume	ML segmentation of layers + fluid compartments (interfaces not all anatomically specified in text)	Compartment-specific segmentation boundaries	Partial	Volumetric	Macular volume	% attenuation; thickness; fluid volumes	QC + exclusions
Yordi et al. (119) (J Pers Med)	EZ–RPE thickness	EZ segmentation line (DL + expert review)	RPE segmentation line	Partial	Volumetric	Macular ROIs	Thickness; % attenuation; intensity	Standardized QC; exclusions
Yordi et al. (119) (J Pers Med)	EZ attenuation (%)	EZ thickness-derived attenuation map	Not thickness-defined (percentage metric)	Partial	Volumetric	Macular ROIs	% attenuation	Standardised QC; exclusions
Yordi et al. (119) (J Pers Med)	EZ intensity	EZ band intensity ROI	Not thickness-defined	Partial	Volumetric	Macular ROIs	Intensity index	Standardized QC; exclusions
Yordi et al. (118) (Ophth Retina)	ML thickness maps + fluid volumes + volatility indices	ML layer segmentation boundaries (Cleveland Clinic; expert verification)	ML layer segmentation boundaries	Partial	Volumetric	Whole volume	Thickness; volume; volatility	Frame-by-frame verification; exclusions
Yoshida et al. (121)	EZ and RPE thickness maps; GA prediction	DL-derived EZ segmentation line	DL-derived RPE segmentation line/BM reference (model-dependent)	Partial	Volumetric + modelling	Whole volume	Prediction performance (r^2^ etc.)	Normalization/ downsampling
Zhuang et al. (122)	3D impairment volumes (EZ/ELM/ONL)	DL-derived impairment boundary (per layer)	Not thickness-defined (impairment map/volume)	Partial	Volumetric	Macular volume	Area/volume of impairment	Standardized QC of maps

Boundary Specification Adequacy was classified as: Full – Explicit anterior and posterior anatomical interfaces are clearly defined using named retinal structures (e.g., ELM–EZ, EZ–IZ, RPE–BM), allowing independent methodological replication. Partial – Boundaries are segmentation-line–based, device-defined, slab-offset–based, or composite compartments where anterior and/or posterior anatomical interfaces are implied but not explicitly defined in anatomical consensus terminology. Insufficient – A structural term is used without explicit specification of its anterior and posterior anatomical borders, or assessment is qualitative (e.g., integrity/disruption grading) without reproducible interface definition. AC1, Gwet’s agreement coefficient; AI, artificial intelligence; AMD, age-related macular degeneration; ART, automatic real-time (OCT image averaging); AUROC, area under the receiver operating characteristic curve; BCVA, best-corrected visual acuity; BLD, bacillary layer detachment; BM, Bruch’s membrane; CAM, Classification of Atrophy Meeting; CE, Conformité Européenne; CNN, convolutional neural network; CoR, coefficient of repeatability; CST, central subfield thickness; DL, deep learning; ELM, external limiting membrane; ETDRS, Early Treatment Diabetic Retinopathy Study; EZ, ellipsoid zone; EZL, ellipsoid zone loss; FAF, fundus autofluorescence; FCP, fundus-controlled perimetry; FLIO, fluorescence lifetime imaging ophthalmoscopy; GA, geographic atrophy; GLD, greatest linear dimension; HFL, Henle fiber layer; HRF, hyperreflective foci; HT, hypertransmission; ICC, intraclass correlation coefficient; iRORA, incomplete retinal pigment epithelium and outer retinal atrophy; IRF, intraretinal fluid; IS, inner segment; IZ, interdigitation zone; LLVA, low-luminance visual acuity; MAIA, Macular Integrity Assessment; ML, machine learning; MNV, macular neovascularisation; OCT, optical coherence tomography; OPL, outer plexiform layer; ORB, outer retinal bands; ORL, outer retinal layer; ORT, outer retinal tubulation; OS, outer segment; PED, pigment epithelial detachment; PR, photoreceptor; QC, quality control; RORA, retinal pigment epithelium and outer retinal atrophy; RPD, reticular pseudodrusen; RPE, retinal pigment epithelium; RMDA, rod-mediated dark adaptation; ROI, region of interest; SDD, subretinal drusenoid deposits; SD-OCT, spectral-domain optical coherence tomography; SHRM, subretinal hyperreflective material; SRF, subretinal fluid; VA, visual acuity. This table summarizes the technical measurement parameters underpinning OCT-derived photoreceptor biomarkers in AMD, including spatial scale, segmentation boundaries, thresholding logic, normalization strategies, and analytic domain. These parameters govern reproducibility, comparability, and trial readiness but are often inconsistently reported in primary studies.

Particular attention was paid to the extraction of phenotypic disease features with established biological relevance. These included drusen morphology and subtype, the presence or absence of SDD, and MNV subtype (types 1–3), where applicable. However, phenotypic stratification was frequently incomplete or absent across included studies. Key disease-defining characteristics were often inconsistently reported or not specified at all, despite substantial evidence that these features are associated with divergent biological mechanisms, structural progression patterns, and prognostic trajectories.

Drusen subtype and composition have been shown to differentially associate with retinal pigment epithelium dysfunction and photoreceptor degeneration, while SDD represents a topographically and pathophysiologically distinct entity linked to choroidal insufficiency and preferential outer retinal involvement (28–30). Similarly, MNV subtype is associated with differing natural histories, treatment responses, and risks of photoreceptor disruption. The inconsistent reporting of these phenotypes limited the feasibility of biologically meaningful subgroup analyses and may have contributed to heterogeneity in pooled estimates. Where phenotype-resolved data were available, these studies were analyzed separately; however, the small number of such studies precluded formal meta-regression.

### 2.6 Statistical analysis

Owing to substantial heterogeneity in segmentation boundaries, threshold definitions, spatial sampling frameworks, disease phenotype, and reported outcome metrics, formal quantitative meta-analysis of structural photoreceptor thickness outcomes was not performed.

Instead, findings were synthesized using a structured narrative approach with quantitative categorization of methodological variability. Operational definitions, segmentation boundaries, thresholding rules, region-of-interest strategies, and reflectivity normalization approaches were extracted and grouped into predefined anatomical and methodological domains.

Where reliability or agreement metrics [e.g., intraclass correlation coefficients (ICC), kappa statistics, and correlation coefficients] were reported, these were summarized descriptively. No pooling of effect sizes was undertaken due to differences in statistical definitions, segmentation protocols, and disease context.

Frequencies and proportions were calculated to characterize reporting completeness, boundary specification adequacy, and construct-level variability across studies.

### 2.7 Structured methodological appraisal

Although a formal risk-of-bias instrument was not applied, as no validated tool exists for the heterogeneous, construct-mapping aims of this scoping review, we conducted a structured appraisal of methodological rigor across five prespecified domains directly relevant to biomarker validity: (1) adequacy of anatomical boundary specification (full/partial/insufficient) (Table 1); (2) reliability-evidence type (formally reported vs. inferred from prior validation) (Table 2); (3) completeness of phenotypic reporting (drusen subtype, SDD/RPD status, MNV subtype); (4) specification of region-of-interest and topographic sampling; and (5) segmentation oversight and adjudication (Supplementary Table S2; summarized in Table 3). Across the 94 studies, boundaries were fully specified in 41 (44%), partially in 32 (34%), and insufficiently in 21 (22%); formal in-study reliability statistics were reported in 18 (19%), with the remainder relying on inferred reliability from previously validated pipelines. This structured appraisal provides a transparent, study-level signal of methodological rigor that contextualizes the reliability, biological-validity, and trial-readiness conclusions drawn in this review.

**TABLE 2:** Reliability evidence supporting trial readiness of OCT-derived photoreceptor biomarkers in AMD.

Study	Biomarker(s)/feature(s)	Reliability evidence type	Inter-grader (reported)	Intra-grader/test–retest (reported)	Validation/ground truth anchor	Trial-readiness interpretation
Abraham et al. (31)	EZ–RPE; ONL–RPE; EZ defect	◇ Inferred	NR	NR	Expert QC + prior validated segmentation/DL	Robust pipeline used, but reliability not quantified in-study
Bell et al. (32)	ILM–RPE; EZ–RPE; RPE–BM	• Reported	ICC 0.965–0.999 (layer-dependent)	NR	Consensus segmentation by senior certified readers	Excellent reproducibility; automation ≈ manual performance
Birner et al. (33)	EZ/ONL + drusen/HRF/SDD (DL + expert review)	◇ Inferred	NR	NR	Expert verification of all SDD	High-fidelity annotation workflow; no ICC/Dice provided
Birner et al. (34)	EZL/RPEL/EZT + morphometrics	◇ Inferred	NR	NR	Prior-validated DL + scan-level QC	Reliability supported by validated DL + QC; not reported numerically
Bogunović et al. (35)	Drusen metrics; ONL/ORB; HRF	◇ Inferred	NR	NR	Iowa algorithms + HRF training set + expert checks	Strong methodological validation; no formal ICC/Dice reported
Borrelli et al. (36)	EZ loss/OS volume; ONL thickness	• Reported	ICC (EZ) = 0.911; ICC (OS) = 0.925	NR	Manual QC review	High repeatability for volumetric EZ/OS quantification
Borrelli et al. (37)	EZ reflectivity; CC FD%	◇ Inferred	NR (prior “excellent agreement” cited)	NR	Manual reference segmentations (prior)	Automated reflectivity stable; not re-quantified here
Brandl et al. (17)	ONL; PR IS/OS; RPE/BrM	◇ Inferred (negative signal)	NR (qualitative: frequent corrections)	NR	Manual corrections used as reference	Autosegmentation error-prone in AMD; manual review essential
Carvajal et al. (38)	HRF; RPD; iRORA; cRORA	• Reported	AC1: HRF 0.88→0.92; RPD 0.82→0.89; iRORA 0.99; cRORA 0.98–0.99	NR	CAM criteria + morphology definitions	Near-perfect agreement for atrophy constructs; subtle features improved with structured scoring
Cedro et al. (39)	cRORA area; √ER; ELM/IS-OS scores	◇ Inferred	NR	NR	CAM criteria + OCT↔FAF comparison	Cross-modal agreement supports validity; reliability stats not stated
Cheung et al. (40)	RPE/EZ sinuosity; RPE/EZ continuity	◇ Inferred	NR	NR	Algorithmic segmentation + manual correction + adjudication	Reliability supported by adjudication workflow; metrics suitable for progression modeling, but no numeric reproducibility
Choi et al. (41)	IHRF, SDD, hDC, drusen volume; iRORA/cRORA	◇ Inferred	NR	NR	Reading-center grading; CAM definitions applied	Central grading supports internal validity; reproducibility not quantified in-paper
Cicinelli et al. (42)	ONL/ORL/DLS thickness; GA area (fovea-sparing GA)	• Reported	“Not reported numerically except subset ICC”	ICC 0.95–0.98(subset)	Expert adjudication + CAM criteria; OCT–FAF alignment validation	Thickness measures appear highly repeatable; qualitative features rely on adjudication
Clemens et al. (43)	dPED morphology score incl. EZ	• Reported (qualitative/BA)	“Good agreement; minimal disagreement” (BA)	NR	Manual OCT + IR review	Composite morphological scoring reproducible
Corbelli et al. (44)	GA area (FAF/en-face OCT/OCTA slabs)	• Reported	ICC 0.986–0.999 (modalities)	ICC 0.995–0.999	Manual outlining; FAF reference	Near-perfect GA area reliability across modalities
Corvi et al. (45)	iRORA/cRORA; hyperTDs	• Reported (agreement)	cRORA 97.6% agreement; iRORA 47.6%	NR	CAM + hyperTD GLD ≥ 250 μm	cRORA robust; iRORA challenging (definition + spacing constraints)
Coulibaly et al. (46)	RPEL; ELML; EZL (DL)	◇ Inferred	NR	NR	FILLY trial masks + expert correction	DL segmentation positioned for trial use; no ICC reported
Ehlers et al. (47)	OCT-GA vs. FAF-GA	• Reported (cross-modal concordance)	r≈0.92–0.93; mean Δ−0.17 to + 0.11 mm^2^	NR	FAF/OCT cross-validation + adjudication	OCT-GA robust and equivalent to FAF; endpoint-ready
Erb et al. (48)	MTII; EZII; GA area	• Reported (agreement ranges)	FAF mean Δ 0.00–0.07 mm^2^; OCT mean Δ 0.08–0.52 mm^2^	NR	Dual graders + senior adjudication; CAM criteria	Strong reproducibility for segmentation-heavy indices
Etheridge et al. (49)	Layer thickness (incl. ONL)	◇ Inferred	NR	NR	Proprietary segmentation + manual correction	Robust QC, but no ICC given
Farinha et al. (50)	7 layers + PRL	◇ Inferred	NR	NR	Full manual correction + senior QC	Reading-center grade workflow; reliability implied
Fasih-Ahmad et al. (51)	EZ/IZ area and thickness (DL + corrections)	◇ Inferred	NR	NR	Validated algorithm + multi-reader corrections	Multi-grader correction implies high reliability; not quantified
Flores et al. (52)	iRORA; EZ; HRF; drusen	• Reported (agreement %)	> 90% agreement (selected features)	NR	Two masked graders + consensus	High reproducibility for manual feature grading
Flynn et al. (53)	EZ disruption; SDD presence	◇ Inferred	NR	NR	Manual OCT grading at predefined functional loci	Structural grading stable for structure–function analysis; no formal reproducibility statistics
Fragiotta et al. (54)	ONL thickness; ORL thickness	◇ Inferred	NR	NR	Automated segmentation with manual correction; longitudinal consistency	Thickness metrics appear stable under corrected workflow; ICC not reported
Frank-Publig et al. (55)	Outer retinal band thickness/loss areas; SDDs (High-Res vs. Spectralis)	◇ Inferred	NR (cites prior improved agreement)	Prior benchmarks: mean abs error ∼1.7–3.5 μm (band-dependent)	Histology-based definitions (Project MACULA) + prior segmentation benchmarks	High-Res OCT improves boundary clarity → likely higher measurement reliability
Futterknecht et al. (56)	Focal lesion detection; lesion-targeted MP alignment	◇ Inferred	NR	NR	Alignment ground truth: mean offset 0.75° (∼218 μm)	Targeting/registration accuracy strong; functional repeatability remains lesion/type dependent
Gallagher et al. (57)	Retinal layer thickness (incl. ONL/OSL/RPE); GA hypertransmission length	◇ Inferred	NR	NR	Prior external validation of segmentation algorithm; internal repeated alignment	Reliability supported by validated segmentation; outer retinal biological variability remains limiting factor
Ghoshal et al. (58)	ONL/RPE/ORT thickness and volume	• Reported	ICC RT 0.98; ORT 0.75; ONLT 0.92; RPET 0.79 (etc.)	NR	Reader agreement	ONL robust; some outer layers more variable
Goerdt et al. (59)	28 retinal bands (HR-OCT)	• Reported	NR	κ inner 0.54–0.88; outer 0.67–0.83	Histology/EM support	Repeatable band localization; some biological visibility limits
Griffin et al. (60)	EZ reflectivity; ROI coding	• Reported	NR	ICC (EZ refl) = 0.96; κ(ROI) = 0.74	Internal consistency	High repeatability for reflectivity-based measures
Hanna et al. (61)	7 layer thickness (SS-OCT)	• Reported	ICC 0.94	NR	Manual comparison	Automated thickness mapping reliable (thin layers cautioned)
Heckenlaible et al. (62)	11 retinal layer thickness/variability/reflectivity	◇ Inferred	NR	NR	Iowa algorithms (externally validated) + manual review/corrections	Considered accurate under established algorithmic validation; no in-study ICC/κ reported
Heiferman et al. (63)	RPD/SDD identification	• Reported	ICC 0.96	NR	Manual ROI analysis	Strong reliability for RPD; semi-automated SDD reproducible
Ho et al. (64)	Tablet retinal sensitivity (PsyPad) + OCT structural mapping	• Reported	NR	CoR ∼12.3 dB (nAMD), 10.2 dB (at-risk); improves after excluding poor performers	Visual validation of OCT structure–function mapping	Functional tool repeatability quantified; OCT grading reproducibility not reported
Hong et al. (65)	SVC/DVC VD; GA size	• Reported	ICC SVC 0.813; DVC 0.891; GA 0.855	NR	Structural OCT + FAF delineation	Good–excellent reproducibility for quantitative OCTA/OCT metrics
Itoh et al. (66)	EZ volume/EZ–RPE	• Reported (test–retest)	NR	r = 0.82 for EZ volume (test–retest)	Manual segmentation check	Stable over time with QC
Kalra et al. (67) (J Pers Med)	GA/HT + multilayer (EZ/RPE/BM)	• Reported	ICC 0.88 (B-scan); 0.95 (en-face)	NR	Expert-corrected multilayer ground truth	Excellent agreement; strong trial-readiness for automated maps
Kalra et al. (68) (Diagnostics)	EZ-At-Risk; GA; bands	• Reported	ICC 0.83	NR	Triple-reader QC + adjudication	High precision, device-agnostic performance
Kar et al. (69)	EZ–RPE texture; sub-RPE shape metrics	◇ Inferred	NR	NR	ML feature stability across cross-validation	Reliability inferred from stable predictive performance rather than segmentation agreement
Liermann et al. (70)	rEZR (EZ peak reflectivity/ELM peak reflectivity)	◇ Inferred	NR	NR	Reading-center SOPs (GRADE Bonn) + prior validated automated peak-detection/segmentation + QC exclusions	Strong procedural reliability (RC + validated automation), but no in-paper reproducibility stats → moderate trial readiness
Mahmoudi et al. (71)	CAM features (HR-OCT vs. Std)	• Reported	HR-OCT ICC 0.915–0.997 vs. Std 0.518–0.601; AC1 HR 0.88 vs. Std 0.82	NR	Senior adjudication + training set	HR-OCT materially improves reliability for CAM features
Mai et al. (72)	Auto RPE/EZ loss vs. FAF GA	• Reported (cross-modal)	r 0.96–0.97	NR	FAF manual GA gold standard	OCT automation closely matches FAF; EZ extends beyond RPE loss
Mai et al. (73)	EZ/RPE loss ratio; EZ loss area; RPE loss area	◇ Inferred	NR	NR	Validated DL segmentation (device-specific) applied to phase III trial imaging + FAF/RC GA definition	Trial-embedded scalable endpoint, but reproducibility not reported in-paper → moderate–high procedural readiness
Morelle et al. (74)	Drusen/layer height (algorithm vs. expert)	• Reported	Dice (drusen) 0.62–0.71 (reader-dependent)	NR	Expert manual layers	Moderate overlap; reflects task difficulty + reader variability
Müller et al. (75)	10 OCT biomarkers incl. EZ/RPE loss	• Reported	κ 0.160–0.823; ICC 0.527–0.972; Dice 0.539–0.764	NR	Multi-reader annotation	Reliability heterogeneous; EZ/RPE loss and HRF less reliable than SRF/PED/HT
Pfau et al. (8)	Junctional phenotype class	• Reported	κ 0.77	NR	2-grader consensus	High reproducibility for phenotype classification
Pfau et al. (79) (AJO)	ONL thickness + multimodal features (GA)	◇ Inferred	“Manual segmentation corrected by two readers (no κ)”	NR	FCP function as anchor	Reliability inferred from dual-reader corrected segmentation; supports modeling but lacks numeric reproducibility
Pfau et al. (78) (JAMA)	DL multilayer + GA maps	• Reported	Dice 0.82 (95% CI 0.80–0.85)	NR	Expert manual annotations	Human-level agreement; supports endpoint extraction at scale
Pfau et al. (76) (TVST)	OCT thickness/reflectivity features (nAMD)	◇ Inferred	NR (“built on earlier validated segmentation”)	NR	Nested cross-validation; prediction MAE reported	Endpoint features operationally stable; reliability not stated as ICC/κ/Dice
Pfau et al. (77)	z-standardized ONL/IS/OS; GA trial context	◇ Inferred	NR	NR	Manual + FAF masks (trial-scale DL segmentation)	Trial-scale deployment suggests robustness; explicit reproducibility not reported
Prenner et al. (81)	RPE/PR thickness; integrity loss (High-Res vs. Spectralis; nAMD)	◇ Inferred	NR (single expert performed manual corrections/annotations)	NR	Expert annotation + validated segmentation algorithm	Improved boundary visibility reduces ambiguity; reproducibility not quantified
Qu et al. (82)	RPE vs. PR defect areas; junctional type	◇ Inferred	NR (“described as high”; dual grading + adjudication)	NR	Manual segmentation (3D-OCTOR) + adjudication	High-confidence manual framework; suitable for phenotyping; lacks numeric reliability
Riedl et al. (83)	EZ integrity (manual); IRC/SRF/PED (AI)	◇ Inferred	NR	NR	Expert manual EZ annotation; validated CNN for fluid; Iowa PED algorithm	High internal consistency via hybrid manual + validated AI; reproducibility not quantified
Riedl et al. (84)	PR thickness/ONL/drusen/HT (AI) + OPL subsidence (manual)	◇ Inferred	NR (OPL graded by 2 graders)	NR	Previously validated DL models + manual QC	Robust pipeline with manual confirmation; no ICC/κ reported for key outputs
Rogala et al. (85)	PR/RPE thickness; ONL thickness; PR thickness above drusen	◇ Inferred	NR	NR	Manual measurements with subset second-clinician verification + standardized Spectralis acquisition	Manual caliper measures with limited scalability and no stats → low–moderate readiness
Russakoff et al. (86)	PR/ONL thickness inputs; latent PR/EZ features (CNN)	◇ Inferred	NR	NR	Automated segmentation pipeline with expert verification + standardized preprocessing; outcome anchor = FA-confirmed nAMD	Procedurally robust ML pipeline, but no reproducibility stats and partly latent features → moderate methodological readiness
Sadigh et al. (87)	ONL + (incl HFL); IS + OS thickness; PR thickness over/paradrusen	◇ Inferred	NR	NR	Manual segmentation using published layer definitions + normative comparison; authors note reliability not assessed	Informative biology but manual, no reproducibility stats → low–moderate readiness
Saeed et al. (2025)	ONL thickness; ELM/EZ disruption; atrophy-associated PR features	◇ Inferred	NR	NR	CAM-based feature definitions + high-density SS-OCT QC; functional anchor = repeated microperimetry	Strong definitions + QC, but single-grader workflow and no stats → moderate readiness
Sarici et al. (89)	EZ–RPE thickness; partial/total EZ attenuation; RPE–BM thickness	◇ Inferred	NR	NR	ML-enabled multilayer segmentation + dual expert review/correction; outcome anchor = subfoveal GA conversion	Scalable quantitative EZ metrics with expert correction, but no ICC/κ/Dice → moderate–high procedural readiness
Saßmannshausen et al. (91)	ONL; PR (ELM–BM); PR-segments (ELM–RPE); RPEDC	◇ Inferred	NR	NR	Automated segmentation with full-volume manual correction + INOCT definitions + standardized Spectralis acquisition	Methodologically rigorous, but reproducibility not quantified → moderate readiness
Saßmannshausen et al. (90) (MACUSTAR)	rEZR (test–retest)	• Reported	NR	ICC overall 0.846 (0.809–0.876); stage-stratified ICCs reported	DL training + reader QC	Repeatable multicenter biomarker suitable for trials
Saßmannshausen et al. (92)	ONL/IS/OS thickness at HRF + vs. HRF− locations	◇ Inferred	NR	NR	Full-volume manual correction of segmentation + CAM/INOCT definitions; HRF graded by single experienced reader	Strong segmentation QC, but HRF single-grader + no stats → moderate readiness
Savastano et al. (93)	SRI (RORA); ONL; EZ interruption	• Reported	κ > 0.9 for ONL and EZ	NR	QC thresholds + 2 graders + fERG link	Strong reliability for ONL/EZ; SRI automated (minimal variability)
Sayegh et al. (94)	Foveal sparing area; OCT-derived GA area (outer retinal/RPE loss)	◇ Inferred	NR	NR	Vienna Reading Center SOPs + adjudication; standardized Spectralis follow-up mode	RC-grade workflow, but no reproducibility metrics and largely single-reader → moderate readiness
Schaal et al. (95)	Outer retinal tubulation (ORT)	• Reported (construct validation)	NR	NR	Direct OCT–histology correlation (light microscopy + TEM)	High biological validity; qualitative feature without reproducibility stats → low endpoint readiness (contextual/enrichment)
Schmitz-Valckenberg et al. (97)	Early atrophy features (HT, ELM/EZ, etc.)	• Reported	κ varies widely (HT/ELM moderate; EZ/ONL poor) + BA widths	NR	Senior consensus	Identifies which subtle features are unreliable without stricter definitions
Schweighofer et al. (98)	Microperimetry repeatability	• Reported	ICC MP-3 0.869; MAIA 0.848; inter-device 0.841	CoR values reported	DL-segmented OCT coregistered to MP	MP reliability strong; supports structure–function endpoint linking
Song et al. (99)	EZ/ELM + exudation features (AI vs. experts)	• Reported	Mean Dice 0.873; accuracy EZ 0.93; ELM 0.873	NR	Dual-expert consensus	Excellent algorithmic agreement for key outer-retinal bands
Steinberg et al. (100)	Partial outer retinal thickness (OPL outer border → EZ inner border)	◇ Inferred	NR	NR	Automated segmentation with full-volume manual correction + normative/topographic normalization; functional anchor = FCP	Strong methodological workflow, but no reproducibility stats → moderate readiness
Sulzbacher et al. (101)	Morphological OCT tags + ELM/EZ	• Reported	Intergrader agreement 89.5% (range 84.5–94.5%)	NR	Dual-grader certified review	High reproducibility for clinical OCT morphology grading
Inanc Tekin et al. (102)	ONL; OPL; RPE thickness	◇ Inferred	NR	NR	Dual-grader analysis + manual correction of automated segmentation; standardized Spectralis acquisition	Quantitative layers with dual grading, but no ICC/κ→ moderate readiness
Tepelus et al. (103)	RPE + OS volume; ONL volume	◇ Inferred	NR	NR	Automated segmentation with QC inspection; centralized analysis (DIRC); functional anchor = microperimetry	Scalable volumetrics with QC, but no reproducibility stats → moderate readiness
Thiele et al. (104)	rEZR (manual vs. automated)	• Reported	Manual CoR 6.3 AU; automated peak-detection 96.8%	Automated “perfect repeatability” stated	Histology-informed band definitions	Automated rEZR extraction highly reproducible for longitudinal use
Trinh et al. (108)	ONL + HFL; IS/OS; RPE–BM thickness	◇ Inferred	NR	NR	Dual-grader full-volume manual correction (ground truth) + standardized Spectralis acquisition + normative anchoring	Very strong procedural reliability, but no ICC/κ/test–retest → moderate readiness
Trinh et al. (2022)	ONL + HFL; IS/OS; RPE–BM thickness (high-density sampling)	◇ Inferred	NR	NR	Dual-grader corrected “ground truth” segmentation + high-density 60 × 60 grid + matched normals	Technically robust and scalable, but reproducibility not quantified → moderate readiness
van Romunde et al. (109)	ELM/EZ integrity and multimodal variables	• Reported	κ/ICC 0.69–0.99 for included variables	NR	Masked graders + adjudication threshold	High categorical reliability; excludes variables failing agreement
Vogl et al. (110)	ONL/ORB/choroid texture (Cirrus; atlas registration)	◇ Inferred	“High stability across > 10k scans” (qualitative)	NR	Validated segmentation + registration; clinician-defined conversion anchors	Population-scale extraction stable; reproducibility inferred (not expressed as ICC)
von der Emde et al. (111)	ONL thickness; FAF intensity; inferred sensitivity modeling	◇ Inferred	NR	NR	Microperimetry functional ground truth; manual OCT layer QC	Predictive reliability strong; segmentation reproducibility not reported numerically
Wang et al. (112)	Layer thickness (RPE/PR/ONL/choroid) + FAF/NIR correlation	◇ Inferred	NR	NR	Manual segmentation + strict co-registration	Pixel-level correspondence strong; reproducibility not reported
Weber et al. (113)	SDD lesion ROIs (FLIO metrics + OCT confirmation)	◇ Inferred	NR (single expert grader)	NR	OCT confirmation of SDD; controlled environment ROIs	Lesion-level metrics stable in practice; formal reproducibility not reported
Woronkowicz et al. (114)	EZ/ELM loss area (manual mapping)	◇ Inferred	NR	NR	Human interpretive mapping	Clinical utility shown; reproducibility stats absent
Wu et al., 2025 (longitudinal bands)	Area of complete ELM/EZ/RPE loss ( ≤ 5 μm)	• Reported	CoV: EZ 217% < ELM 244% < RPE 397%	NR	CE-marked DL segmentation; validated previously	Quantifies variability in automated band-loss endpoints (important for meta-analysis modeling)
Wu et al., 2025 (multicenter reader study)	cRORA feature set incl. EZ/ELM disruption, ONL thinning etc.	• Reported	AC1 0.80–0.88 ( ≥ 500 μm lesions)	NR	12 readers/6 certified reading centers; functional relevance	Strong trial-readiness: high inter-reader agreement when definitions sized ≥ 500 μm
Yang et al., 2021	EZ damage; RPE damage; drusen reflectivity/homogeneity/shape	• Reported	κ/agreement reported (inter-observer 83.4–95.2%)	Intra-observer agreement 82.7–94.8%	Dual masked graders + adjudication; explicit OCT morphology definitions	Strong reliability for categorical features; useful for enrichment/risk stratification (not quantitative endpoint)
Yordi et al. (120)	BLD; EZ attenuation (%0, % ≤ 20 μm); SRF/SHRM metrics	◇ Inferred	NR	NR	Trial-grade imaging + ML segmentation with masked expert correction; OSPREY phase II	Trial-embedded pipeline, but no ICC/κ/test–retest → high procedural, inferred reliability
Yordi et al. (119) (J Pers Med)	EZ attenuation (partial/total); EZ–RPE thickness/volume; EZ intensity index	◇ Inferred	NR	NR	ML segmentation + layered expert review/QC; standardized acquisition; VA prediction anchor	Strong procedural QC and longitudinal validity, but no reproducibility stats → high procedural, inferred reliability
Yordi et al. (118) (Ophthal Retina)	BLD (classic/indeterminate); EZ attenuation; EZ–RPE thickness/volume; fluid volumes	◇ Inferred	NR (dual-reader adjudicated BLD; no κ/ICC)	NR	Phase III HAWK imaging + ML quantification + adjudication	Phase III embedded and scalable, but no reproducibility stats → high procedural, inferred reliability
Yoshida et al. (121)	EZ thickness (iEZ–iRPE); RPE thickness; EZ–RPE maps; EZ/RPE intensity maps	◇ Inferred	NR	NR	ML segmentation trained on 6,718 manual labels + trial FAF grading anchor (RegionFinder)	Mature trial-grade ML pipeline, but no ICC/κ/test–retest → high procedural, inferred reliability
Zhuang et al. (122)	EZ/ELM/ONL impairment areas; avascular SHRM; MNV volume	• Reported	κ > 0.80 (two readers; arbitration if > 10% difference)	NR	Dual masked readers + explicit impairment definitions; OCT + OCTA separation of components	Explicit inter-reader reliability for 3D-derived damage metrics; manual burden limits scalability → moderate–high readiness

• Reported = explicit reliability statistic(s) provided. ◇ Inferred = reliability supported by reading-center protocols/adjudication/validated algorithms/structured QC, but no statistic reported in that paper. NR, not reported; AC1, Gwet’s agreement coefficient; AMD, age-related macular degeneration; BA, Bland–Altman; BCVA, best-corrected visual acuity; BLD, bacillary layer detachment; BM, Bruch’s membrane; CAM, Classification of Atrophy Meeting; CC, choriocapillaris; CoR, coefficient of repeatability; CoV, coefficient of variation; cRORA, complete retinal pigment epithelium and outer retinal atrophy; CST, central subfield thickness; DL, deep learning; DVC, deep vascular complex; ELM, external limiting membrane; ER, ellipsoid zone–RPE (EZ–RPE) thickness (also termed ellipsoid zone to RPE distance); EZ, ellipsoid zone; EZII, ellipsoid zone integrity index; EZL, ellipsoid zone loss; FAF, fundus autofluorescence; FA, fluorescein angiography; FD%, flow deficit percentage; FCP, fundus-controlled perimetry; GA, geographic atrophy; GLD, greatest linear dimension; HFL, Henle fiber layer; HRF, hyperreflective foci; HT, hypertransmission; ICC, intraclass correlation coefficient; iRORA, incomplete retinal pigment epithelium and outer retinal atrophy; ILM, internal limiting membrane; IRF, intraretinal fluid; IS/OS, inner segment/outer segment; IZ, interdigitation zone; MNV, macular neovascularization; MP, microperimetry; MTII, macular tissue integrity index; nAMD, neovascular age-related macular degeneration; OCT, optical coherence tomography; OCTA, OCT angiography; OPL, outer plexiform layer; ORT, outer retinal tubulation; ONL, outer nuclear layer; PR, photoreceptor; QC, quality control; RC, reading center; rEZR, relative ellipsoid zone reflectivity; RPD, reticular pseudodrusen; RPE, retinal pigment epithelium; RPE–BM, retinal pigment epithelium–Bruch’s membrane complex; RPEL, RPE loss; RPEDC, RPE–drusen complex; RORA, RPE and outer retinal atrophy; SD-OCT, spectral-domain OCT; SDD, subretinal drusenoid deposits; SHRM, subretinal hyperreflective material; SRF, subretinal fluid; SOP, standard operating procedure; SS-OCT, swept-source OCT; SVC, superficial vascular complex; VA, visual acuity.

**TABLE 3:** Structured methodological-rigor appraisal of the 94 included studies.

#	Appraisal domain	Source	Rating categories	Studies, n (%)
1	Anatomical boundary specification adequacy	Table 1	Full	41 (44%)
			Partial	32 (34%)
			Insufficient	21 (22%)
2	Reliability evidence	Table 4	Formally reported (in-study)	18 (19%)
			Inferred from prior validation	76 (81%)
3	Phenotype reporting completeness	§3.5	Qualitative appraisal*	Inconsistent
4	Region-of-interest/topographic specification	§3.3	ROI geometry specified	82 (87%)
			ROI geometry not specified	12 (13%)
			Eccentricity-/topography-stratified	29 (31%)
5	Segmentation oversight and adjudication	Supplementary Table S2	Explicit oversight	55 (59%)
			Partial oversight	25 (27%)
			None/not reported	14 (15%)

*Phenotype reporting was appraised qualitatively (§3.5): drusen presence was commonly documented, whereas drusen subtype, subretinal drusenoid deposit (SDD/RPD) status, and macular neovascularization subtype were inconsistently reported, and complete phenotype stratification was rare. A per-study rating (complete/partial/absent) can be provided in Supplementary Table S1. Percentages are of the 94 included studies; ROI, region of interest. Studies were appraised across five prespecified domains relevant to biomarker validity. A formal risk-of-bias instrument was not applied, as none is validated for the construct-mapping aims of a scoping review; this appraisal provides a transparent, study-level signal of methodological rigor.

## 3 Results

### 3.1 Study selection and characteristics

The search yielded 571 unique records after de-duplication. Following title and abstract screening, 311 full texts were reviewed, and 94 studies met the inclusion criteria and were synthesized (17, 27, 31–122) (Figure 1). The temporal distribution of publications showed a marked increase after 2020, coinciding with wider adoption of dense volumetric OCT acquisition, *en face* and three-dimensional analytic approaches, and the maturation of automated and deep-learning–based segmentation pipelines for outer retinal structures.

Included studies ranged from small exploratory cohorts to large observational datasets and trial-linked analyses, encompassing between 12 and 1,847 eyes (median 94; interquartile range 48–186) (Table 1). Most cohorts were derived from North America and Europe and were frequently associated with established academic imaging centers and AMD consortia. Heidelberg Spectralis spectral-domain OCT predominated, particularly in studies aligned with the Classification of Atrophy Meeting (CAM) framework and longitudinal geographic atrophy (GA) analyses, with Zeiss Cirrus and swept-source platforms increasingly represented in later work, especially in studies emphasizing volumetric mapping, cross-device validation, or automated analytics (70, 90). Full study characteristics are provided in Supplementary Table S1.

Disease stages were intentionally heterogeneous (Table 4). Intermediate AMD predominated (47 studies, 50%), followed by geographic atrophy or atrophic AMD including iRORA/cRORA (31, 33%), early AMD (17, 18%), and neovascular AMD (12, 13%); a further four studies (4%) addressed late dry AMD of unspecified stage. Because many cohorts deliberately spanned the AMD continuum, 18 studies (19%) contributed to more than one stage and 15 (16%) included normal or control comparators, so categories are not mutually exclusive. A substantial body of work addressed early and intermediate AMD, often focusing on subclinical outer retinal change and detailed structure–function relationships using microperimetry or dark adaptation testing (33, 53, 91, 100, 103, 110). A large GA-focused literature quantified photoreceptor degeneration within atrophic lesions and at their margins, frequently using CAM-aligned constructs (iRORA and cRORA) or closely related OCT feature definitions (45, 72, 77–80, 82, 97, 112). A smaller but clinically relevant subset examined neovascular AMD, typically in the context of treatment response, disease activity, or structure–function mapping (64, 101, 109, 111, 114, 118, 120, 122). Several studies spanned multiple disease stages to characterize biomarker behavior across the AMD continuum and to model progression risk (35, 52, 68, 69, 86, 89, 96, 121).

**TABLE 4:** Distribution of AMD phenotypes across the 94 included studies.

AMD phenotype/stage	Studies, n	% of 94 studies
Early AMD	17	18%
Intermediate AMD (iAMD)	47	50%
Geographic atrophy/atrophic AMD (incl. iRORA, cRORA)	31	33%
Neovascular AMD (nAMD)	12	13%
Late dry AMD, stage not further specified	4	4%
Mixed AMD/non-AMD or unspecified stage	3	3%
Studies including normal/control comparators	15	16%
Studies spanning > 1 disease stage	18	19%

Classification based on the AMD Stage/Phenotype field in Supplementary Table S1. Studies spanning multiple stages are counted in each applicable category; counts therefore exceed 94 and percentages exceed 100%. Control and > 1 disease stage rows are descriptive overlays, not mutually exclusive categories. cRORA, complete RPE and outer-retinal atrophy; iRORA, incomplete RPE and outer-retinal atrophy.

The use of the OCT platform reflects real-world clinical research practice. Heidelberg Spectralis was the predominant platform, used in 72 studies (77%), including 60 studies (64%) that relied exclusively on Spectralis and 12 (13%) that combined Spectralis with another OCT device. Zeiss Cirrus was the second most common platform (9%), while other devices (Topcon, RTVue, and prototype systems) each accounted for fewer than 5% of studies (17, 45, 77–80, 94, 97), followed by Zeiss Cirrus and other platforms used for cross-device evaluation or disease-agnostic validation of segmentation outputs (32, 67, 72, 90). Swept-source OCT adoption increased in later studies, particularly where deeper imaging and dense-volume acquisition supported *enface*/volumetric mapping and more automated workflows (27, 46, 62, 67, 72, 73, 121).

### 3.2 Axial quantification of photoreceptor compartments

Across the 94 included studies, “photoreceptor integrity” was most commonly inferred from axial thickness measurements of one or more outer retinal compartments. Although terminology varied, the dominant methodological assumption was that the anterior–posterior span of a defined anatomical structure reflects the quantity or preservation of photoreceptor tissue (37, 89). To reduce this ambiguity, we apply standardized terminology throughout this review (Table 5): “integrity” denotes the overarching qualitative construct, “disruption” a categorical loss of band continuity, “attenuation” a partial reduction in reflectivity or thickness, and “loss/absence” the complete absence of a detectable band, with every quantitative metric paired with its explicit boundaries and measurement domain. However, studies differed substantially in which photoreceptor compartment was measured, how its borders were defined, and how loss was operationalized. The measurement parameters underpinning each OCT-derived photoreceptor biomarker, particularly boundary choice, spatial domain, and thresholding, are summarized in Table 1.

**TABLE 5:** Standardized terminology for OCT-derived photoreceptor biomarkers.

Term	Standardized definition	Recommended usage
EZ integrity	Overarching qualitative construct denoting preservation of the ellipsoid zone band (presence, normal reflectivity, and normal thickness).	Use as a general concept only; not as a measured quantity. Avoid as a synonym for a specific metric.
EZ disruption	Categorical, focal or segmental discontinuity of the EZ band; typically continuity-graded.	Use for continuity-based (present/interrupted/absent) assessments; state grading scheme.
EZ attenuation	Partial reduction in EZ reflectivity and/or thickness without complete absence.	Use for sub-total signal/thickness reduction; specify whether reflectivity- or thickness-based.
EZ loss/EZ absence	Complete absence of a detectable EZ band (e.g., EZ–RPE = 0 μm).	Use for complete loss only; state the threshold/boundary used to define 0.
EZ thickness	Axial distance between explicitly defined anterior and posterior borders of the EZ (or EZ-referenced span).	Always state the inner and outer boundaries (e.g., ELM–EZ, EZ–RPE) and the measurement domain.
Relative EZ reflectivity (rEZR)	Normalized EZ-band intensity index referenced to a specified internal standard.	State the normalization reference (e.g., ELM-, IPL-, or peak-referenced).
ONL thickness	Axial distance from OPL to ELM; proxy for photoreceptor cell-body integrity.	State handling of the Henle fiber layer (included vs. excluded/modeled).
ELM integrity/disruption	Continuity of the external limiting membrane band.	Use disruption for categorical continuity grading; pair with EZ/ONL status in advanced disease.
Interdigitation zone (IZ)	Hyperreflective band corresponding to outer-segment–RPE apical apposition.	Report visibility/acquisition conditions; treat as emerging biomarker.
Composite outer-retinal thickness	Multi-layer axial span combining ≥ 2 photoreceptor compartments (e.g., ELM–RPE, ONL–RPE).	Always specify constituent boundaries; avoid implying single-compartment specificity.
iRORA/cRORA	Incomplete/complete RPE and outer-retinal atrophy, per CAM consensus definitions.	Use strictly per CAM; do not use as generic synonyms for EZ/ONL loss.

CAM, Classification of Atrophy Meeting; ELM, external limiting membrane; EZ, ellipsoid zone; IPL, inner plexiform layer; ONL, outer nuclear layer; OPL, outer plexiform layer; RPE, retinal pigment epithelium.

Under an anatomical framework, axial photoreceptor quantification can be grouped into three principal compartments: (1) inner segment metrics, most commonly derived from the ellipsoid zone (EZ); (2) nuclear layer metrics, typically based on outer nuclear layer (ONL) thickness; and (3) composite outer retinal spans that combine multiple layers within a single measurement.

These findings demonstrate that commonly used terms such as “EZ thickness” and “ONL loss” do not represent single anatomical constructs across the literature. Instead, identical terminology frequently corresponds to distinct segmentation boundaries, thresholding rules, and spatial sampling strategies. This heterogeneity underpins the boundary-dependent nature of axial photoreceptor biomarkers and provides context for the conditional reliability findings reported below. Operational definitions and thresholds used across studies are mapped in Table 6.

**TABLE 6:** Evidence map of operational definitions for OCT-derived photoreceptor-layer biomarkers in AMD.

Study	Biomarker(s)	Operational definition/threshold	Imaging plane	Region of interest/topography	CAM alignment
Ellipsoid zone (EZ)–based biomarkers					
Abraham et al. (31)	EZ–RPE thickness; EZ attenuation	Total EZ loss: EZ–RPE = 0 μm; partial loss ≤ 20 μm	Volumetric + en face	ETDRS subfields	Partial
Bell et al. (32)	EZ loss	EZ boundary collapsed to RPE (“EZ dropped to RPE”)	B-scan	Central macula	Partial
Birner et al. (33)	EZ thickness	EZ thickness between IZ and EZ boundaries	B-scan	70-μm radius at MP loci	Partial
Birner et al. (34)	EZ loss	Loss of EZ–IDZ band at functional loci	B-scan	70-μm radius at MP loci	Yes
Borrelli et al. (36)	EZ area/volume	Manual segmentation of EZ band between ELM and IZ	B-scan	Macular ROIs	No
Flynn et al. (53)	EZ integrity category	EZ intact/attenuated/absent (ordinal)	B-scan	Functional test loci	No
Griffin et al. (60)	EZ reflectivity	EZ reflectivity normalized to IPL reflectivity	B-scan	Pixel-based ROIs	No
Liermann et al. (70)	EZ/ELM reflectivity ratio	rEZR = EZ peak ÷ ELM peak reflectivity	Raw B-scan	Central ETDRS zones	Partial
Woronkowicz et al. (114)	EZ-loss area	En-face projection of EZ absence	B-scan → en face	Whole 20° × 20° field	Partial
Yordi et al. (120)	EZ attenuation	Partial EZ ≤ 20 μm; total EZ = 0 μm	Volumetric	Whole cube + subfields	Partial
Yordi et al. (119)	EZ attenuation/intensity	EZ thickness and intensity normalized to RPE	Volumetric	ETDRS subfields	Partial
Zhuang et al. (122)	EZ impairment	EZ absence annotated across serial B-scans	B-scan	8 × 6 mm cube	Partial
Outer nuclear layer (ONL)–based biomarkers					
Brandl et al. (17)	ONL thickness	Distance between OPL and ELM boundaries	Volumetric	ETDRS 1/3/6-mm rings	Partial
Etheridge et al. (49)	ONL thickness	Spectralis-defined OPL→ELM thickness	ETDRS maps	ETDRS grid	No
Fragiotta et al. (54)	ONL thickness	OPL outer border to ELM	ETDRS maps	Central 1 mm and 1–3 mm	Partial
Ghoshal et al. (58)	ONL thickness/volume	OPL outer border to ELM	Volumetric	Central ETDRS	No
Rogala et al. (85)	ONL thickness	ELM to posterior OPL	B-scan	Drusen-centric ROIs	No
Sadigh et al. (87)	ONL^+^ thickness	OPL vitread boundary to ELM	Line scans	6 × 6 mm raster	No
Saßmannshausen et al. (91)	ONL thickness (z-score)	ONL normalized to age-matched controls	Volumetric	56 functional loci	Partial
Thiele et al. (105)	ONL thickness	Automated ONL segmentation over time	Volumetric	ETDRS regions	Partial
Yang et al., 2021	ONL damage	Qualitative ONL disruption grading	B-scan	Drusen-associated ROIs	No
External limiting membrane (ELM)–based biomarkers					
Cedro et al. (39)	ELM integrity score	Ordinal ELM continuity score	B-scan	Central macula	Yes
Steinberg et al. (100)	Partial OR thickness	OPL to EZ inner border (adjusted if EZ absent)	Volumetric	MP loci	Partial
Sulzbacher et al. (101)	ELM integrity	Intact vs. disrupted ( > 50% interruption)	Volumetric	Pointwise loci	No
Wu et al. (2025)	ELM loss	Segmented ELM thickness ≤ 5 μm	Volumetric	Central 5 mm	Yes
Photoreceptor reflectivity defects (PRD/isolated EZ loss)					
Qu et al. (82)	PR defect type	Type 1: PR loss beyond RPE loss	B-scan	GA perimeter	No
Inanc Tekin et al. (102)	PR loss pattern	Photoreceptor loss vs. fibrotic scar comparison	B-scan	Lesion-centric	No
Composite and derived photoreceptor metrics					
Cheung et al. (40)	EZ/RPE sinuosity	Band length ÷ BM length (EZ > 1.01)	B-scan	Central macula	Partial
Erb et al. (48)	EZ integrity index	% intact EZ within 1- and 3-mm circles	En face	Central ETDRS	Yes
Pfau et al. (78)	ONL/IS/OS z-scores	Thickness normalized to eccentricity-matched controls	Volumetric	Contour-based ROIs	Partial
Pfau et al. (77)	Photoreceptor z-scores	ONL/IS/OS thickness outside GA margins	En face	Distance-based contours	Partial
Yoshida et al. (121)	Continuous PR thickness	EZ-to-RPE continuous thickness (no binary cut-off)	Volumetric	Whole cube	Partial
CAM-aligned atrophy and photoreceptor degeneration (iRORA/cRORA)					
Carvajal et al. (38)	iRORA/cRORA	CAM size thresholds (125–249 μm; ≥ 250 μm)	B-scan	ETDRS grid	Yes
Cicinelli et al. (42)	GA with foveal status	GA ≥ 0.049 mm^2^ with PR degeneration	Radial scans	Central 1 mm	Yes
Corvi et al. (45)	iRORA/cRORA	CAM criteria + hyperTD slab	B-scan + en face	6 × 6 mm	Yes
Ehlers et al. (47)	OCT-GA	RPE loss with PR degeneration confirmed vs. FAF	Volumetric	Whole lesion	Yes
Kalra et al. (67)	Automated GA	Coincident EZ, RPE and BM loss	Volumetric	6 × 6 mm	Yes
Mai et al. (72)	EZ and RPE loss	EZ ≤ 4 μm; complete RPE attenuation	Volumetric	GA within OCT FOV	Yes
Schmitz-Valckenberg et al. (97)	Early atrophy	Longitudinal iRORA/cRORA classification	Volumetric	Central macula	Yes
Wu et al. (2025)	Early atrophic lesions	CAM-based grading with reader agreement	Volumetric	3.5° region	Yes

AMD, age-related macular degeneration; BM, Bruch’s membrane; CAM, Classification of Atrophy Meetings; cRORA, complete retinal pigment epithelium and outer retinal atrophy; ELM, external limiting membrane; ETDRS, Early Treatment Diabetic Retinopathy Study; EZ, ellipsoid zone; FAF, fundus autofluorescence; GA, geographic atrophy; IZ, interdigitation zone; MP, microperimetry; ONL, outer nuclear layer; OCT, optical coherence tomography; PR, photoreceptor; PRD, photoreceptor reflectivity defect; RPE, retinal pigment epithelium.

#### 3.2.1 Photoreceptor inner segment metrics (EZ-derived)

The most common approach for quantifying photoreceptor inner segment integrity was measuring EZ thickness. In most studies, the axial thickness of the EZ hyperreflective band was used as a surrogate for preservation of inner segment structure, with thinning or absence interpreted as photoreceptor loss or dysfunction (37, 89).

However, substantial variability arose from how the EZ was defined. Some investigations measured thickness strictly between the anterior and posterior borders of the EZ band, whereas others defined spans incorporating adjacent interfaces (e.g., ELM-to-EZ or EZ-to-RPE distances). These represent distinct anatomical assumptions regarding what portion of the photoreceptor complex is being captured. Because the EZ band is thought to correspond primarily to mitochondria-rich inner segments rather than the full photoreceptor unit, interpretation of EZ thickness as total photoreceptor integrity depends critically on consistent boundary definition (15, 16).

Boundary delineation is particularly challenging in diseased maculae. In the presence of drusen, subretinal fluid, intraretinal fluid, subretinal hyperreflective material, or nascent atrophy, outer retinal bands may broaden, attenuate, or partially merge. Under these conditions, small differences in segmentation rules may materially alter measured thickness and shift classification from “attenuated” to “absent.” Inter-reader variability and segmentation ambiguity in AMD have been documented, particularly where layers are thin or poorly demarcated (32, 75).

Beyond thickness, several studies examined relative EZ reflectivity or intensity-normalized indices as potential markers of early inner segment dysfunction (90, 104). Large consortium analyses, including MACUSTAR-linked work, demonstrated stepwise reductions in relative EZ reflectivity with increasing disease severity and associations with functional outcomes (70, 90). However, reflectivity-based metrics are sensitive to acquisition parameters, signal normalization strategy, axial resolution, and post-processing pipelines, limiting direct comparability across platforms (20).

Topographic sampling further influenced interpretation. EZ measurements were reported using ETDRS subfields, central-only measures, parafoveal rings, lesion-centric masks, or microperimetry-aligned loci (33, 90). Given physiological variation in outer retinal thickness across the macula, and evidence that parafoveal regions may demonstrate earlier structural change, differences in spatial sampling constrain cross-study comparisons of absolute values (Table 6).

Collectively, inner segment metrics illustrate how differences in segmentation boundaries, thresholding rules, and spatial reporting materially affect the interpretation of ostensibly similar “EZ thickness” endpoints.

#### 3.2.2 Photoreceptor nuclear layer metrics (ONL-derived)

ONL thickness was widely used as a proxy for photoreceptor cell-body integrity and was generally defined more consistently than EZ-derived metrics. Most studies measured ONL thickness between the OPL and ELM and interpreted thinning as evidence of photoreceptor nuclear loss (17, 49, 50).

Population-based and cohort studies demonstrated progressive ONL thinning with aging and increasing AMD severity, while structure–function analyses linked ONL thickness to retinal sensitivity, reading performance, and other functional measures (54, 58, 103). These findings support the biological plausibility of ONL thickness as a marker of photoreceptor quantity.

Nevertheless, segmentation variability persisted, particularly regarding treatment of the Henle fiber layer (HFL). Most automated algorithms segment the HFL together with the ONL, thereby increasing measured thickness relative to histological definitions, whereas a smaller number of studies attempted to exclude or model HFL separately using directional or reflectivity-based approaches. Because HFL orientation is most prominent in parafoveal regions, inclusion or exclusion can materially influence measured ONL thickness and contribute to regional variability. Thus, even for nuclear-layer metrics, segmentation boundary selection remains a source of between-study inconsistency (Table 1).

Several investigations examined ONL behavior over drusen, reporting local thinning of the drusen-overlying retina and suggesting early photoreceptor compromise (87, 123). However, in early and intermediate AMD, reductions in EZ reflectivity or focal EZ disruption were often reported before frank ONL thinning, suggesting a potential temporal sequence in which inner segment dysfunction precedes nuclear loss.

#### 3.2.3 Composite outer retinal span metrics

A third category comprised composite outer retinal spans that combined multiple layers within a single anterior–posterior measurement, such as anterior ONL-to-RPE or ELM-to-RPE distances. These metrics were used to estimate overall “outer retinal thickness” or structural integrity, implicitly integrating nuclear and segmental compartments (89).

While composite measures may offer pragmatic advantages, they obscure the specific anatomical compartment driving change. Because nuclear, inner segment, and outer segment components may degenerate at different rates, combining them into a single axial span complicates biological interpretation and limits mechanistic specificity.

Recent work has also focused on atrophy-related constructs, including iRORA, cRORA, junctional-zone metrics, and hypertransmission defects (45, 97). Although hypertransmission is driven primarily by RPE attenuation or loss and frequently co-localizes with EZ and ONL abnormalities, these constructs do not directly quantify axial photoreceptor thickness. Accordingly, while highly relevant to disease staging and progression modeling, they were not considered primary axial photoreceptor integrity measures within the core framework of this review. Across compartments, axial photoreceptor quantification is therefore not a single construct, but a family of boundary-dependent ran whose interpretation depends on anatomical assumptions, segmentation rules, and spatial sampling design.

### 3.3 Methodological replicability and boundary variability

To contextualize the reliability and validity findings that follow, each study was appraised across five prespecified methodological domains (Table 3). In summary, formal in-study reliability statistics were reported in 18 studies (19%) (Table 2), region-of-interest geometry was specified in 82 (87%), with eccentricity- or topography-stratified analysis in only 29 (31%), and explicit segmentation oversight was documented in 55 (59%) (Supplementary Table S2); phenotype reporting was inconsistent (§3.5). Boundary-specification adequacy is detailed below.

Across 94 included studies, segmentation boundaries for photoreceptor biomarkers were fully and explicitly specified in 41 (44%), partially specified in 32 (34%), and insufficiently described to permit independent replication in 21 (22%) (Table 1). In these latter studies, the anatomical borders underlying thickness or loss metrics were either ambiguously described (e.g., “EZ thickness” without boundary specification) or defined using device-default terminology, without clarification of the anterior and posterior interfaces.

The EZ was the most frequently analyzed structure (*n* = 62 studies) (Table 6). However, the term “EZ thickness” or “EZ loss” encompasses at least five distinct boundary configurations, including ELM-to-EZ inner border, EZ inner-to-outer border, EZ-to-IZ, EZ-to-RPE, and composite EZ-to-RPE slabs. Reflectivity-based EZ biomarkers demonstrated further heterogeneity, with at least four normalization strategies identified, including IPL-referenced scaling, ELM-referenced ratios (rEZR), internal peak-referenced scaling, and unnormalized intensity metrics. In 28% of reflectivity-based studies, the reference standard for normalization was not specified.

Outer nuclear layer (ONL) thickness was reported in 39 studies and defined using at least four boundary configurations, most commonly OPL-to-ELM, but also including alternative OPL boundary selections and composite outer retinal slabs (Table 6). Anterior OPL border selection was not explicitly specified in approximately one-third of ONL studies. Identical anatomical boundaries were frequently labeled with different terminology, while identical terms were used to describe different boundary constructs, limiting cross-study comparability despite superficial similarity of metrics.

Threshold definitions for attenuation or loss also varied substantially. EZ loss thresholds ranged from complete collapse (0 μm) to partial attenuation defined at ≤ 20 μm, while ELM disruption was variably defined using categorical continuity grading, percentage interruption, or absolute thickness cut-offs. Hypertransmission size thresholds for atrophy varied between CAM-aligned definitions and non-size-based constructs. In 37% of studies employing binary thresholds, the rationale for cut-off selection was not provided.

Region-of-interest (ROI) strategies were similarly heterogeneous. Twenty-nine studies employed ETDRS subfields, 18 used lesion-centric or contour-based ROIs, 21 applied custom macula-wide slabs, 14 used pointwise microperimetry-aligned ROIs, and 12 did not explicitly define ROI geometry. Only 31% of studies explicitly incorporated eccentricity-adjusted or topography-stratified analyses, despite known macular thickness gradients.

Collectively, these findings demonstrate that axial photoreceptor quantification represents a family of boundary-dependent surrogates rather than a single construct. This construct instability has direct implications for AI validation, reproducibility, and interpretation of imaging-derived trial endpoints. The scale of boundary, threshold, and ROI variability provides a quantitative explanation for cross-study heterogeneity and reinforces the central conclusion of this review: reproducibility is high when segmentation conventions are explicitly defined and consistently applied, but methodological comparability is limited when anatomical boundaries are incompletely specified.

### 3.4 Segmentation methodology and reliability evidence

Of the 94 included studies, 18 (19%) formally reported inter- or intra-grader reliability statistics for photoreceptor-layer metrics. Among studies reporting intraclass correlation coefficients (ICCs) for continuous measures (e.g., ONL thickness, EZ–RPE span), values ranged from 0.87 to 0.97, with the majority exceeding 0.90 under prespecified segmentation protocols and adequate image quality. Agreement was lower for categorical constructs such as partial EZ disruption or transitional atrophic states, particularly in morphologically complex AMD. Reliability evidence supporting trial readiness, including reported vs. inferred reproducibility, is summarized in Table 2.

Because axial photoreceptor metrics depend critically on boundary placement, segmentation methodology represents a central determinant of reliability. Across the included literature, segmentation approaches evolved from manual grading and device-native software with extensive correction to custom research pipelines and fully automated or deep-learning–based algorithms. Earlier studies and anatomically complex cases frequently relied on manual or semi-automated workflows to adjudicate subtle outer retinal bands, whereas later investigations increasingly adopted automated pipelines to enable dense volumetric analysis, longitudinal modeling, and scalability to trial-sized datasets (37, 43, 78, 85).

Where formally reported, inter- and intra-grader agreement for quantitative EZ and ONL thickness measurements was generally high under constrained conditions, specifically when segmentation rules were prespecified, image quality was high, and analyses were restricted to eyes with relatively preserved outer retinal architecture. However, reproducibility declined in the presence of thin layers, gradual transition zones, or complex AMD-related pathology, including drusen, nascent atrophy, intraretinal or subretinal fluid, and subretinal hyperreflective material (17, 75, 97). In these settings, ambiguity in defining anterior and posterior borders, particularly at the EZ–RPE interface or within attenuated ONL regions, was a major contributor to intergrader variability.

Cross-reader and cross-disease validation studies demonstrated good agreement for EZ- and ONL-based metrics in non-atrophic or structurally simpler disease contexts (32). However, this performance did not consistently generalize to AMD cohorts characterized by progressive thinning, band merging, or evolving atrophy. Comparisons using high-resolution OCT further underscored that axial resolution, scan averaging, and preprocessing strategies influence delineation of outer retinal bands and atrophic lesion boundaries, with direct implications for both reliability and sensitivity to change (55, 71).

Automated segmentation pipelines were increasingly applied not only for structural quantification but also for prognostic modeling and treatment-response analyses, including prediction of progression to late AMD and evaluation of therapeutic effects in phase 3 datasets (35, 72, 86, 89, 96). While these approaches enhance scalability and consistency, multiple studies emphasized the continued need for manual review or adjudication in AMD to mitigate segmentation failure modes arising from thin, ambiguous, or out-of-distribution morphology (75, 97). Segmentation pipelines, oversight, and adjudication workflows are detailed in Supplementary Table S2.

Taken together, reliability evidence suggests that axial photoreceptor thickness metrics are reproducible when anatomical boundaries are clearly defined and outer retinal architecture is preserved. However, in advanced or morphologically complex AMD, precisely where such biomarkers are most clinically relevant, segmentation ambiguity becomes a principal source of variability. Device-specific acquisition parameters and processing pipelines further modulate performance and are summarized in Supplementary Table S3.

### 3.5 Phenotypic context and reporting completeness

Interpretation of axial photoreceptor metrics is further influenced by phenotypic context. Across studies, reporting of biologically relevant modifiers was inconsistent, limiting phenotype-resolved interpretation of thickness-derived biomarkers.

Drusen presence was commonly documented, but detailed characterization of drusen subtype, burden, and internal composition was variably reported. Several investigations demonstrated associations between drusen-associated morphology and local photoreceptor alteration, and highlighted the feasibility of AI-based drusen segmentation to support quantitative phenotyping (74, 85, 87, 123). Because drusen can distort overlying outer retinal architecture, failure to account for drusen morphology may confound the interpretation of axial thickness metrics.

Subretinal drusenoid deposits (SDD), also known as reticular pseudodrusen (RPD), were frequently mentioned but inconsistently quantified. SDD are associated with characteristic topographic patterns of degeneration and rod dysfunction, and have been shown to exhibit distinct spatial distributions on en face OCT compared with infrared imaging (63, 91, 124). Importantly, SDD and interdigitation zone alterations may alter apparent layer boundaries and reflectivity profiles. In studies employing EZ-to-RPE or other composite outer retinal spans, these features can introduce additional variability in thickness estimates even in the absence of overt photoreceptor cell-body loss.

Reporting of macular neovascularization (MNV) subtype was also uneven. While some neovascular AMD studies stratified outcomes by lesion type or specific morphological features, others did not clearly distinguish between subtypes (118, 120). Because fluid, subretinal hyperreflective material, and bacillary layer changes can distort outer retinal bands, inconsistent phenotype reporting limits cross-study synthesis of photoreceptor biomarkers in neovascular contexts.

Overall, incomplete phenotypic characterization complicates the interpretation of axial thickness metrics, particularly where segmentation boundaries are already sensitive to morphological distortion. Standardized reporting of drusen phenotype, SDD presence, and neovascular subtype would enhance comparability and biological interpretability of photoreceptor integrity measures. CAM adoption and atrophy-definition frameworks are summarized in Supplementary Table S4.

### 3.6 Structure–function relationships

Structure–function analysis formed a central component of the reviewed literature. Functional endpoints included mesopic and scotopic microperimetry, best-corrected visual acuity (BCVA), low-luminance acuity, electrophysiological measures, and performance-based outcomes such as reading speed (54, 58, 79, 91). These associations are summarized by AMD stage, biomarker type, and functional endpoint in Table 7, which reports the direction and strength of reported relationships and representative studies; values are reported ranges and representative findings rather than pooled estimates.

**TABLE 7:** Structure–function relationships by AMD stage, biomarker type, and functional endpoint.

AMD stage	Biomarker type	Functional endpoint	Reported association (direction/strength)	Representative studies (ref. #)
Early/intermediate AMD	EZ (reflectivity, thickness, focal disruption)	Mesopic and scotopic microperimetry (pointwise sensitivity)	Moderate–strong; stepwise reduction in relative EZ reflectivity with severity; DL-derived EZ metrics associated with local sensitivity loss even without overt atrophy	(6, 60, 89, 91)
Early/intermediate AMD	ONL thickness	Microperimetry, contrast sensitivity, reading performance	Thinner ONL → reduced sensitivity; evidence of non-linear/threshold relationship (preserved function until critical thinning)	(34, 38, 91, 108)
Early/intermediate AMD	Composite/partial outer-retinal thickness (incl. RPD)	Scotopic and mesopic fundus-controlled perimetry; deep sensitivity	Correlated with deep/scotopic sensitivity loss; stronger than high-contrast acuity for parafoveal change	(33, 86, 87, 103)
Early/intermediate AMD	EZ/ONL	BCVA	Generally weak; BCVA insensitive to parafoveal/extrafoveal loss in early disease	(68)
Geographic atrophy	EZ loss/disruption	Pointwise microperimetry	Strong local coupling; sensitivity decrements frequently > 10 dB at EZ-disrupted loci; residual function within lesions and pronounced loss at margins	(7, 34, 73, 74, 75)
Geographic atrophy	ELM + EZ + ONL (composite)	BCVA; cone- and rod-mediated function	ELM disruption with EZ/ONL loss → worse VA; combined photoreceptor + RPE features best explain functional variance	(15, 74)
Geographic atrophy	EZ–RPE/outer-retinal thickness	Deep visual sensitivity	OCT-defined atrophic change associated with deep visual-sensitivity losses (multicentre)	(86, 122)
Neovascular AMD	EZ and ELM integrity	BCVA; retinal sensitivity; treatment response	EZ/ELM integrity prognostic for visual outcome; AI morphology features predict function and activity	(100, 104, 117, 120, 121)
Across stages (synthesized)	EZ/ONL (overall)	Microperimetry sensitivity	*r* ≈ 0.50–0.80 (stronger in advanced disease)	Results §3.6; Abstract
Across stages (synthesized)	EZ/ONL (overall)	BCVA	*r* ≈ 0.40–0.70 (context-dependent)	Results §3.6; Abstract

Values are reported ranges and representative findings synthesized across methodologically heterogeneous studies; they are not pooled effect sizes. Correlation strength is influenced by microperimetry grid design, test density, spatial-alignment strategy, boundary definition, and disease stage. BCVA, best-corrected visual acuity; DL, deep learning; EZ, ellipsoid zone; ELM, external limiting membrane; ONL, outer nuclear layer; RPD, reticular pseudodrusen; RPE, retinal pigment epithelium.

Microperimetry was particularly informative because it enabled topographic alignment between OCT-derived photoreceptor metrics and pointwise retinal sensitivity (33, 54, 91). In intermediate AMD, deep-learning–derived structural metrics demonstrated associations with local sensitivity loss even in the absence of overt atrophy (33, 84). These findings support the biological plausibility of axial thickness and reflectivity metrics as markers of functional impairment.

However, the strength and spatial specificity of structure–function correlations varied with microperimetry grid design, test density, and alignment strategy. Lesion-targeted or adaptive microperimetry grids highlighted limitations of standard sampling approaches for focal pathology and underscored the importance of spatial congruence when relating structural biomarkers to functional outcomes (56). Thus, variability in topographic sampling, already identified as a source of structural heterogeneity, extends directly to functional correlation analyses.

In GA, spatially resolved studies demonstrated residual function within atrophic areas and pronounced sensitivity loss at lesion margins (8, 79, 80), reinforcing the clinical relevance of high-resolution OCT mapping. These findings also suggest that photoreceptor degeneration may extend beyond clinically defined lesion borders, depending on the structural definition applied.

BCVA was widely reported but generally less sensitive to parafoveal or extrafoveal photoreceptor loss, particularly in earlier disease stages (10). Nonetheless, associations between outer retinal integrity and BCVA were observed in specific contexts, including foveal-sparing GA and treatment-response analyses (39). Low-luminance and deep-sensitivity measures more consistently captured functional deficits not reflected in high-contrast acuity and showed stronger associations with parafoveal or eccentric structural change (51, 53, 125).

Taken together, structure–function evidence supports the biological validity of axial photoreceptor metrics. However, the magnitude and localization of these associations depend on precise spatial alignment, consistent boundary definition, and phenotypic context. Variability in these methodological parameters likely contributes to between-study differences in reported correlation strength.

### 3.7 Agreement between OCT- and FAF-based atrophy assessment

A subset of studies directly compared OCT-based atrophy constructs with FAF-derived GA measures. Across cohorts, strong correlations were observed between OCT- and FAF-based GA area, with high sensitivity and specificity for lesion detection (44, 47). Large trial-linked analyses demonstrated close correspondence between automated OCT-derived GA measurements and conventional FAF endpoints, supporting OCT as a feasible modality for GA monitoring in clinical trials (44, 47, 72).

However, the magnitude of agreement depended on the operational definitions used, particularly the thresholds for defining atrophy onset and lesion margins across modalities. Differences in whether atrophy was defined by complete RPE and outer retinal loss (e.g., cRORA) or by FAF hypoautofluorescence influenced both lesion area estimates and margin delineation (45, 78, 79).

Importantly, OCT–FAF discordance was biologically informative rather than contradictory. Structural OCT frequently identified outer retinal thinning, EZ loss, and subsidence preceding the development of definitive hypoautofluorescence, suggesting a structural lead time, particularly at lesion margins and in nascent atrophy (45, 78, 79). Conversely, FAF may detect metabolic RPE dysfunction that precedes complete photoreceptor loss or does not meet strict OCT-based atrophy criteria (45, 78).

A recent phase 3 trial–linked analysis directly compared automated SD-OCT–derived geographic atrophy measurements with conventional FAF-based endpoints and demonstrated high overall agreement in total lesion area (72). However, systematic differences emerged at lesion margins, where modality-specific definitions of atrophy and differing sensitivity to early structural change influenced boundary delineation. OCT-based definitions, grounded in axial outer retinal and RPE structural loss, frequently identified subtle subsidence or photoreceptor attenuation not yet meeting FAF hypoautofluorescence criteria. Conversely, FAF sometimes detected metabolic RPE dysfunction without complete structural collapse on OCT.

These findings suggest that apparent discrepancies between OCT and FAF are not simply measurement error, however reflect differences in the biological compartment being captured—axial outer retinal structural integrity on OCT versus RPE metabolic status on FAF. Together, these data support a complementary, multimodal framework for atrophy assessment, in which OCT provides direct structural quantification of nuclear and inner segment compartments, while FAF reflects RPE functional integrity. Harmonization of atrophy definitions across modalities, including explicit specification of structural thresholds and boundary criteria, will be essential to ensure consistent endpoint interpretation in both observational studies and interventional trials.

### 3.8 Imaging protocols and adoption of standardization frameworks

Imaging acquisition protocols varied substantially across studies in terms of scan density, field size, averaging strategies, motion correction, and reporting transparency. Earlier investigations frequently relied on lower-density raster scans and device-native acquisition settings, whereas more recent studies increasingly adopted high-density volumetric protocols to enable en face mapping, three-dimensional reconstruction, and deep-learning–based segmentation workflows. Such methodological shifts have direct implications for axial layer delineation, particularly in thin or transitional zones where sampling density and signal averaging influence boundary detection. Device models, acquisition protocols, and QC thresholds across studies are summarized in Supplementary Table S3.

Adoption of the Classification of Atrophy Meeting (CAM) definitions for atrophy was evident in an increasing proportion of the literature and was associated with improved inter-reader agreement and longitudinal consistency, particularly for lesion definition and progression endpoints (24, 97). The CAM framework provides explicit structural criteria for atrophy classification, thereby reducing interpretive variability at lesion borders.

However, for photoreceptor-specific biomarkers, no equivalent consensus framework exists. Many studies continued to employ custom segmentation rules, device-specific algorithms, or hypothesis-driven composite measures of outer retinal thickness. While such innovation has advanced quantitative analysis and prognostic modeling, it has also introduced heterogeneity in layer definitions, thresholding rules, and spatial sampling strategies. This tension between methodological innovation and the need for harmonization is particularly salient for axial photoreceptor metrics, where small variations in acquisition parameters or segmentation rules can materially influence measured thickness.

Collectively, the literature demonstrates increasing standardization of atrophy endpoints, but continued variability in photoreceptor-layer quantification. Establishing clearer reporting standards, including explicit specification of acquisition parameters, segmentation boundaries, and topographic sampling frameworks, will be essential for cross-study comparability and for the translation of axial photoreceptor biomarkers into robust clinical trial endpoints. These gaps motivate a minimum reporting framework (Table 8).

**TABLE 8:** Minimum reporting framework for OCT-derived photoreceptor biomarkers in AMD.

Domain	Required reporting elements	Rationale
1. Anatomical definition and segmentation boundaries	Explicit anterior and posterior boundaries for each biomarker (e.g., EZ–RPE, OPL–ELM) Inclusion/exclusion of adjacent layers (e.g., Henle fiber layer) Criteria for attenuation vs. complete loss Handling of transitional/ambiguous bands	Boundary choice materially alters quantitative outputs; explicit definition prevents construct drift across studies.
2. Imaging platform and acquisition parameters	Device manufacturer and model Software version and segmentation algorithm (native vs. custom vs. DL) Scan protocol (field size, raster density, averaging, eye tracking) Axial/lateral resolution (if relevant) Quality control thresholds and exclusion criteria	Device architecture and acquisition parameters influence band visibility, thickness measurements, and reflectivity metrics.
3. Analytic domain and measurement strategy	Measurement domain (B-scan, en face, slab, volumetric) Units (thickness, area, volume, reflectivity index) Preprocessing steps (flattening reference, interpolation, motion correction) Handling of ungradable regions or missing data	Analytic strategy determines spatial sampling and sensitivity to focal vs. diffuse degeneration.
4. Reflectivity and intensity normalization	Reference structure used for normalization (e.g., RPE, vitreous, internal band) Thresholding logic for defect definition Signal strength adjustments Statement on inter-device comparability	Reflectivity-based biomarkers are device- and signal-dependent; transparent normalization reduces measurement artefact.
5. Region of interest (ROI) and topographic reference	ROI definition (ETDRS grid, central 1 mm, lesion-centric mask, whole macula) Inclusion/exclusion of lesion margins Spatial alignment method (fovea-centered, MP-aligned, etc.) Biological justification for ROI choice	Spatial sampling strongly influences structure–function correlations and progression rates.
6. Phenotypic Stratification	Drusen subtype/burden Presence of SDD MNV subtype (if applicable) Atrophy classification framework (e.g., CAM iRORA/cRORA)	Photoreceptor metrics behave differently across phenotypes; stratification improves interpretability and external validity.
7. Reliability and validation	Type of reliability statistic (ICC, κ, Dice, CoR) Sample size used for reliability testing Inter- and/or intra-grader assessment Cross-device validation (if applicable) Adjudication procedures	High reliability is conditional on clearly defined boundaries and consistent analytic rules.
8. Longitudinal and Progression Modeling (if applicable)	Definition of progression event Inter-scan interval Handling of segmentation drift Sensitivity analyses for boundary ambiguity	Longitudinal metrics are particularly vulnerable to boundary instability and algorithm drift.

AMD, age-related macular degeneration; CAM, Classification of Atrophy Meetings; CoR, coefficient of repeatability; DL, deep learning; ETDRS, Early Treatment Diabetic Retinopathy Study; EZ, ellipsoid zone; ICC, intraclass correlation coefficient; iRORA, incomplete retinal pigment epithelium and outer retinal atrophy; cRORA, complete retinal pigment epithelium and outer retinal atrophy; MNV, macular neovascularisation; OCT, optical coherence tomography; OPL, outer plexiform layer; RPE, retinal pigment epithelium; ROI, region of interest; SDD, subretinal drusenoid deposits; κ, kappa statistic. To facilitate harmonization and regulatory translation, we propose a minimum reporting framework for OCT-derived photoreceptor biomarkers structured to address the principal sources of construct variability identified in this review.

## 4 Discussion

This scoping review demonstrates that OCT-derived photoreceptor-layer biomarkers in AMD are not a single construct, but a family of anatomically distinct, boundary-dependent axial surrogates, most commonly derived from the ellipsoid zone and outer nuclear layer. Across disease stages, these measures demonstrate analytical robustness and biological relevance when segmentation conventions are clearly specified, spatial sampling is standardized, and quality control procedures are applied. However, their interpretation depends critically on how anatomical borders are defined, how spatial sampling is performed, and how transitional states are operationalized. Recognizing these dependencies is essential to distinguishing biological signal from methodological artifact and to advancing photoreceptor metrics toward regulatory-grade application.

### 4.1 Analytical validity and reliability

Across studies reporting formal reproducibility outcomes, inter- and intra-rater reliability for quantitative photoreceptor biomarkers was consistently high, particularly for continuous measures such as ONL thickness, EZ–RPE thickness, and OCT-derived GA area. Among the subset of studies that formally reported inter-grader reliability statistics, intraclass correlation coefficients (ICCs) for continuous thickness measures consistently exceeded 0.90, supporting their suitability for group-level comparisons and longitudinal assessment. However, the majority of included studies relied on inferred reliability based on previously validated segmentation pipelines or established reading-center protocols, without reporting in-study reproducibility metrics (17, 32, 75). In contrast, agreement was lower for categorical or transitional constructs, including partial EZ disruption and early incomplete RPE and outer retinal atrophy (iRORA), which explicitly incorporates hypertransmission defects and subtle outer retinal changes, a pattern consistently observed across intergrader studies (38, 97), particularly at lesion margins where hypertransmission, EZ attenuation, and ONL thinning may not yet co-localize. This reduced agreement likely reflects the intrinsic subtlety and boundary ambiguity in early structural transition of OCT-based assessment.

The literature also documents a clear methodological evolution from manual and semi-automated segmentation to fully automated, deep-learning–based pipelines. Quantitative validation studies demonstrate that contemporary automated approaches achieve agreement comparable to, and in some cases exceeding, expert manual grading for EZ and ONL boundaries (32, 68). High Dice similarity coefficients and excellent test–retest reliability have been reported across diverse retinal diseases, including AMD, supporting scalability to large observational cohorts and multicenter trials. However, generalizability remains dependent on the composition of the training data, imaging platform heterogeneity, and critically, the alignment and stability of the ground-truth anatomical definitions used for validation. Several studies have noted performance degradation when algorithms were applied across devices or populations not represented in the training set, reinforcing the importance of external validation and human-in-the-loop quality control (75, 96).

From a practical grading and acquisition perspective, operability constraints represent a major determinant of observed variability, particularly for reflectivity- and continuity-based EZ metrics. In routine clinical and trial imaging, EZ intensity and reflectivity are influenced by optical media clarity, pupil size, focus, signal strength, and scan quality, such that apparent “dimming” of the EZ may reflect acquisition factors rather than biological change. Motion artifact, poor fixation, decentration, and shadowing from overlying pathology can further distort the appearance and continuity of the outer retinal band. In neovascular AMD, intraretinal or subretinal fluid and subretinal hyperreflective material may render the EZ intermittently ungradable or spuriously variable across visits. Automated pipelines introduce additional dependencies on preprocessing choices, including flattening reference, slab definition, reflectivity normalization, and thresholding, which can materially alter measured EZ extent or thickness and limit portability across devices and sites. These operability considerations help explain why high reproducibility is most consistently observed under constrained conditions and underscore the continued need for prespecified rules, quality control, and expert adjudication in both manual and automated analyses.

Collectively, these findings indicate that photoreceptor biomarkers can satisfy key criteria for analytical validation when segmentation rules are explicitly defined, consistently applied, and supported by appropriate quality control and phenotype-aware interpretation. Automated and AI-enabled approaches appear well-suited to augment expert oversight in regulatory-grade applications, but current evidence does not support their use as complete replacements for human adjudication in pivotal trials.

### 4.2 Biological plausibility and structure–function coupling

A central and unifying finding of this review is the consistency and biological plausibility of structure–function relationships linking photoreceptor biomarkers to visual outcomes. EZ integrity, operationalized across studies as loss or attenuation of the EZ band, reductions in EZ reflectivity, or decreases in EZ thickness, area, or volume, emerged as the most informative single correlate, demonstrating moderate-to-strong associations with both global functional measures and spatially resolved outcomes such as pointwise retinal sensitivity (78, 124). Spatially resolved analyses consistently showed large decrements in retinal sensitivity, often exceeding 10 dB, at loci with EZ disruption, supporting a direct local coupling between photoreceptor structure and function (54, 80).

ONL thickness provided complementary information, particularly as a marker of photoreceptor cell-body loss. Numerous cohort and population-based studies demonstrated progressive ONL thinning with increasing AMD severity and aging (17, 50). Structure–function analyses further linked ONL thinning, defined either in absolute terms or relative to age-matched normative reference data, to reduced retinal sensitivity, contrast sensitivity, and reading performance (54, 58). Importantly, spatially resolved structure–function analyses suggested potential non-linear relationships between ONL thickness and functional sensitivity, with relative preservation of function at higher thickness ranges and steeper decline once thinning progressed beyond a critical threshold (33, 54, 91, 103).

ELM status and composite outer retinal metrics refined structure–function associations, particularly in advanced disease and perilesional regions. In GA, disruption of the ELM in conjunction with EZ and ONL loss was associated with worse visual acuity and more pronounced functional impairment, supporting its role as a marker of advanced photoreceptor degeneration (39, 79). Deep-learning–based analyses further demonstrated that combined photoreceptor and RPE features best explained variability in cone- and rod-mediated function, reinforcing a layered and stage-dependent model of structure–function coupling (33, 79).

Together, these data support a conceptual framework in which EZ, ONL, and ELM metrics provide complementary rather than interchangeable information, capturing different aspects of photoreceptor integrity across the AMD spectrum.

### 4.3 Comparison with established endpoints and multimodal imaging

The high concordance between OCT- and fundus autofluorescence (FAF)-based assessment of GA provides quantitative support for OCT as a valid alternative or complement to FAF in clinical trials. Multiple studies reported strong correlations between OCT-derived GA area and FAF-defined lesions, with high sensitivity and specificity for lesion detection (47, 72). Trial-linked analyses from the OAKS and DERBY studies further demonstrated close correspondence between automated OCT-derived GA measurements and conventional FAF endpoints, supporting the feasibility of OCT-based monitoring in phase 3 trials (72), particularly when consistent operational definitions and lesion-boundary thresholds were applied across modalities.

Importantly, OCT–FAF discordance was consistently shown to be biologically informative rather than contradictory. Longitudinal studies demonstrated that OCT can identify photoreceptor loss, outer retinal subsidence, and early cRORA-like change at lesion margins and in nascent atrophy before the development of definitive hypoautofluorescence, suggesting a structural lead time (45, 78). Conversely, FAF may detect metabolic RPE dysfunction that precedes complete photoreceptor loss or does not meet strict OCT-based atrophy criteria (115). The relative timing and magnitude of these signals depend on the specific atrophy definitions and thresholds applied, underscoring that OCT and FAF probe complementary aspects of the degenerative process. These complementary strengths argue strongly for multimodal imaging strategies in both trials and high-resolution observational studies.

### 4.4 Implications for clinical trials and regulatory science

From a regulatory science perspective, candidate structural endpoints must demonstrate analytical validity, biological relevance, sensitivity to change over trial-relevant intervals, and interpretability in relation to patient benefit (23). Across these domains, EZ- and ONL-based OCT biomarkers address many criteria for early-phase and enrichment endpoints in AMD; however, regulatory interpretation remains dependent on harmonized construct definitions and transparent validation frameworks, particularly when definitions are prespecified, segmentation boundaries are harmonized, and phenotypic context is incorporated into analytical frameworks (10, 11), and may also serve as eligibility or enrichment criteria to define at-risk populations or exclude eyes with advanced, non-modifiable photoreceptor loss, provided that segmentation conventions, phenotype context, and analytic definitions are prespecified and consistently applied.

Longitudinal evidence indicates that photoreceptor biomarkers not only reflect contemporaneous function but also predict subsequent visual decline. Several studies demonstrated that baseline EZ or ONL integrity and rates of photoreceptor loss were associated with future development of GA, foveal involvement, or visual acuity decline (42, 52, 78). These associations were most robust when biomarkers were derived from anatomically relevant regions and interpreted in the context of disease stage and phenotype, aligning photoreceptor metrics with conceptual frameworks for candidate surrogate endpoints, biomarkers reasonably likely to predict clinical benefit, pending confirmation in interventional trials.

Photoreceptor biomarkers also offer opportunities for risk enrichment and patient stratification, enabling identification of eyes at higher risk of progression based on baseline structural vulnerability. In established GA, preservation of perilesional photoreceptors may provide treatment-responsive outcomes complementary to GA area alone, particularly for therapies targeting early disease mechanisms (77, 96), and may enhance sensitivity to detect treatment effects over shorter trial durations.

### 4.5 CLEAR framework: standardization priorities and future directions

The heterogeneity observed across studies underscores that analytical robustness is contingent upon boundary stability and harmonized operational definitions. Despite a strong overall signal, substantial heterogeneity in biomarker definitions, thresholds, and regions of interest remains a major barrier to adoption. EZ metrics alone encompassed dozens of operational definitions across the literature, while ONL boundaries and spatial sampling strategies varied widely. Phenotypic stratification by drusen subtype, subretinal drusenoid deposit status, and macular neovascularization subtype was inconsistently reported, limiting biologically meaningful synthesis (27, 63), and constraining the use of photoreceptor biomarkers as reproducible eligibility or enrichment criteria across trials.

The success of the Classification of Atrophy Meeting (CAM) framework provides a clear model for extending consensus to photoreceptor biomarkers (24, 126, 127). The CLEAR initiative was conceived as a complementary effort focused on harmonizing photoreceptor-layer constructs, AI validation strategies, segmentation conventions, and reporting standards for OCT-derived biomarkers in AMD. Priorities include agreement on segmentation boundaries, preferred regions of interest, measurement units, thresholds for partial versus complete disruption, validation datasets, and minimum reporting requirements for AI-enabled biomarker quantification, with explicit consideration of how these definitions will be applied for both trial eligibility, enrichment, and longitudinal endpoints, aligned with INOCT nomenclature (16). Routine ETDRS-based reporting, mandatory phenotypic context, and standardized reliability reporting would substantially enhance cross-study synthesis, facilitate regulatory acceptance, and accelerate translation into clinical trials. Exploratory evidence implicating Müller-cell–associated structures, including ELM-related metrics and reflectivity-based surrogates, is summarized in Supplementary Table S4 and should be interpreted as hypothesis-generating rather than endpoint-ready.

### 4.6 Emerging photoreceptor biomarkers

Beyond EZ and ONL metrics, several emerging biomarkers may refine photoreceptor characterization. Interdigitation zone (IZ) integrity, reflecting the apposition of outer segments to RPE apical processes, is increasingly resolvable with high-resolution, high-density OCT and may be among the earliest bands to attenuate; however, its visibility is acquisition-dependent, and inter-reader concordance remains to be established (55, 59, 81). Photoreceptor reflectivity profiles, including directional (Stiles–Crawford-related) EZ reflectivity and relative EZ reflectivity (rEZR), provide intensity-based surrogates of inner-segment health that show a stepwise decline with disease severity and associations with function, but are sensitive to acquisition parameters and normalization strategies (60, 70, 90, 104, 105). Outer retinal hyperreflective foci (HRF), implicated in RPE migration and intraretinal cellular changes, carry prognostic value for progression and may modify the interpretation of overlying photoreceptor metrics (92). Finally, adaptive optics and high-magnification imaging enable *in vivo* quantification of cone density and mosaic regularity, providing cellular-scale validation of axial OCT surrogates, particularly in eyes with subretinal drusenoid deposits (27, 106). These biomarkers are currently heterogeneous in definition and limited in evidence of reproducibility, and we therefore regard them as hypothesis-generating priorities for the CLEAR harmonization agenda rather than trial-ready endpoints.

### 4.7 CLEAR framework implications

A central finding of this review is that many OCT-derived photoreceptor biomarkers currently function as boundary-dependent analytical constructs rather than universally standardized anatomical entities. This has important implications for AI validation and regulatory science because algorithm performance metrics are inherently dependent on the underlying construct definition used as the reference standard. Apparent differences in algorithm performance may therefore reflect instability or inconsistency in the endpoint definition itself rather than true differences in segmentation capability.

The CLEAR initiative (Consensus Layer Evaluation for AI Algorithm Reporting) was developed to address these challenges through harmonization of segmentation conventions, biomarker terminology, reporting standards, and validation approaches for AI-enabled OCT biomarkers in AMD. The present manuscript serves as CLEAR Report 1 by defining the conceptual and methodological landscape underpinning photoreceptor biomarker interpretation and reproducibility.

### 4.8 Strengths and limitations

This review integrates operational definitions, reliability evidence, structure–function associations, and cross-modal agreement, providing a comprehensive assessment of OCT-derived photoreceptor biomarkers not previously available. Strengths include a broad, systematic search, adherence to PRISMA-ScR guidance (128), dual data extraction, and quantitative synthesis where feasible. Limitations primarily reflect the underlying literature, including inconsistent reporting, modest sample sizes in some cohorts, and limited phenotype-stratified data. As a scoping review, a formal risk-of-bias assessment was not performed because the primary objectives were to map the existing literature, clarify key concepts and operational definitions, identify knowledge gaps, and determine whether a more focused systematic review with certainty grading is currently feasible; no validated risk of bias instrument exists for this construct mapping aim. To ensure that our conclusions regarding reliability, biological validity, and trial readiness were not drawn uncritically, we instead conducted a structured appraisal of methodological rigor across five prespecified domains (§2.7), which provides a transparent, study-level signal of methodological quality in place of a formal risk-of-bias score.

## 5 Conclusion

In summary, OCT-derived photoreceptor biomarkers, particularly EZ and ONL metrics, can be reproducible, biologically meaningful, and closely linked to visual function when segmentation boundaries and analytic conventions are clearly specified and consistently applied. When defined and applied in a fit-for-purpose manner, these biomarkers can support multiple trial functions, including eligibility and enrichment strategies, risk stratification, and sensitive outcome assessment beyond conventional visual acuity.

These measures demonstrate the evidentiary foundation necessary for candidate trial endpoints, but their translation to regulatory-grade application requires consensus on segmentation standards, phenotype reporting, and cross-platform validation; steps that the heterogeneity documented in this review makes both necessary and achievable. Continued consensus-building, modeled on the CAM framework and explicitly extending to photoreceptor-specific constructs, will be critical to harmonizing definitions, reducing operability-related variability, and realizing the full translational and regulatory potential of photoreceptor biomarkers in AMD. CLEAR Report 1 provides the conceptual and evidentiary foundation for future efforts focused on AI validation, harmonized reporting standards, and regulatory-grade photoreceptor imaging endpoints.
