# Supplementary material for: CLEAR report 1: a scoping review and meta-analysis for definitions, imaging metrics, and functional correlates of photoreceptor integrity in AMD
Source: Front Med (Lausanne). 2026 Jul 6;16:1578813. doi: 10.3389/fmed.2026.1887548 (PMC13382513; doi:10.3389/fmed.2026.1887548)
Supplement: Supplementary file 1 [file Data_Sheet_1.docx]

# **Supplemental Appendix**

**Definitions, Measurement Constructs, and Functional Interpretation of OCT-Derived Photoreceptor Biomarkers in AMD: A Scoping Review with Conceptual Framework**

**Kimberly L. Spooner,** PhD^a,b,c^**; Anjali Gaston,** MD^d^; **Anushka Irodi**, MD^e^; **Alicia Lim^c^**, MD; **Livia Faes,** MD, PhD^c,f,g,h^**; Sobha Sivaprasad,** MD, PhD^c^; **Dun Jack Fu,** MD, PhD^c^;

Table of Contents

[Supplemental Appendix 1](#_Toc223373398)

[1. Search and Screening Documentation 2](#_Toc223373399)

[Supplemental Appendix 1. Details of the Search Strategy 2](#_Toc223373400)

[**Databases Searched** 2](#_Toc223373401)

[PubMed (MEDLINE) Search Strategy 2](#_Toc223373402)

[Embase (Ovid) Search Strategy 3](#_Toc223373403)

[Supplemental Appendix 2. Screening and Eligibility Criteria 4](#_Toc223373404)

[Inclusion Criteria 4](#_Toc223373405)

[**Population** 4](#_Toc223373406)

[**Exposure / Index Measurement** 4](#_Toc223373407)

[**Outcomes** 4](#_Toc223373408)

[**Study Design** 4](#_Toc223373409)

[Exclusion Criteria 4](#_Toc223373410)

[Screening Process 5](#_Toc223373411)

[**Stage 1: Title and Abstract Screening** 5](#_Toc223373412)

[**Stage 2: Full-Text Screening** 5](#_Toc223373413)

[Summary of Screening Flow 5](#_Toc223373414)

[Supplemental Appendix 3. PRISMA 2020 Main Checklist 6](#_Toc223373415)

[Supplemental Appendix 4. PRISMA Abstract Checklist 9](#_Toc223373416)

[Supplemental Appendix 5: Excluded Full-Text Studies With Reasons 10](#_Toc223373417)

[Supplemental Table S1. Study Characteristics 14](#_Toc223373418)

[Supplemental Table S2. Segmentation Pipelines and Analytical Approaches Used for OCT-Derived Photoreceptor Metrics in Age-Related Macular Degeneration 22](#_Toc223373419)

[Supplementary Table S3 — Device & Protocol Specifications 29](#_Toc223373420)

[Supplementary Table S4 — CAM Adoption & Atrophy Definitions 36](#_Toc223373421)

[Supplementary Table S5 — Müller Cell / Gliosis Evidence (Exploratory) 41](#_Toc223373422)

## 1. Search and Screening Documentation

## Supplemental Appendix 1. Details of the Search Strategy

**Databases Searched**

- PubMed/MEDLINE
- Embase (Ovid)
- Scopus
- Cochrane Library (Trials)

No study-design limits were applied in the primary strategy. Searches were restricted to:

- English language
- Publication date: January 1, 2015 to current
- Human studies

The final search was conducted on 11 January, 2026.

**PubMed (MEDLINE) Search Strategy**

("Tomography, Optical Coherence"[Mesh] OR
 "Angiography, Optical Coherence Tomography"[Mesh] OR
 OCT[tiab] OR "optical coherence tomograph*"[tiab] OR
 SD-OCT[tiab] OR "spectral domain OCT"[tiab] OR
 SS-OCT[tiab] OR "swept source OCT"[tiab] OR
 OCTA[tiab] OR "OCT angiograph*"[tiab])

AND

("Macular Degeneration"[Mesh] OR
 "Geographic Atrophy"[Mesh] OR
 AMD[tiab] OR ARMD[tiab] OR
 "age-related macular degeneration"[tiab] OR
 "intermediate AMD"[tiab] OR
 "geographic atrophy"[tiab] OR
 GA[tiab] OR
 iRORA[tiab] OR cRORA[tiab] OR
 "retinal pigment epithelium and outer retinal atrophy"[tiab])

AND

("Photoreceptors"[Mesh] OR
 photoreceptor*[tiab] OR
 "ellipsoid zone"[tiab] OR EZ[tiab] OR
 "external limiting membrane"[tiab] OR ELM[tiab] OR
 "outer nuclear layer"[tiab] OR ONL[tiab] OR
 "outer retinal layer"[tiab] OR
 reflectivit*[tiab] OR
 thickness[tiab] OR
 segmentation[tiab])

Limits applied:

- English
- 2015–Current
- Humans

**Embase (Ovid) Search Strategy**

1 exp optical coherence tomography/ OR exp optical coherence tomography angiography/
2 (OCT OR SD-OCT OR SS-OCT OR OCTA OR "optical coherence tomograph*").ti,ab.
3 1 OR 2

4 exp age related macular degeneration/
5 exp geographic atrophy/
6 (AMD OR ARMD OR "age-related macular degeneration" OR "intermediate AMD" OR GA OR iRORA OR cRORA).ti,ab.
7 4 OR 5 OR 6

8 exp photoreceptor cell/
9 ("ellipsoid zone" OR EZ OR "external limiting membrane" OR ELM OR "outer nuclear layer" OR ONL OR reflectivit* OR segmentation OR thickness).ti,ab.
10 8 OR 9

11 3 AND 7 AND 10
12 limit 11 to yr="2015 - Current"
13 limit 12 to English language
14 limit 13 to human

Duplicates were removed using Covidence and manual verification.

## Supplemental Appendix 2. Screening and Eligibility Criteria

**Inclusion Criteria**

Studies were included if they met all of the following:

**Population**

- Adults with age-related macular degeneration (early, intermediate, neovascular, or geographic atrophy)
- Human subjects only

**Exposure / Index Measurement**

- Quantitative or categorical SD-OCT-derived photoreceptor or outer retinal biomarkers, including:
  - Ellipsoid zone (EZ) integrity or thickness
  - EZ–RPE or ELM–EZ metrics
  - Outer nuclear layer (ONL) thickness
  - External limiting membrane (ELM) integrity
  - Reflectivity-based metrics (e.g., rEZR)
  - CAM-defined atrophy features (iRORA, cRORA)

**Outcomes**

- Structural quantification
- Reliability/reproducibility outcomes
- Structure–function correlations
- Longitudinal progression
- Prognostic modelling
- Trial-based endpoints

**Study Design**

- Prospective or retrospective cohort studies
- Randomised controlled trials (post-hoc imaging analyses)
- Cross-sectional validation studies
- Deep-learning segmentation validation studies
- Multicentre or registry analyses

**Exclusion Criteria**

- Case reports (<10 eyes)
- Review articles, editorials, or commentaries
- Conference abstracts without full data
- Animal or histopathological-only studies
- Studies without quantitative OCT photoreceptor metrics
- Non-English publications
- Pre-2015 publications

**Screening Process**

Screening was conducted in Covidence (Veritas Health Innovation, Melbourne, Australia).

**Stage 1: Title and Abstract Screening**

Two independent reviewers screened all retrieved records.

**Stage 2: Full-Text Screening**

Full texts were assessed independently by two reviewers.
Disagreements were resolved by consensus.

Final study selection is illustrated in the PRISMA flow diagram (Figure 1).

**Summary of Screening Flow**

- Records identified: 571
- Duplicates removed: 207
- Screened: 360
- Full texts assessed: 311
- Excluded at full text: 217
- Studies included in qualitative synthesis: 94

## Supplemental Appendix 3. PRISMA 2020 Main Checklist

| **Section and Topic** | **Item #** | **Checklist item** | **Location where item is reported** |
| --- | --- | --- | --- |
| **TITLE** |  |  |  |
| Title | 1 | Identify the report as a systematic or scoping review. | Title Page |
| **ABSTRACT** |  |  |  |
| Abstract | 2 | See the PRISMA 2020 for Abstracts checklist. | Manuscript (Abstract) |
| **INTRODUCTION** |  |  |  |
| Rationale | 3 | Describe the rationale for the review in the context of existing knowledge. | Manuscript – Introduction |
| Objectives | 4 | Provide an explicit statement of the objective(s) or question(s) the review addresses. | Manuscript – Introduction (Final Paragraph) |
| **METHODS** |  |  |  |
| Eligibility criteria | 5 | Specify the inclusion and exclusion criteria for the review and how studies were grouped. | Manuscript – Methods – Eligibility Criteria; Supplemental Appendix 2 |
| Information sources | 6 | Specify all databases and sources searched and the date last searched. | Manuscript – Methods – Search Strategy |
| Search strategy | 7 | Present the full search strategies, including filters and limits used. | Supplemental Appendix 1 – Search Strategy |
| Selection process | 8 | Describe screening methods, number of reviewers, independence, and automation tools used. | Manuscript – Methods – Study Selection |
| Data collection process | 9 | Describe data extraction methods and reviewer processes. | Manuscript – Methods – Data Extraction |
| Data items | 10a | List and define all outcomes for which data were sought. | Manuscript – Methods – Definitions and Outcomes |
|  | 10b | List and define other variables extracted (study design, device, segmentation method, etc.). | Manuscript – Methods – Data Extraction; Tables 1–3 |
| Study risk of bias assessment | 11 | Specify methods used to assess risk of bias. | Manuscript – Methods – Risk of Bias Assessment; Supplemental Risk-of-Bias Tables |
| Effect measures | 12 | Specify effect measures used. | Not applicable – no quantitative meta-analysis performed (scoping review) |
| Synthesis methods | 13a | Describe how studies were grouped for synthesis. | Manuscript – Methods – Data Synthesis Strategy |
|  | 13b | Describe preparation of data for synthesis. | Manuscript – Methods – Data Charting and Standardisation |
|  | 13c | Describe tabulation and visual presentation methods. | Tables 1–4; Supplementary Tables S1–S5 |
|  | 13d | Describe methods used to synthesise results and rationale. | Manuscript – Methods – Narrative Synthesis |
|  | 13e | Describe methods to explore heterogeneity (if applicable). | Not applicable – no formal meta-analysis conducted |
|  | 13f | Describe sensitivity analyses (if conducted). | Not applicable |
| Reporting bias assessment | 14 | Describe methods used to assess reporting bias. | Not assessed – Not applicable |
| Certainty assessment | 15 | Describe methods used to assess certainty of evidence. | Not formally assessed – scoping review methodology |
| **RESULTS** |  |  |  |
| Study selection | 16a | Describe results of search and selection process; include flow diagram. | Manuscript – Results 3.1; Figure 1 (PRISMA Flow Diagram) |
|  | 16b | Cite excluded studies that appeared eligible and explain exclusions. | Supplemental Appendix – Excluded Studies Table |
| Study characteristics | 17 | Cite each included study and present its characteristics. | Table 1; Supplementary Table S1 |
| Risk of bias in studies | 18 | Present risk-of-bias assessments. | Supplemental Risk-of-Bias Tables |
| Results of individual studies | 19 | Present summary findings of individual studies. | Results Sections 3.2–3.8; Tables 1–3; Supplementary Tables S1–S5 |
| Results of syntheses | 20a | Summarise characteristics of contributing studies. | Results 3.2–3.8 |
|  | 20b | Present results of syntheses. | Results 3.2–3.8; Tables 2–3 |
|  | 20c | Present heterogeneity investigations. | Descriptive only – Results 3.3 and 3.8 |
|  | 20d | Present sensitivity analyses. | Not applicable |
| Reporting biases | 21 | Present assessments of reporting bias. | Not assessed – Not applicable |
| Certainty of evidence | 22 | Present certainty assessments. | Not formally assessed (scoping review design) |
| **DISCUSSION** |  |  |  |
| Discussion | 23a | Provide general interpretation in context of other evidence. | Manuscript – Discussion |
|  | 23b | Discuss limitations of included evidence. | Manuscript – Discussion – Limitations |
|  | 23c | Discuss limitations of review process. | Manuscript – Discussion – Methodological Considerations |
|  | 23d | Discuss implications for practice, policy, and research. | Manuscript – Discussion – Standardisation and Regulatory Implications; Table 4 |
| **OTHER INFORMATION** |  |  |  |
| Registration and protocol | 24a | Provide registration information. | Manuscript – Methods – Registration (state if not registered) |
|  | 24b | Indicate where protocol can be accessed. | PROSPERO (if registered) or Not applicable |
|  | 24c | Describe protocol amendments. | Not applicable |
| Support | 25 | Describe funding sources and roles. | Manuscript – Funding Statement |
| Competing interests | 26 | Declare competing interests. | Manuscript – Competing Interests |
| Availability of data | 27 | Report availability of data, materials, and forms. | Manuscript – Data Availability Statement |

## Supplemental Appendix 4. PRISMA Abstract Checklist

| **Topic** | **No.** | **Item** | **Reported?** |
| --- | --- | --- | --- |
| **TITLE** |  |  |  |
| Title | 1 | Identify the report as a systematic or scoping review. | Yes |
| **BACKGROUND** |  |  |  |
| Objectives | 2 | Provide an explicit statement of the main objective(s) or question(s) the review addresses. | Yes |
| **METHODS** |  |  |  |
| Eligibility criteria | 3 | Specify the inclusion and exclusion criteria for the review. | Yes |
| Information sources | 4 | Specify the information sources (e.g., databases, registers) used to identify studies and the date when each was last searched. | Yes |
| Risk of bias | 5 | Specify the methods used to assess risk of bias in the included studies. | Not formally assessed (scoping review design; stated in Methods) |
| Synthesis of results | 6 | Specify the methods used to present and synthesize results. | Yes |
| **RESULTS** |  |  |  |
| Included studies | 7 | Give the total number of included studies and summarize relevant characteristics. | Yes |
| Synthesis of results | 8 | Present results for main outcomes. If meta-analysis was done, report summary estimate and confidence interval. | Yes (narrative synthesis; no meta-analysis) |
| **DISCUSSION** |  |  |  |
| Limitations of evidence | 9 | Provide a brief summary of limitations of the evidence included. | Yes (abstract discussion sentence included) |
| Interpretation | 10 | Provide a general interpretation of the results and important implications. | Yes |
| **OTHER** |  |  |  |
| Funding | 11 | Specify the primary source of funding for the review. | Yes (Funding statement) |
| Registration | 12 | Provide the register name and registration number. | Yes |

## Supplemental Appendix 5: Excluded Full-Text Studies With Reasons

Full-text articles excluded after eligibility assessment are listed below with primary reasons for exclusion. Reasons reflect prespecified criteria requiring extractable quantitative performance, reproducibility, or prognostic data for OCT-derived photoreceptor biomarkers in AMD.

| **First Author** | **Year** | **Journal** | **Primary Reason for Exclusion** |
| --- | --- | --- | --- |
| Ahmed | 2016 | *Molecular Vision* | Animal / preclinical model of geographic atrophy; not human OCT photoreceptor biomarker study |
| Azoadahnaf | 2024 | *European Journal of Ophthalmology* | CNN-based AMD classification; no quantitative photoreceptor biomarker outcomes |
| Chatziralli | 2017 | *Seminars in Ophthalmology* | Quality-of-life study; no structural OCT photoreceptor metrics |
| Chen | 2021 | *Ophthalmology Retina* | Histopathologic correlation of FAF; not in vivo OCT-derived biomarker analysis |
| Edwards | 2025 | *J Integr Neurosci* | Animal (mouse) aging study; not human OCT photoreceptor biomarker study |
| Fleckenstein | 2010 | *IOVS* | Pre-2015 publication (outside date restriction) |
| Goel | 2022 | *Journal of Clinical Medicine* | Hyperreflective foci segmentation study; not focused on photoreceptor-layer biomarkers |
| Guymer | 2023 | *Ophthalmology Retina* (Editorial) | Editorial commentary; no original imaging data |
| Heine | 2025 | *TVST* | AI segmentation study; not reporting clinical photoreceptor biomarker outcomes |
| Jaffe | 2021 | *Ophthalmology Retina* | Consensus classification report; no original quantitative photoreceptor biomarker data |
| Jamil | 2025 | *TVST* | High-resolution OCT of normal aging; not AMD cohort and no clinical photoreceptor biomarker outcomes |
| Kim | 2022 | *Scientific Reports* | Functional optoretinography in mice; preclinical dark adaptation model |
| Lamin | 2019 | *Eye* | Retinal layer volumetrics in early/intermediate AMD; not specific to photoreceptor integrity biomarkers of interest |
| Lee | 2020 | *TVST* | OCT hardware development study; no clinical biomarker outcomes |
| Li | 2019 | *Retina* | Clinicopathologic GA correlation; histology-based, not quantitative in vivo photoreceptor biomarker analysis |
| Linderman | 2020 | *Ophthalmology Retina* | FAZ morphology study; unrelated to photoreceptor-layer OCT biomarkers in AMD |
| Lindner | 2018 | *IOVS* | GA border morphology and progression; not photoreceptor-layer biomarker study |
| Litts | 2016 | *IOVS* | Outer retinal tubulation analysis; structural description without extractable quantitative biomarker framework |
| Lujan | 2024 | *Retina* | Directional OCT imaging technique study; exploratory reflectivity analysis without extractable quantitative photoreceptor biomarker outcomes |
| Midena | 2021 | *Frontiers in Immunology* | Diabetic retinopathy cohort; hyperreflective retinal foci methodology study, not AMD photoreceptor-layer biomarker study |
| Monés | 2013 | *Ophthalmology* | Foveal sparing in GA; evaluates apparent ONL swelling, not a biomarker harmonization or quantitative photoreceptor-layer outcomes study |
| Moradi | 2023 | *Computers in Biology and Medicine* | Deep-learning segmentation and AMD classification study; focuses on algorithm performance rather than clinical photoreceptor biomarker validation |
| Mukherjee | 2022 | *Biomedical Optics Express* | 3D deep learning segmentation in AMD; methodological segmentation development study without clinical biomarker outcome analysis |
| Nusinowitz | 2018 | *Current Eye Research* | Pre-clinical AMD structural study; no standardized quantitative photoreceptor biomarker framework aligned with prespecified criteria |
| Ouyang | 2013 | *Graefes Arch Clin Exp Ophthalmol* | Descriptive phenotypes of OPL/Henle fiber layer reflectivity; not AMD photoreceptor biomarker quantification |
| Paavo | 2017 | *IOVS* | Reticular pseudodrusen multimodal imaging study; structural correlation without standardized quantitative photoreceptor biomarker framework |
| Pappuru | 2011 | *IOVS* | Manual segmentation study correlating outer retinal thickness with VA; pre-2015 and not aligned with prespecified biomarker definitions |
| Puell | 2019 | *Current Eye Research* | Healthy aging study; no AMD cohort and no disease-specific photoreceptor biomarker analysis |
| Rajan | 2021 | *Indian Journal of Ophthalmology* | Pupillometry study correlating RAPD with CNVM lesion size; functional pupillary outcomes, not OCT photoreceptor biomarker analysis |
| Ramkumar | 2018 | *Retina* | Inner retinal (ganglion cell layer) volume analysis in GA; not focused on photoreceptor-layer biomarker definitions |
| Reiter | 2025 | *Ophthalmology and Therapy* | Practical imaging review of GA; narrative review without extractable primary biomarker data |
| Ristau | 2013 | *Ophthalmologica* | Correlative study of VA and OCT volumes in neovascular AMD; not focused on standardized photoreceptor biomarker definitions or reproducibility outcomes |
| Ross | 2015 | *IOVS* | Automated reflectivity band modeling methodology; pre-2015 and technical segmentation study rather than clinical biomarker validation |
| Sadda (CAM Report 3) | 2018 | *Ophthalmology* | Consensus classification paper defining OCT-based atrophy terminology; not a primary outcome biomarker study |
| Sadigh | 2013 | *IOVS* | Drusen–photoreceptor thickness relationship in intermediate AMD; exploratory morphologic study without standardized biomarker performance framework |
| Savastano | 2014 | *IOVS* | Automated segmentation of multiple retinal layers in early ARM; inner retinal emphasis and not prespecified photoreceptor biomarker validation |
| Simader | 2014 | *American Journal of Ophthalmology* | GA progression monitoring study comparing SD-OCT with FAF; focused on atrophy metrics rather than photoreceptor-layer biomarker definitions |
| Sumaroka | 2025 | *TVST* | Inherited retinal degeneration cohort; non-AMD population |
| Trinh | 2024 | *Ophthalmology Retina* | Systematic review/meta-analysis of OCT prognostic biomarkers; not primary data study |
| Wang | 2023 | *Current Eye Research* | Deep learning disease detection framework; classification study without extractable quantitative photoreceptor biomarker metrics |
| Wong | 2019 | *TVST* | Neurodegenerative disease cohort (non-AMD); segmentation validation study unrelated to AMD photoreceptor biomarkers |
| Yu | 2020 | *Biomedical Optics Express* | OCT-based GA growth biomarker development; focused on HFL-ONL projections rather than photoreceptor biomarker standardization |
| Zhou | 2025 | *Biomedical Optics Express* | AO-OCT cone detection/IS-OS measurement framework; methodological imaging study without AMD clinical biomarker outcomes |

## Supplemental Table S1. Study Characteristics

| **Study (Author, Year)** | **Design** | **Eyes (n)** | **AMD Stage / Phenotype** | **OCT Platform** | **Temporal Scope** | **Primary Analytic Intent** |
| --- | --- | --- | --- | --- | --- | --- |
| Abraham et al., 2022 | Post-hoc RCT analysis | 45 | Intermediate non-neovascular AMD | Spectralis SD-OCT | ~7 months | OCT-derived photoreceptor and outer retinal change in a clinical-trial cohort |
| Bell et al., 2024 | Cross-sectional validation | 210 | Mixed AMD and non-AMD | Cirrus & Spectralis SD-OCT | Single visit | Cross-device validation of automated OCT layer segmentation |
| Birner et al., 2024 | Prospective cross-sectional | 20 | Intermediate AMD | Spectralis SD-OCT | Single visit | Structure–function correlation of photoreceptor metrics |
| Birner et al., 2025 | Prospective cross-sectional | 20 | Geographic atrophy (cRORA) | Spectralis SD-OCT | Single visit | Structure–function correlation within geographic atrophy |
| Bogunović et al., 2017 | Prospective longitudinal | 61 | Early and intermediate AMD | Spectralis SD-OCT | 15–63 months mean 37.8 ± 13.8months) | Longitudinal modelling of OCT features predicting progression |
| Borrelli et al., 2019 | Prospective cross-sectional | 35 | Intermediate AMD ± RPD | Spectralis SD-OCT | Single session | Repeatability of photoreceptor metrics after photobleaching |
| Borrelli et al., 2020 | Retrospective case–control | 70 | Intermediate AMD | PLEX Elite SS-OCT | Single visit | Automated EZ reflectivity and vascular associations |
| Brandl et al., 2019 | Population-based cross-sectional | 822 | No AMD to early AMD | Spectralis SD-OCT | Single visit | Accuracy of automated vs manually corrected segmentation |
| Carvajal et al., 2024 | Cross-sectional agreement study | 79 | Intermediate AMD | Spectralis SD-OCT | Single visit | Inter-grader agreement of CAM-defined atrophy features |
| Cedro et al., 2023 | Retrospective longitudinal | 204 | Geographic atrophy | Spectralis SD-OCT + FAF | Up to 10 years | Longitudinal OCT–FAF concordance and GA progression |
| Cheung et al., 2024 | Retrospective longitudinal | 59 | Intermediate AMD | Spectralis & Cirrus SD-OCT | ~4 years | Prognostic value of RPE and EZ continuity metrics |
| Choi et al., 2025 | Retrospective longitudinal | 101 | Intermediate AMD | Cirrus SD-OCT | 24 months | Longitudinal analysis of CAM-defined atrophy features |
| Cicinelli et al., 2024 | Retrospective longitudinal | 167 | Non-exudative GA | Spectralis SD-OCT + FAF | ~4 years | Photoreceptor and outer retinal thinning in GA |
| Clemens et al., 2015 | Cross-sectional | 26 | Drusenoid PED | Spectralis SD-OCT | Single visit | Structure–function assessment in drusenoid PED |
| Corbelli et al., 2017 | Prospective reliability | 47 | Geographic atrophy | SD-OCT, FAF, OCT-A, Cirrus AngioPlex | Single visit | Inter-modality and inter-reader reliability of GA measures |
| Corvi et al., 2023 | Cross-sectional comparison | 91 | Late atrophic AMD | Spectralis & Cirrus SD-OCT | Single visit | Topographic agreement of CAM atrophy across OCT |
| Coulibaly et al., 2023 | Retrospective analysis | 74 | Geographic atrophy | Spectralis SD-OCT | 18 months | Deep-learning segmentation of photoreceptor loss |
| Ehlers et al., 2024 | Post-hoc trial analysis | 260 | Geographic atrophy | SD-OCT + FAF | 18 months | Multimodal validation of OCT-derived GA metrics |
| El Ghazi et al., 2024 | Prospective cross-sectional | 24 | Intermediate AMD with SDD | Spectralis SD-OCT + HMM | Single visit | Characterisation of photoreceptor disruption in SDD |
| Erb et al., 2025 | Retrospective longitudinal | 43 | Geographic atrophy | Spectralis SD-OCT + FAF | 12 months | Reproducibility and functional relevance of OCT indices |
| Etheridge et al., 2021 | Population-based cohort | 906 | No AMD to late AMD | Spectralis SD-OCT | Single visit | Retinal layer thickness associations with visual function |
| Farinha et al., 2021 | Population-based cross-sectional | 346 | Early AMD | Spectralis SD-OCT | Single visit | Early AMD–related retinal layer characterisation |
| Fasih-Ahmad et al., 2024 | Cross-sectional | 476 | Normal to intermediate AMD | Spectralis SD-OCT | Baseline only | Photoreceptor integrity and dark adaptation deficits |
| Flores et al., 2023 | Prospective cohort | 135 | Intermediate AMD | Spectralis SD-OCT | 24 months | OCT biomarkers associated with progression to late AMD |
| Flynn et al., 2018 | Cross-sectional | 42 | Early/intermediate AMD | Spectralis SD-OCT + FAF + NIR | Single visit | Mapping early photoreceptor dysfunction to function |
| Fragiotta et al., 2022 | Retrospective longitudinal | 18 | Intermediate AMD | Spectralis SD-OCT + Zeiss Plex Elite | 12 months | Multimodal structure–function evaluation |
| Frank-Publig et al., 2025 | Cross-sectional comparison | 40 | Geographic atrophy | Spectralis SD-OCT + High Resolution | Single visit | Photoreceptor delineation using high-resolution OCT |
| Futterknecht et al., 2025 | Prospective observational | 93 | Intermediate AMD with focal lesions | Spectralis-type SD-OCT | Same-day sessions | Lesion-targeted structure–function assessment |
| Gallagher et al., 2022 | Multicentre retrospective | 17 | GA, fovea-involving | Spectralis SD-OCT +FAF ± FA | ≥4 years | Retinal layer and hypertransmission quantification in fovea-involving GA |
| Ghoshal et al., 2020 | Cross-sectional | 26 | Early and intermediate AMD | Spectralis SD-OCT | Single visit | Reproducibility of retinal layer thickness measures |
| Goerdt et al., 2024 | Cross-sectional validation | 65 | Healthy to intermediate AMD | High-resolution Spectralis SD-OCT | Single visit | Validation of retinal band segmentation and nomenclature |
| Griffin et al., 2021 | Retrospective analysis | 37 | Intermediate AMD and GA | Directional SD-OCT using Zeiss Cirrus 4000 HD-OCT | Single visit | Repeatability of EZ reflectivity and contour metrics |
| Hanna et al., 2021 | Retrospective cohort | 37 | Treated intermediate AMD | SS-OCT (Topcon Triton) | 6 months | Reliability of automated OCT segmentation |
| Heckenlaible et al., 2025 | Retrospective longitudinal | 50 | Early to intermediate AMD | Spectralis SD-OCT | 3.6 ± 2.0 y | Longitudinal photoreceptor layer change assessment |
| Heiferman et al., 2015 | Retrospective cross-sectional | 54 | AMD with RPD | Cirrus & Spectralis SD-OCT | Single visit | OCT features associated with reticular pseudodrusen |
| Ho et al., 2018 | Prospective cross-sectional | 74 | Neovascular AMD + fellow eyes | Spectralis SD-OCT | Single visit | Structure–function correlation in nAMD |
| Hong et al., 2022 | Retrospective comparative | 28 | GA vs fellow iAMD | SD-OCT | ~25 months | Comparative OCT biomarker profiling in GA versus fellow intermediate AMD |
| Itoh et al., 2016 | Retrospective validation | 34 | Outer retinal disease (incl. GA) | Bioptigen & Cirrus SD-OCT | Single visit | Validation of ellipsoid-zone mapping techniques |
| Kalra et al., 2022 | Retrospective AI development | 341 | Non-exudative AMD ± GA | Spectralis + Cirrus SD-OCT | Cross-sectional | Development of AI-based OCT feature detection |
| Kalra et al., 2023 | Retrospective longitudinal | 461 | Non-exudative AMD ± GA | Spectralis + Cirrus SD-OCT | ~60 months | Longitudinal validation of AI-derived OCT biomarkers |
| Kar et al., 2024 | Retrospective longitudinal | 137 | Intermediate/advanced dry AMD | Spectralis SD-OCT | 60 months | Prediction of subfoveal GA conversion |
| Liermann et al., 2025 | Cross-sectional | 275 | Controls to late AMD | Spectralis SD-OCT | Single visit | Reflectivity-based photoreceptor integrity metrics across AMD stages |
| Mahmoudi et al., 2024 | Prospective cross-sectional | 60 | AMD (none/iRORA/cRORA) | High-Res OCT vs Spectralis SD-OCT | Single visit | Comparison of high-resolution versus conventional OCT boundary detection |
| Mai et al., 2024 | Post-hoc RCT analysis | 719 | Geographic atrophy | SD-OCT + FAF | 24 months | Trial-based analysis of OCT-derived atrophy metrics |
| Mai et al., 2025 | Post-hoc DL analysis | 889 | Geographic atrophy | Spectralis SD-OCT | 24 months | Deep-learning quantification of photoreceptor loss |
| Morelle et al., 2023 | AI development/validation | 134 volumes* | Early–intermediate AMD | SD-OCT (Spectralis/Bioptigen) | Cross-sectional | Development and internal validation of AI-based OCT segmentation pipelines |
| Müller et al., 2021 | Cross-sectional reliability | 112 | nAMD, iAMD, late AMD | Topcon SD-OCT | Single visit | Inter-reader reliability of OCT-derived atrophy and layer measurements |
| Pfau et al., 2019 | Prospective natural history | 36 | GA secondary to AMD | SD-OCT+ FAF+ NIR | Single visit | Natural history characterisation of photoreceptor loss in GA |
| Pfau et al., 2020 (JAMA) | Prospective longitudinal | 158 | Geographic atrophy | Spectralis SD-OCT +FAF + NIR | Median 1.1 (IQR: 0.5–1.7) | Longitudinal modelling of photoreceptor loss and GA |
| Pfau et al., 2020 (AJO) | Prospective observational | 41 | GA secondary to AMD | Spectralis SD-OCT | Single visit | AI-driven structure–function modelling in geographic atrophy |
| Pfau et al., 2021 (TVST) | Retrospective real-world | 99 | Neovascular AMD | Spectralis SD-OCT | 12 months | Prediction modelling of anti-VEGF treatment burden from baseline OCT |
| Pfau et al., 2022 | Post-hoc trial analysis | 192 | Non-neovascular GA | Spectralis SD-OCT + FAF | 18 months | Trial-based quantification of OCT-derived atrophy progression metrics |
| Prenner et al., 2025 | Prospective cross-sectional | 36 | Active nAMD + controls | Standard vs High-Res Spectralis OCT | Single visit | Comparative imaging performance of high-resolution OCT in active nAMD |
| Qu et al., 2018 | Retrospective cross-sectional | 15 | Advanced atrophic AMD (GA) | Spectralis SD-OCT | Single visit | Quantitative OCT analysis of photoreceptor loss patterns in advanced GA |
| Riedl et al., 2020 | Post-hoc RCT analysis | 185 | Treatment-naïve nAMD | Spectralis SD-OCT | 12 months | Trial-based OCT feature analysis in treatment-naïve neovascular AMD |
| Riedl et al., 2024 | Post-hoc longitudinal | 53 | Bilateral iAMD (no baseline atrophy) | Spectralis SD-OCT | 36 months | Longitudinal OCT assessment of structural progression in intermediate AMD |
| Rogala et al., 2015 | Cross-sectional | 122 | Early to intermediate AMD | Spectralis/Cirrus SD-OCT | Single visit | Topographic OCT layer evaluation across early and intermediate AMD |
| Russakoff et al., 2019 | Prospective observational | 71 | Early/intermediate AMD | Topcon SD-OCT | ~2 years (17–27 months) | Machine-learning prediction of AMD progression |
| Sadigh et al., 2015 | Cross-sectional | 31 | iAMD ± 1 advanced; controls | RTVue + SPectralis SD-OCT | Single visit | Quantitative outer retinal thickness assessment in intermediate AMD |
| Saeed et al., 2025 | Prospective observational | 40 | Early atrophic AMD (iRORA/cRORA) | SS-OCTA + Spectralis SD-OCT | Single visit | Structure–function assessment in early atrophic AMD using OCT and MP |
| Sarici et al., 2022 | Retrospective cohort | 137 | Early/intermediate AMD | SD-OCT | 5 years | Identification of OCT predictors of subfoveal atrophy |
| Sassmannshausen et al., 2018 | Prospective cross-sectional | 64 | Intermediate AMD; controls | Spectralis SD-OCT | Single visit | Mesopic/scotopic structure–function correlation in intermediate AMD |
| Sassmannshausen et al., 2022 | Prospective cross-sectional | 301 | Controls to late AMD | Spectralis SD-OCT + OCTA (PLEX Elite / Cirrus) | ~1 month | Test–retest reliability of OCT biomarkers |
| Sassmannshausen et al., 2023 | Prospective cross-sectional | 69 | iAMD (no iRORA/cRORA); controls | Spectralis SD-OCT+ FAF+NIR | Single visit | Structure–function evaluation of photoreceptor integrity in iAMD |
| Savastano et al., 2022 | Retrospective cross-sectional + longitudinal | 141 | Early/intermediate non-exudative AMD | Cirrus SD-OCT | 12 months (subset) | OCT–electrophysiology evaluation of photoreceptor dysfunction over time |
| Sayegh et al., 2017 | Prospective cohort | 36 | GA with foveal sparing | Spectralis SD-OCT + NIR | 18 months | Longitudinal structure–function analysis in fovea-sparing GA |
| Schaal et al., 2015 | Retrospective correlation | 96†  96 (OCT + histology) | Advanced AMD with ORT | Spectralis SD-OCT | Single visit | Correlation of OCT-defined ORT features with histopathology |
| Schmidt-Erfurth et al., 2025 | Post-hoc longitudinal | 897 | GA secondary to AMD | Spectralis SD-OCT | 24 months | Post-hoc OCT-based quantification of atrophy progression in trial cohorts |
| Schmitz-Valckenberg et al., 2023 | Prospective observational (post-hoc) | 24 | iAMD with iRORA/cRORA | Spectralis SD-OCT+NIR | Up to 25 months | Longitudinal evaluation of early atrophic OCT features over time |
| Schweighofer et al., 2025 | Prospective cross-sectional | 20 | Intermediate AMD | Spectralis SD-OCT | Single visit | High-resolution structure–function mapping of photoreceptor loss |
| Song et al., 2022 | Retrospective cohort | 186 | Treatment-naïve nAMD | RTVue SD-OCT | 12 months | Deep-learning analysis of OCT biomarkers predicting nAMD outcomes |
| Steinberg et al., 2016 | Cross-sectional | 40 | Early/intermediate AMD with RPD; controls | Spectralis SD-OCT | Single visit | Structure–function correlation in reticular pseudodrusen phenotypes |
| Sulzbacher et al., 2015 | Prospective longitudinal | 22 | Treatment-naïve nAMD | Spectralis SD-OCT | 12 months | Longitudinal structure–function changes during anti-VEGF treatment |
| Tekin et al., 2018 | Prospective cross-sectional | 74 | GA vs fibrotic scar; controls | Spectralis SD-OCT | Single visit | Comparative photoreceptor loss profiling in atrophic vs fibrotic AMD |
| Tepelus et al., 2017 | Prospective cross-sectional | 37 | Intermediate non-neovascular AMD | Cirrus/Nidek SD-OCT | Single visit | Structure–function evaluation of photoreceptor impairment in iAMD |
| Thiele et al., 2020 | Cross-sectional validation | 40 | iAMD; aging controls | Spectralis SD-OCT | Single visit | Methodological validation of high-resolution photoreceptor measures |
| Thiele et al., 2022 | Prospective longitudinal | 280 | Intermediate AMD | Spectralis SD-OCT | Up to 36 months | Longitudinal characterisation of photoreceptor layer change in iAMD |
| Trinh et al., 2021 | Cross-sectional topography | 168 | Intermediate AMD; controls | Spectralis SD-OCT | Single visit | ETDRS-aligned topographic mapping of retinal layer thickness in iAMD |
| Trinh et al., 2022a | Cross-sectional | 153 | Intermediate AMD ± RPD | Spectralis SD-OCT | Single visit | High-density topographic analysis of photoreceptor loss |
| Trinh et al., 2022b | Cross-sectional topography | 192 | Early + intermediate AMD; controls | Spectralis SD-OCT | Single visit | High-density OCT structural topography across early/intermediate AMD |
| van Romunde et al., 2019 | Retrospective longitudinal | 51 | Advanced exudative AMD | Spectralis SD-OCT +FAF+NIR | Up to 5 years | Longitudinal multimodal profiling of advanced exudative AMD |
| Vogl et al., 2021 | Post-hoc longitudinal | 518 | Intermediate AMD | Cirrus SD-OCT | 24 months | Spatial–temporal modelling of progression events from OCT features |
| von der Emde et al., 2019 | Cross-sectional | 30 | Intermediate AMD | Spectralis SD-OCT +FAF +NIR | Single visit | Machine-learning structure–function prediction in intermediate AMD |
| Wang et al., 2019 | Retrospective cross-sectional | 17 | GA secondary to AMD | Spectralis SD-OCT+FAF +NIR | Single visit | Quantitative OCT analysis of photoreceptor loss in geographic atrophy |
| Weber et al., 2022 | Prospective observational | 30 | Early/intermediate AMD with SDD | FLIO (blue-excited FAF lifetimes) + OCT + CFP + NIR | Up to 72 months | Longitudinal evaluation of progression in SDD phenotypes |
| Woronkowicz et al., 2020 | Retrospective observational | 71 | Treatment-resistant nAMD | Spectralis SD-OCT | 6 months | En-face ELM/EZ integrity mapping after treatment modification |
| Wu et al., 2025 | Prospective longitudinal | 280 | Intermediate AMD | Spectralis SD-OCT | Up to 36 months | Longitudinal structure–function assessment |
| Wu et al., 2025a (reader study) | Multicentre reader study | 60 | Early/intermediate AMD with iRORA | Spectralis SD-OCT | Multi-visit | Reader agreement and structure–function evaluation of early atrophic OCT |
| Yang et al., 2021 | Prospective longitudinal | 45 | Intermediate AMD | Spectralis SD-OCT | 24 months mean 15.92 ± 6.99) | Longitudinal tracking of drusen-associated photoreceptor change |
| Yordi et al., 2022 | Post-hoc RCT analysis | 81 | Treatment-naïve nAMD | Spectralis/Cirrus SD-OCT | 56 weeks | Trial-based OCT analysis of photoreceptor features in nAMD |
| Yordi et al., 2024 (J Pers Med) | Retrospective longitudinal | 116 | Early to late dry AMD | Cirrus SD-OCT | 60 months | Longitudinal photoreceptor loss profiling across dry AMD stages |
| Yordi et al., 2024 (Ophthalmol Retina) | Post-hoc RCT analysis | 652 | Treatment-naïve nAMD | Spectralis SD-OCT | 48 weeks | Post-hoc OCT analysis of baseline biomarker-defined subgroups |
| Yoshida et al., 2025 | Retrospective DL modelling | 1,661 | Bilateral GA | Spectralis SD-OCT + FAF | Multi-year | Deep-learning modelling of GA growth |
| Zhuang et al., 2024 | Prospective short-term | 63 | Active nAMD | Spectralis SD-OCT+ RTVue-XR OCTA | 3 months | Short-term OCT biomarker change during anti-VEGF loading |
| **Abbreviations:** AMD, age-related macular degeneration; CAM, Classification of Atrophy Meetings; cRORA, complete retinal pigment epithelium and outer retinal atrophy; DL, deep learning; EZ, ellipsoid zone; FAF, fundus autofluorescence; GA, geographic atrophy; HMM, high-magnification module; iAMD, intermediate age-related macular degeneration; iRORA, incomplete retinal pigment epithelium and outer retinal atrophy; MP, microperimetry; nAMD, neovascular age-related macular degeneration; OCT, optical coherence tomography; OCT-A, optical coherence tomography angiography; PED, pigment epithelial detachment; RPD, reticular pseudodrusen; SD-OCT, spectral-domain optical coherence tomography; SS-OCT, swept-source optical coherence tomography. | | | | | | |

## Supplemental Table S2. Segmentation Pipelines and Analytical Approaches Used for OCT-Derived Photoreceptor Metrics in Age-Related Macular Degeneration

| **Study** | **Segmentation type** | **Software / pipeline** | **QC / adjudication** | **Human oversight** |
| --- | --- | --- | --- | --- |
| Abraham et al., 2022 | Deep learning + graph-based | ML-enhanced multilayer segmentation with DL EZ-defect detection | Manual double-review; expert QC of layer outputs | Partial |
| Bell et al., 2024 | Manual + DL | CNN-based multilayer pipeline (fluid / low-mag / high-mag models) | Two senior readers; consensus ground truth | Yes |
| Birner et al., 2024 | DL + manual hybrid | DL for EZ/drusen/HRF; manual ONL & SDD annotation | Full expert verification of co-registration and SDD | Yes |
| Birner et al., 2025 | Deep learning | GA Monitor DL for EZL/RPEL; DL for HRF/drusen | Manual QC of OCT–MP registration | Partial |
| Bogunović et al., 2017 | Semi-automated | Iowa graph-search segmentation + RF classifiers | Manual inspection of RPE/BM for regression events | Partial |
| Borrelli et al., 2019 | Manual | ImageJ-based manual delineation | Repeat grading; reproducibility checks | Yes |
| Borrelli et al., 2020 | Semi-automated | ARI RPE-elevation + EZ-reflectivity pipeline | Manual exclusion of poor-quality slabs | Partial |
| Brandl et al., 2019 | Automated + manual | Heidelberg Eye Explorer with extensive manual correction | Line-by-line correction documented | Yes |
| Carvajal et al., 2024 | Manual | Reading-centre grading using ETDRS grid | Adjudication for discrepancies | Yes |
| Cedro et al., 2023 | Manual | Manual IR/B-scan tracing in HEYEX | Single grader; CAM-defined rules | Yes |
| Cheung et al., 2024 | Semi-automated | Auto-segmentation → manual correction → MATLAB curvature analysis | Disagreement adjudication | Yes |
| Choi et al., 2025 | Semi-auto + manual | Semi-automatic drusen segmentation + manual lesion grading (Cirrus) | Excluded low-quality scans (SS threshold); reading-centre review | Yes |
| Cicinelli et al., 2024 | Automated + manual | Auto thickness maps → manual correction; FAF co-registration; manual DLS/SDD grading | Excellent QC; adjudication used; consistent longitudinal imaging | Yes |
| Clemens et al., 2015 | Manual | Calliper-based OCT and IR grading | Dual graders; Bland–Altman analysis | Yes |
| Corbelli et al., 2017 | Semi-automated | Automated slab generation + manual correction | Inter- and intra-observer testing | Yes |
| Corvi et al., 2023 | Hybrid | Manual B-scan grading + automated en-face hyperTD slabs | Senior reader verification | Partial |
| Coulibaly et al., 2023 | Deep learning | Fully convolutional CNN for RPEL/EZL/ELML | Expert review of DL outputs | Partial |
| Ehlers et al., 2024 | Deep learning + manual | ML-enhanced multilayer segmentation | OCT–FAF cross-confirmation; expert review | Partial |
| El Ghazi et al., 2024 | Manual + automated | Manual SDD ID → SDD subtraction → automated FociPicker cone count | Best-quality image selected; manual vessel alignment | Partial |
| Erb et al., 2025 | Manual | Manual GA and EZ annotation (FAF + OCT) | Dual graders + senior adjudication | Yes |
| Etheridge et al., 2021 | Automated + manual | Proprietary Spectralis segmentation | Manual correction in >80% scans | Yes |
| Farinha et al., 2021 | Semi-automated | Automated segmentation with full manual correction | Full-volume QC | Yes |
| Fasih-Ahmad et al., 2024 | Deep learning + manual | DL graph-based segmentation with dual-grader correction | Adjudication of ambiguous bands | Yes |
| Flores et al., 2023 | Manual | Manual grading of iRORA, drusen, HRF | Inter-observer agreement assessed | Yes |
| Flynn et al., 2018 | Manual | Manual EZ grading aligned to functional loci | Repeat functional testing for QC | Yes |
| Fragiotta et al., 2022 | Semi-automated | Heidelberg segmentation + manual correction; Fiji CVI | Careful manual QC of all layers | Yes |
| Frank-Publig et al., 2025 | AI + manual | Iowa algorithm + DL U-Net; dual manual correction | Two-step expert review | Yes |
| Futterknecht et al., 2025 | Deep learning | DL lesion detection on serial OCT + OCT–MP registration (SIFT); XML import to MAIA; interpolation modelling | Strict exclusion of poor quality; alignment verified on subset; QC threshold for clinically meaningful error | Partial |
| Gallagher et al., 2022 | Semi-automated | Validated reflectivity-based multilayer algorithm | Quality thresholds; scan-matching across visits | Partial |
| Ghoshal et al., 2020 | Automated + manual | Spectralis multilayer segmentation | ICC validation of all layers | Partial |
| Goerdt et al., 2024 | Manual | Custom ImageJ “Retinal Bands” tool | Intra-reader κ analysis | Yes |
| Griffin et al., 2021 | Manual + scripted | Manual EZ contouring + automated ROI extraction | Repeat segmentation and ROI checks | Yes |
| Hanna et al., 2021 | Deep learning | Orion® AI automated segmentation | Relies on prior validation | No |
| Heckenlaible et al., 2025 | Automated + manual | Iowa algorithms + custom Python reflectivity tool | Manual review of segmentation | Partial |
| Heiferman et al., 2015 | Manual + semi-auto | RPD manual marking (ImageJ) + semi-automated SDD thresholding/masking; vessel removal | Excluded motion/RPE tracking failures; alignment validated using vessels | Yes |
| Ho et al., 2018 | Manual | Manual mapping of sensitivity points to OCT; manual grading of pathology | Limitations noted (manual alignment; coarse OCT sampling) | Yes |
| Hong et al., 2022 | Automated + manual | Manual correction of ILM/IPL/OPL; Otsu binarization; FAZ masking; RegionFinder GA segmentation; HEYEX graph-search thickness | Artifact scans excluded; segmentation errors corrected; two graders for GA/VD | Yes |
| Itoh et al., 2016 | Automated + manual | Automated EZ detection with manual review | Test–retest repeatability | Partial |
| Kalra et al., 2022 | Deep learning + adjudication | Multilayer U-Net segmentation | Dual-tier expert correction | Partial |
| Kalra et al., 2023 | Deep learning + adjudication | Auto multilayer CNN + triple-expert review | Structured expert adjudication | Partial |
| Kar et al., 2024 | Automated + manual | OCTViewer ML multilayer segmentation (RPE/BM/EZ) → line-by-line manual correction → MATLAB radiomics + RF | Central B-scan subset analysed; generalisability flagged as limitation | Yes |
| Liermann et al., 2025 | Deep learning | DL layer segmentation + raw reflectivity extraction | Protocol standardisation; no manual editing | No |
| Mahmoudi et al., 2024 | Manual | Five-grader CAM-trained qualitative grading | AC1-based agreement | Yes |
| Mai et al., 2024 | Deep learning | Fully automated CNN for EZ/RPE loss | No manual correction | No |
| Mai et al., 2025 | Deep learning | GA Monitor / RetInSight AI | No manual correction | No |
| Morelle et al., 2023 | Deep learning | CNN predicting EZ/RPE/BM heights | Comparison with manual annotations | Partial |
| Müller et al., 2021 | Manual | Four-reader manual annotation platform | Reader consensus and Dice metrics | Yes |
| Pfau et al., 2019 | Manual | Pointwise OCT–MP annotation | Two graders; adjudication | Yes |
| Pfau et al., 2020 (AJO) | Automated + manual | Automated segmentation + dual manual correction | Expert QC | Yes |
| Pfau et al., 2020 (JAMA) | Deep learning | Deeplabv3-ResNet50 multilayer segmentation | Compared vs human readers | Partial |
| Pfau et al., 2021 | Deep learning | Validated DL segmentation + feature extraction | Strong internal QC | No |
| Pfau et al., 2022 | Deep learning | DL segmentation with contour-based analysis | OCT–FAF cross-checks | No |
| Prenner et al., 2025 | Automated + manual | Iowa algorithm + OPTIMUS manual correction | Expert correction of 50 central B-scans | Yes |
| Qu et al., 2018 | Manual | 3D-OCTOR manual segmentation | Dual graders + adjudication | Yes |
| Riedl et al., 2020 | Manual + DL | Manual EZ + DL fluid segmentation | Strict QC; expert oversight | Yes |
| Riedl et al., 2024 | Deep learning + manual | Multiple CNNs + manual ONL correction | Exclusion of segmentation failures | Partial |
| Rogala et al., 2015 | Manual | Calliper-based layer measurements | Two-grader cross-check | Yes |
| Russakoff et al., 2019 | Automated + expert check | Orion 7-layer segmentation → preprocessing → AMDnet/VGG16 | Segmentation error-checked by experts; 5-fold CV | Partial |
| Sadigh et al., 2015 | Manual | Manual layer segmentation | High-density raster QC | Yes |
| Saeed et al., 2025 | Manual | Manual CAM-feature annotation | Artifact filtering; repeat checks | Yes |
| Sarici et al., 2022 | Automated + manual | OCTViewer ML multilayer segmentation → EZ–RPE / RPE–BM maps → RF classifier | Outputs reviewed by 2 experts; manual correction; qualitative grading by experts | Yes |
| Sassmannshausen et al., 2018 | Automated + manual | Heidelberg segmentation + manual correction | Full QC of all layers | Yes |
| Sassmannshausen et al., 2022 | Deep learning | DL segmentation with raw reflectivity extraction | Automated exclusion rules | No |
| Sassmannshausen et al., 2023 | Automated + manual | Automated segmentation + manual correction; HRF polygons (ImageJ); topographic registration plugin | Full review of B-scans; exclusion of poor-quality images | Yes |
| Savastano et al., 2022 | Automated + manual | Cirrus Advanced RPE Analysis → SRI; manual EZ/ONL checks; fERG integration | QC thresholds; two graders; high κ; manual fovea ID when needed | Yes |
| Sayegh et al., 2017 | Manual | OCTAVO manual B-scan segmentation + en-face projection | ART averaging; masked grading; arbitration for ambiguity | Yes |
| Schaal et al., 2015 | Manual (qualitative) | Manual review of raster volumes to classify ORT phenotypes; OCT–histology/TEM matching | Image quality variable; denser spacing used where possible | Yes |
| Schmidt-Erfurth et al., 2025 | Deep learning | DL CNNs for RPE/EZ + A-scan classification | Trial-grade acquisition; strict operator certification | No |
| Schmitz-Valckenberg et al., 2023 | Manual | FIJI manual annotation; quantitative diameters; tolerance rule; arbitration | High QC: IR+OCT registration; multi-reader consistency checks | Yes |
| Schweighofer et al., 2025 | DL + manual hybrid | DL EZ/ONL/drusen/HRF + manual SDD + pointwise coregistration | High QC; same-day repeats; randomized device order | Partial |
| Song et al., 2022 | Deep learning | CNN for EZ/ELM presence + EfficientNet FCN (fluid/lesions) + RF prediction | Excluded poor scans; patient-level split; augmentation | No |
| Steinberg et al., 2016 | Automated + manual | Automated OPL segmentation + manual corrections; manual EZ adjustment; pointwise structure–function alignment | Dense raster; repeat functional testing; manual alignment of grids | Yes |
| Sulzbacher et al., 2015 | Manual | Manual OCT feature tagging; pointwise mapping using ImageJ + auxiliary tools | High QC: averaging; manual verification of alignment | Yes |
| Tekin et al., 2018 | Automated + manual | Auto-segmentation with manual redraw when failures; ETDRS layer export | Masked graders; layer-specific corrections | Yes |
| Tepelus et al., 2017 | Automated + manual | Navis-EX automated segmentation (ONL/RPE+OS) + Cirrus Advanced RPE Analysis | Manual verification; standardized MP | Partial |
| Thiele et al., 2020 | Scripted / semi-auto | XML export → raw processing → RPE flattening → automated ROI peak detection (MATLAB) | High-res ART100; Spectralis-only | No |
| Thiele et al., 2022 | Deep learning | DL segmentation → raw reflectivity extraction → RPE flattening → EZ/ELM peak detection | Drusen ROIs excluded; strict baseline tracking alignment | No |
| Trinh et al., 2021 | Deep learning | DL EZ/ONL/RPE → voxelwise reflectivity → en-face NOIR + EZD maps | Motion/missing slice QC; automated masking; high peak recovery | No |
| Trinh et al., 2022a | Manual + scripted | Manual correction → XML extraction → MATLAB 3600-grid sampling → clustering | Manual segmentation through drusen/RPD; signal-strength QC | Yes |
| Trinh et al., 2022b | Manual + scripted | Manual segmentation → MATLAB grid extraction → clustering → displacement correction | Signal threshold QC; exclusion of vascular shadow grids | Yes |
| van Romunde et al., 2019 | Manual | Manual callipers + categorical ELM/EZ grading; multimodal integration | Adjudication when discordant; ELM only within central radius | Yes |
| Vogl et al., 2021 | Automated + exclusions | Iowa segmentation + motion correction + vessel-guided alignment + texture analysis | ~17% excluded for segmentation/registration failures | Partial |
| von der Emde et al., 2019 | Automated + manual | Auto-segmentation → manual correction → nonlinear registration → RF model | Poor segmentation frames excluded; co-registration essential | Partial |
| Wang et al., 2019 | Manual + scripted | Manual segmentation → MATLAB smoothing → FAF quartile mapping → spatial correlation | Strict vessel landmark co-registration; intensity trimming rules | Yes |
| Weber et al., 2022 | Manual (ROI) + device pipeline | FLIO acquisition + SPCImage fitting; ROIs drawn in FLIMX; OCT used for confirmation | Device-specific constraints; ROI-based QC implicit | Yes |
| Woronkowicz et al., 2020 | Automated + manual | Spectralis SD-OCT automated multilayer segmentation (ILM, ELM, EZ, BM) with manual correction; en-face EZ/ELM loss mapping in HEYEX | Poor-quality scans excluded; manual correction applied where segmentation failed; ETDRS-centred analysis | Yes |
| Wu et al., 2025 | Manual + DL | CE-marked automated AI segmentation (Discovery, Ikerian AG) for ELM, EZ, and RPE loss maps with foveal detection and sector-based analysis | CE-marked automated AI segmentation (Discovery, Ikerian AG) for ELM, EZ, and RPE loss maps with foveal detection and sector-based analysis | No |
| Wu et al., 2025a (reader study) | Manual reader annotation | Custom cross-modality annotation software; manual identification and measurement of seven atrophy-related OCT features across B-scans | Custom cross-modality annotation software; manual identification and measurement of seven atrophy-related OCT features across B-scans | Yes |
| Yang et al., 2021 | Manual | Manual Manual SD-OCT grading of drusen morphology and overlying RPE/EZ integrity using HEYEX; categorical reflectivity and disruption assessment | Two independent graders; senior adjudication for disagreement; κ statistics reported | Yes |
| Yordi et al., 2022 | Automated + manual | Proprietary ML-enabled higher-order OCT segmentation platform for retinal layers and fluid compartments; bacillary detachment classified as SRF | Proprietary ML-enabled higher-order OCT segmentation platform for retinal layers and fluid compartments; bacillary detachment classified as SRF | Yes |
| Yordi et al., 2024 (J Pers Med) | Deep learning + expert review | DL segmentation → multi-expert review → EZ–RPE maps / attenuation / intensity index | Standardised image quality; small % excluded | Partial |
| Yordi et al., 2024 (Ophthalmol Retina) | ML + expert verification | ML segmentation (Cleveland Clinic) + BD expert review → thickness maps + fluid volumes + volatility indices | Frame-by-frame verification; indeterminate BD removed | Yes |
| Yoshida et al., 2025 | Deep learning | BM flattening → DL segmentation → EZ/RPE maps → 2D/SLIVER-net/3D CNN | Incomplete segmentation excluded; downsampling for models | No |
| Zhuang et al., 2024 | Deep learning + manual | DL segmentation with expert verification | Manual QC of impairment maps | Partial |
| **Abbreviations:** AI, artificial intelligence; CAM, Classification of Atrophy Meetings; CNN, convolutional neural network; DL, deep learning; ETDRS, Early Treatment Diabetic Retinopathy Study; EZ, ellipsoid zone; FAF, fundus autofluorescence; GA, geographic atrophy; HRF, hyperreflective foci; MP, microperimetry; OCT, optical coherence tomography; QC, quality control; RPE, retinal pigment epithelium; SDD, subretinal drusenoid deposits. | | | | |

## Supplementary Table S3 — Device & Protocol Specifications

**Purpose.** This table summarises OCT acquisition platforms, segmentation or analysis pipelines, cross-device comparability, and quality-control procedures across included studies. It provides technical context for inter-study variability and informs feasibility of cross-device harmonisation in clinical trials.

| **Study** | **Primary Device / Platform** | **Acquisition Protocol** | **Segmentation / Analysis Pipeline** | **Cross-Device Comparability** | **Processing / QC Notes** |
| --- | --- | --- | --- | --- | --- |
| Abraham et al., 2022 | Heidelberg Spectralis SD-OCT | 20°×20°, ART ≥9 | ML graph-search multilayer segmentation + DL EZ-defect detection | Not assessed (single device) | Double reader review; manual correction; masked grading |
| Bell et al., 2024 | Zeiss Cirrus + Heidelberg Spectralis | Central 2-mm volume | 3-model CNN (fluid, low-mag, high-mag) + human editing | Excellent (ICC >0.95 across vendors) | Gold-standard consensus; high reader training (>200 h) |
| Birner et al., 2024 | Spectralis SD-OCT + MP-3 + MAIA | Dense macular cube | DL for EZ/drusen/HRF + manual ONL; OCT–MP co-registration | MP devices differ (offset −3.5 dB), structure–function preserved | Manual verification of all registrations |
| Birner et al., 2025 | Spectralis SD-OCT + MP-3 + MAIA | Follow-up mode | DL (GA Monitor) for EZL/RPEL + expert refinement | MP device offsets present, biomarkers consistent | All OCT–MP points manually checked |
| Bogunović et al., 2017 | Spectralis SD-OCT | Longitudinal raster | Iowa graph-search segmentation + RF HRF classifier | Single device | Manual inspection of regression events |
| Borrelli et al., 2019 | Spectralis HR-OCT | 19 B-scans, high ART | Manual EZ/OS delineation (ImageJ) | Single device | Signal ≥25 required; strict ROI QC |
| Borrelli et al., 2020 | Zeiss PLEX Elite 9000 | SS-OCT / SS-OCTA | ARI RPE-elevation + EZ reflectivity pipeline | Single device | SSI ≥7; Phansalkar thresholding |
| Brandl et al., 2019 | Spectralis SD-OCT | ETDRS-based export | Auto segmentation + extensive manual correction | Device-specific | Up to 70% manual edits required |
| Carvajal et al., 2024 | Spectralis SD-OCT | ETDRS grid | Manual CAM-aligned grading | Single device | Consensus grading; only high-quality scans |
| Cedro et al., 2023 | Spectralis SD-OCT + FAF | Annual follow-up | Manual tracing; ELM/IS-OS scoring | Single device | Multifocal lesions summed; high QC |
| Cheung et al., 2024 | Spectralis SD-OCT; Cirrus (drusen) | Central 1-mm | Auto segmentation → manual correction → MATLAB curvature | Limited (vendor-dependent segmentation) | Low-quality scans excluded |
| Choi et al., 2025 | Zeiss Cirrus HD-OCT | 512×128 cube | Semi-automatic drusen segmentation | Cirrus-specific | SS ≥7 enforced; reading-centre review |
| Cicinelli et al., 2024 | Spectralis SD-OCT + FAF | Longitudinal | Auto thickness + manual correction | Device-specific | Dual grading; adjudication |
| Clemens et al., 2015 | Spectralis SD-OCT + IR | High ART | Manual calliper-based grading | Single device | Dual-reader scoring |
| Corbelli et al., 2017 | Spectralis FAF + Cirrus OCT-A | En-face slabs | Auto slabs → manual correction | Inter-device by design | Strict averaging; manual slab definition |
| Corvi et al., 2023 | Spectralis (B-scan) + Cirrus (en-face) | Same-day imaging | Auto en-face + manual B-scan labelling | Moderate (format-dependent) | Strict signal thresholds |
| Coulibaly et al., 2023 | Spectralis SD-OCT | 6×6-mm volumes | DL segmentation of EZL/RPEL/ELML | Single device | Expert review of all outputs |
| Ehlers et al., 2024 | Spectralis SD-OCT | Trial-grade | Automated OCT-GA detection (RPE loss) | Conceptually CAM-aligned | No strict CAM size thresholds |
| El Ghazi et al., 2024 | Spectralis HMM + SD-OCT | Best-quality frames | Manual SDD ID + automated cone count | Device-specific | Vessel-based alignment |
| Erb et al., 2025 | Spectralis SD-OCT + FAF | Longitudinal | Manual GA borders; EZ annotation | Single platform | Dual graders + adjudication |
| Etheridge et al., 2021 | Spectralis SD-OCT | ETDRS grid | Proprietary segmentation + manual editing | Device-specific | >80% manual corrections |
| Farinha et al., 2021 | Spectralis SD-OCT (EDI) | Full macular volume | Automated multilayer + manual correction | Device-specific | All B-scans manually reviewed |
| Fasih-Ahmad et al., 2024 | Spectralis SD-OCT | High-density volumes | DL graph-based segmentation + manual edits | Device-specific | Ambiguous IZ/EZ deleted |
| Flores et al., 2023 | Spectralis SD-OCT | Central scan | Fully manual grading | Device-agnostic | High intergrader agreement |
| Flynn et al., 2018 | Spectralis SD-OCT + DAC | Vertical scans | Manual grading + psychophysical modelling | Single device | Multimodal QC |
| Fragiotta et al., 2022 | Spectralis SD-OCT + PLEX Elite + MAIA | Follow-up mode | Auto segmentation + manual correction | Limited | Extensive QC of all B-scans |
| Frank-Publig et al., 2025 | Spectralis vs High-Res OCT | Identical volumes | AI presegmentation + expert correction | Not numerically interchangeable | Strict device-matched protocol |
| Futterknecht et al., 2025 | PINNACLE HR-OCT + MAIA | High-res serial volumes | DL focal-lesion detection + OCT–MP XML grids | Single ecosystem | Alignment error <0.75° |
| Gallagher et al., 2022 | Spectralis SD-OCT (EDI) | 97 B-scans | Semi-automated multilayer segmentation | Device-specific | Quality ≥25; follow-up mode |
| Ghoshal et al., 2020 | Heidelberg Spectralis SD-OCT | 20°×20°, ETDRS-centred | Automated multilayer segmentation + manual correction | Device-specific | ICC validation for all layers; consistent B-scan spacing |
| Goerdt et al., 2024 | Heidelberg High-Resolution OCT (<3 µm axial) | High ART (9–100), dense raster | Manual band identification (“Retinal Bands 2024”) | HR-OCT only | Manual foveal centring; visibility dependent on ART/SNR |
| Griffin et al., 2021 | Zeiss Cirrus HD-OCT 4000 | Directional OCT protocol | Manual EZ segmentation + automated ROI extraction | Single device | Requires stable pupil decentration; IPL normalisation |
| Hanna et al., 2021 | Topcon Triton SS-OCT | Standard macular cube | Orion AI automated multilayer segmentation | Single device | No manual correction; thin layers may be mis-segmented |
| Heckenlaible et al., 2025 | Heidelberg Spectralis SD-OCT | Standardised capture settings | Iowa Reference Algorithms + manual correction | Single device | Reflectivity normalised to GCL; drusen included in RPEDC |
| Heiferman et al., 2015 | Cirrus HD-OCT + Spectralis SD-OCT | IR + OCT co-registration | Manual RPD marking; semi-automated SDD thresholding | Limited | Vessel-based alignment; poor-quality scans excluded |
| Ho et al., 2018 | Spectralis SD-OCT + iPad perimetry | Sparse B-scan protocol | Manual pathology grading + manual point mapping | Single device | No eye tracking; coarse OCT sampling |
| Hong et al., 2022 | Spectralis OCT2 OCTA | Macular cube + OCTA | Manual layer correction + Otsu binarisation | Single device | Dual graders; artifact scans excluded |
| Itoh et al., 2016 | Zeiss Cirrus + Bioptigen Envisu | 6×6 mm and 10×10 mm | Automated EZ segmentation + en-face reconstruction | Two devices used, no harmonisation | Manual verification of segmentation |
| Kalra et al., 2022 | Zeiss Cirrus + Heidelberg Spectralis | Macular cubes | Multilayer DL segmentation + dual-tier manual correction | Excellent (92–97% accuracy across vendors) | >100k B-scans; strict patient-level splits |
| Kalra et al., 2023 | Spectralis + Cirrus | Standard macular cubes | U-Net EZ At-Risk segmentation | High (Spectralis > Cirrus) | Triple-expert correction; patch-level retraining |
| Kar et al., 2024 | Single SD-OCT scanner | 128 B-scan cube | ML segmentation + radiomics (MATLAB) | Not assessed | Central 43 B-scans; authors note generalisability limits |
| Liermann et al., 2025 | Heidelberg Spectralis SD-OCT | Raw (linear) OCT volumes | DL segmentation + raw reflectivity extraction | Single device | Strict protocol; exclusion of GA/MNV/drusen >100 µm |
| Mahmoudi et al., 2024 | HR-OCT prototype vs Spectralis | Identical 97 B-scans | Expert grading (FIJI) + quantitative borders | Direct HR vs SD comparison | HR-OCT superior reproducibility |
| Mai et al., 2024 | Spectralis SD-OCT + FAF | Trial-grade 20° volumes | Fully automated CNN for EZ + RPE loss | Single device | No manual correction; validated pipeline |
| Mai et al., 2025 | Spectralis SD-OCT | Follow-up mode, ART 16 | RetInSight / GA Monitor AI | Single device | EZ loss ≤4 µm; no manual edits |
| Morelle et al., 2023 | Spectralis + Bioptigen | Standard macular cubes | CNN predicting BM/RPE/EZ heights | Moderate (tested on 2 vendors) | Height-based outputs avoid polynomial fitting |
| Müller et al., 2021 | Topcon 3D-OCT | Standard cubes | Manual CAM-based annotation | Single device | Interreader variability partly device-related |
| Pfau et al., 2019 | Spectralis SD-OCT + S-MAIA | Dense raster + MP | Manual OCT–MP registration | Single ecosystem | Gold-standard structure–function mapping |
| Pfau et al., 2020 (AJO) | Spectralis SD-OCT + S-MAIA | Follow-up mode | Auto segmentation + manual correction + RF | Single device | Multimodal registration |
| Pfau et al., 2020 (JAMA) | Spectralis SD-OCT + FAF | 20° volumes | Two-stage DL + z-score standardisation | Single device | Normative dataset device-specific |
| Pfau et al., 2021 | Spectralis SD-OCT | 19 B-scans | DL segmentation + ML feature selection | Single device | Nested CV; SHAP interpretability |
| Pfau et al., 2022 | Spectralis SD-OCT + FAF | 49 B-scans | DL segmentation + contour-line analysis | Single device | Cirrus scans excluded |
| Prenner et al., 2025 | High-Res OCT vs Spectralis | Identical 20°×20° | Iowa segmentation + manual correction | Direct device comparison | Same scan location; strict QC |
| Qu et al., 2018 | Spectralis SD-OCT | 6×6 mm cube | Manual 3D-OCTOR segmentation | Single device | Photoshop-based defect overlays |
| Riedl et al., 2020 | Spectralis SD-OCT | 49 B-scans, ART 29 | Manual EZ + DL fluid segmentation | Single device | Strict PED thresholds |
| Riedl et al., 2024 | Spectralis SD-OCT | High-density volumes | Multiple DL CNNs + manual ONL | Single device | Drusen-height thresholds enforced |
| Rogala et al., 2015 | Spectralis SD-OCT (+ Cirrus maps) | Targeted B-scans | Manual calliper measurements | Limited | Strict lesion inclusion criteria |
| Russakoff et al., 2019 | Topcon 3D OCT-1000/2000 | Variable raster | Orion segmentation + AMDnet | Device bias minimal | 5-fold CV; expert QC |
| Sadigh et al., 2015 | RTVue-100 SD-OCT | Dense raster | Manual layer segmentation | Single device | GEE for repeated measures |
| Saeed et al., 2025 | Spectralis SD-OCT + PLEX Elite | Multimodal | Manual lesion annotation | Limited | Repeated tests if FP >25% |
| Sarici et al., 2022 | SD-OCT (OCTViewer) | Macular cubes | ML-enhanced multilayer segmentation | Single platform | Dual-reader verification |
| Sassmannshausen et al., 2018 | Spectralis SD-OCT + MP-1S | High ART | Automated + manual segmentation | Single device | Test–retest scotopic enforcement |
| Sassmannshausen et al., 2022 | Spectralis SD-OCT | Multicentre standardised | DL segmentation + raw reflectivity | Single device | Central reading centre QC |
| Sassmannshausen et al., 2023 | Spectralis SD-OCT | 61 B-scans | Automated + manual HRF annotation | Single device | Full-volume review |
| Savastano et al., 2022 | Cirrus HD-OCT 5000 | Standard cubes | Automated RPE analysis + manual checks | Single device | κ >0.9 for QC |
| Sayegh et al., 2017 | Spectralis SD-OCT | Follow-up mode | Manual OCTAVO segmentation | Single device | Masked grading |
| Schaal et al., 2015 | Spectralis SD-OCT + histology | Variable | Qualitative ORT classification | Single device | Histologic validation |
| Schmidt-Erfurth et al., 2025 | Spectralis SD-OCT | Trial-grade | DL CNN for EZ + RPE | Single device | Operator certification |
| Schmitz-Valckenberg et al., 2023 | Spectralis SD-OCT | 49 B-scans | Manual FIJI annotation | Single device | 50 µm tolerance rules |
| Schweighofer et al., 2025 | MAIA + MP-3 + Spectralis | Same-day testing | DL segmentation + manual SDD | Moderate (MP offset persists) | Randomised device order |
| Song et al., 2022 | RTVue XR Avanti | Radial 18-line scans | CNN for EZ/ELM + fluid + RF | Single device | Augmentation to reduce overfitting |
| Steinberg et al., 2016 | Spectralis SD-OCT + MP-1S | 61 B-scans | Automated + manual correction | Single device | Dense raster; repeated MP |
| Sulzbacher et al., 2015 | Spectralis SD-OCT + MP-1 | 49 B-scans | Manual OCT feature tagging | Single device | 726 mapped points |
| Tekin et al., 2018 | Spectralis SD-OCT | ETDRS export | Auto segmentation + manual redraw | Single device | Masked graders |
| Tepelus et al., 2017 | Cirrus + Nidek + MP-3 | Standard cubes | Navis-EX segmentation | Multi-vendor, analysed separately | Manual verification |
| Thiele et al., 2020 | Spectralis SD-OCT (ART100) | Raw data | XML-based peak detection | Single device | Raw linear data required |
| Thiele et al., 2022 | Spectralis SD-OCT | Follow-up mode | DL segmentation + raw reflectivity | Single device | Drusen ROIs excluded |
| Trinh et al., 2021 | Spectralis SD-OCT | 49 B-scans | DL segmentation + reflectivity maps | Single device | Motion QC |
| Trinh et al., 2022 | Spectralis SD-OCT | Full volume | Manual segmentation + clustering | Single device | Signal >15 dB |
| van Romunde et al., 2019 | Spectralis SD-OCT | Multimodal | Manual calliper grading | Single device | Adjudication used |
| Vogl et al., 2021 | Zeiss Cirrus SD-OCT | Serial volumes | Iowa segmentation + texture analysis | Single device | ~17% excluded |
| von der Emde et al., 2019 | Spectralis SD-OCT + S-MAIA | Co-registered | Auto segmentation + RF | Single device | Fixation stability enforced |
| Wang et al., 2019 | Spectralis SD-OCT + FAF + NIR | Multimodal | Manual segmentation + MATLAB smoothing | Single device | Vessel landmark registration |
| Weber et al., 2022 | FLIO + Cirrus OCT | FLIO lifetime imaging | FLIMX/FLIO analysis + manual ROI | Device-specific | Pupil dilation mandatory |
| Woronkowicz et al., 2020 | Spectralis SD-OCT | En-face mapping | Auto segmentation + manual correction | Single device | Poor-quality scans excluded |
| Wu et al., 2025 (reader) | Spectralis SD-OCT + MAIA | 97 B-scans | Manual CAM-feature annotation | Single ecosystem | AC1 high across readers |
| Wu et al., 2025 (AI) | Spectralis SD-OCT | 49 B-scans | AI EZ/ONL thresholding | Single device | No manual edits |
| Yang et al., 2021 | Spectralis SD-OCT | 6×6 mm cube | Manual drusen morphology grading | Single device | Dual graders |
| Yordi et al., 2022 | Spectralis / Cirrus | Trial-derived cubes | Automated high-order segmentation + QC | Moderate (used on 2 vendors) | Frame-by-frame review |
| Yordi et al., 2024 (Cirrus) | Zeiss Cirrus SD-OCT | 6×6 mm cube | DL EZ–RPE maps | Single device | <5% exclusions |
| Yordi et al., 2024 (Spectralis) | Spectralis SD-OCT | Follow-up mode | ML segmentation + volatility metrics | Single device | Manual BD verification |
| Yoshida et al., 2025 | Spectralis SD-OCT | 49 B-scans | DL EZ/RPE prediction models | Single device | Downsampling for 3D CNN |
| Zhuang et al., 2024 | Spectralis SD-OCT + OCTA | 61 B-scans | Manual 3D lesion tracing | Platform-specific | Dual-reader adjudication |
| **Abbreviations:** AC1, Gwet’s agreement coefficient; AI, artificial intelligence; AMD, age-related macular degeneration; AO, adaptive optics; ART, automatic real-time (OCT image averaging); AUC, area under the curve; BLD, bacillary layer detachment; BM, Bruch’s membrane; CAM, Classification of Atrophy Meetings; CE, Conformité Européenne; CNN, convolutional neural network; CV, cross-validation; DA, dark adaptation; DL, deep learning; EDI, enhanced depth imaging; ELM, external limiting membrane; ETDRS, Early Treatment Diabetic Retinopathy Study; EZ, ellipsoid zone; EZL, ellipsoid zone loss; FAF, fundus autofluorescence; FCN, fully convolutional network; FLIO, fluorescence lifetime imaging ophthalmoscopy; GA, geographic atrophy; GCL, ganglion cell layer; GEE, generalized estimating equations; GLD, greatest linear dimension; HMM, high-magnification module; HR, high resolution; HRF, hyperreflective foci; HT, hypertransmission; ICC, intraclass correlation coefficient; ICGA, indocyanine green angiography; ILM, internal limiting membrane; IR, infrared; IRF, intraretinal fluid; IS, inner segment; IZ, interdigitation zone; MAIA, Macular Integrity Assessment; ML, machine learning; MP, microperimetry; MNV, macular neovascularisation; OCT, optical coherence tomography; OCTA, optical coherence tomography angiography; OPL, outer plexiform layer; ORL, outer retinal layer; ORT, outer retinal tubulation; OS, outer segment; PED, pigment epithelial detachment; PR, photoreceptor; PRL, photoreceptor layer; QC, quality control; RF, random forest; ROI, region of interest; RPEDC, retinal pigment epithelium–drusen complex; RPD, reticular pseudodrusen; RPE, retinal pigment epithelium; SD-OCT, spectral-domain optical coherence tomography; SHAP, SHapley Additive exPlanations; SHRM, subretinal hyperreflective material; SIFT, scale-invariant feature transform; SLO, scanning laser ophthalmoscopy; SRF, subretinal fluid; SS-OCT, swept-source optical coherence tomography; TEM, transmission electron microscopy; VA, visual acuity | | | | | |

## Supplementary Table S4 — CAM Adoption & Atrophy Definitions

**Purpose.** This table summarises the extent to which included studies adopted the Classification of Atrophy Meetings (CAM) framework or equivalent reference standards for defining iRORA, cRORA, and related atrophy phenotypes, highlighting areas of harmonisation and divergence across the literature.

| **Study (Year)** | **Main OCT Focus** | **Atrophy / PR Definition Framework** | **CAM Alignment** | **Standardisation Notes** |
| --- | --- | --- | --- | --- |
| Abraham et al., 2022 | EZ loss; RPE–BM/GA | Quantitative EZ–RPE thresholds; GA = RPE–BM = 0 µm | Partial | Uses fixed thickness cut-offs aligned with later CAM concepts |
| Bell et al., 2024 | EZ & sub-RPE metrics | Layer-based anatomical definitions | Partial | Device-harmonised but not lesion-based CAM staging |
| Birner et al., 2024 | EZ, ONL, HRF, SDD | Orlando 2020; IN-OCT nomenclature | No | Pixel-level standardisation supports cross-study reuse |
| Birner et al., 2025 | EZ loss; RPE loss | CAM hierarchical atrophy model | Yes | DL tools operationalise CAM-defined RPEL and cRORA |
| Bogunović et al., 2017 | Drusen; ONL; ORB | Iowa segmentation; pre-CAM GA concepts | No | Reproducible biomarkers without CAM terminology |
| Borrelli et al., 2019 | OS/EZ volumes | Anatomical outer retinal layers | No | Standard ROIs; no atrophy staging |
| Borrelli et al., 2020 | EZ reflectivity | Fixed EZ slab above RPE | Partial | Standardised slab placement approximates CAM integrity |
| Brandl et al., 2019 | Layer thickness | IN-OCT nomenclature | Yes | Layers renamed to consensus terms |
| Carvajal et al., 2024 | iRORA; cRORA | CAM Reports 3–6 | Yes | Qualitative CAM grading on ETDRS grid |
| Cedro et al., 2023 | cRORA | CAM Report 3 | Yes | OCT-defined cRORA closely matches FAF |
| Cheung et al., 2024 | EZ/RPE integrity | CAM continuity criteria | Yes | Adds curvature as objective CAM-compatible marker |
| Choi et al., 2025 | iRORA → cRORA | CAM Reports 3–5 | Yes | Strict CAM definitions for early atrophy prediction |
| Cicinelli et al., 2024 | Foveal involvement | CAM Reports 3–5 | Yes | CAM thresholds define foveal status |
| Clemens et al., 2015 | EZ integrity | IN-OCT; pre-CAM | No | Early composite PR degeneration scoring |
| Corbelli et al., 2017 | GA mapping | Pre-CAM RPE loss | No | Structural definitions consistent with later CAM |
| Corvi et al., 2023 | iRORA/cRORA | CAM Reports 3–4 | Yes | Cross-method validation vs hyperTDs |
| Coulibaly et al., 2023 | RPE + PR loss | CAM Reports 3–5 | Yes | AI segmentation enforces CAM criteria |
| Ehlers et al., 2024 | GA detection | RPE loss + HT | Partial | Simplified CAM-like RPE loss endpoint |
| El Ghazi et al., 2024 | Cone density | SDD / ORA frameworks | No | Complements CAM PR degeneration concepts |
| Erb et al., 2025 | Central integrity | CAM GA size criteria | Partial | Emphasises PR integrity over GA area |
| Etheridge et al., 2021 | Layer thickness | IN-OCT; AREDS | No | Supports CAM principles without CAM labels |
| Farinha et al., 2021 | PRL; SDD | Rotterdam / IN-OCT | No | Early PR injury consistent with CAM sequence |
| Fasih-Ahmad et al., 2024 | IZ/EZ integrity | IN-OCT; AREDS | No | Early PR–RPE interface compromise |
| Flores et al., 2023 | iRORA; EZ | Beckman + CAM | Yes | Clear CAM-aligned iRORA definitions |
| Flynn et al., 2018 | EZ; SDD | Standard EZ break definitions | No | Supports CAM-like early PR loss |
| Fragiotta et al., 2022 | ONL/ORL | IN-OCT; Beckman | No | Structural–functional topology aligns with CAM |
| Frank-Publig et al., 2025 | OR bands; SDD | IN-OCT + CAM cRORA | Yes | Device-specific metrics suitable for CAM trials |
| Futterknecht et al., 2025 | Early focal lesions | Literature-derived PR + HT definitions | No | Harmonised but not explicitly CAM-labelled |
| Gallagher et al., 2022 | Layer thinning | Hypertransmission-based GA | No | Quantifies CAM-consistent PR+RPE loss |
| Ghoshal et al., 2020 | ONL/ORL | Ferris AMD staging | No | Early atrophy precursors |
| Goerdt et al., 2024 | HR-OCT bands | Extended IN-OCT | No | Future-proof nomenclature for CAM/AI |
| Hong et al., 2022 | GA + OCTA | CAM Report 3 | Yes | Strict CAM cRORA criteria |
| Itoh et al., 2016 | EZ–RPE volume | N-OCT consensus | No | High anatomical standardisation |
| Kalra et al., 2022 | GA segmentation | CAM Report 3 | Yes | Pixel-accurate CAM classification |
| Kalra et al., 2023 | EZ At-Risk | CAM Report 4 | Yes | Distinguishes EZ loss beyond GA |
| Kar et al., 2024 | sfGA risk | Structural RPE + PR loss | No | CAM-like concepts without CAM staging |
| Liermann et al., 2025 | rEZR | MACUSTAR / IN-OCT | Partial | Excludes GA; integrity focus |
| Mahmoudi et al., 2024 | Atrophy classes | CAM Reports 3–6 | Yes | Explicit CAM-based training |
| Mai et al., 2024 | RPE vs EZ loss | CAM-aligned GA | Yes | EZ loss captures pre-CAM PR change |
| Mai et al., 2025 | EZ/RPE loss ratio | CAM-aligned GA | Yes | Ratio reflects EZ involvement beyond cRORA |
| Müller et al., 2021 | EZ loss | AREDS + CAM | Yes | Early CAM adoption |
| Pfau et al., 2019 | Junctional zone | Histology-anchored | Yes | Mechanistic basis for CAM boundaries |
| Pfau et al., 2020–2022 | PR laminae | FAF-defined GA + CAM concepts | Partial | CAM-consistent but endpoint = GA |
| Prenner et al., 2025 | PR/RPE integrity | Anatomical definitions | No | High technical but not CAM-labelled |
| Qu et al., 2018 | Junctional GA | Pre-CAM GA | No | Foundational morphology |
| Riedl et al., 2020–2024 | EZ loss | IN-OCT; CAM-aware | Partial | Structural progression before CAM atrophy |
| Russakoff et al., 2019 | DL prediction | APOSTEL layers | No | Risk prediction, not staging |
| Saeed et al., 2025 | Local atrophy | CAM Reports 4 & 6 | Yes | Manual CAM-defined lesion annotation |
| Sarici et al., 2022 | EZ–RPE and RPE–BM thickness; sfGA prediction | Structural GA defined as RPE–BM = 0 µm; EZ–RPE attenuation from prior EZ-mapping literature | No (pre-CAM terminology) | Uses consistent quantitative OCT thresholds; concepts overlap with CAM cRORA but not labelled as such |
| Sassmannshausen et al., 2018 | Layer thickness matched to function | IN•OCT lexicon; RPEDC definitions from Wu/Sadigh | No | Dense raster and z-score normalisation support CAM-like PR integrity concepts |
| Sassmannshausen et al., 2022 | rEZR reflectivity biomarker | IN•OCT + MACUSTAR protocols | No | Integrity-focused biomarker; excludes GA and atrophy by design |
| Sassmannshausen et al., 2023 | HRF as structural risk marker | IN•OCT; Beckman iAMD | Yes | HRF defined and quantified within CAM-consistent early-atrophy risk framework |
| Savastano et al., 2022 | RORA / SRI | CAM-aligned | Yes | Quantifies early CAM-like atrophy |
| Sayegh et al., 2017 | GA area; foveal sparing | Hypertransmission-based GA; ETDRS grid | Partial | Structural GA aligns with CAM but predates CAM terminology |
| Schaal et al., 2015 | Outer retinal tubulation | IN•OCT / Spaide–Curcio anatomy | No | Provides anatomical basis for CAM outer-retinal degeneration concepts |
| Schmidt-Erfurth et al., 2025 | EZ & RPE loss | CAM-aligned endpoints | Yes | FDA-acknowledged EZ loss |
| Schmitz-Valckenberg et al., 2023 | Early atrophy | CAM definitions | Yes | Quantitative CAM feature template |
| Schweighofer et al., 2025 | EZ/ONL integrity; SDD | Beckman + validated DL OCT metrics | No | Uses standardised biomarkers without CAM lesion staging |
| Song et al., 2022 | DL-based EZ/ELM integrity | Anatomical EZ/ELM definitions | No | Predictive modelling rather than staging framework |
| Steinberg et al., 2016 | Partial outer retinal thickness | IN•OCT lexicon | No | Strong structure–function coupling predating CAM |
| Sulzbacher et al., 2015 | Fluid morphology; IS/OS integrity | Standard OCT pathology definitions | No | Pre-CAM era; fixed scan alignment and expert grading |
| Tekin et al., 2018 | ONL and RPE thickness | Spectralis layer definitions | No | Quantitative PR thinning consistent with CAM sequence |
| Tepelus et al., 2017 | RPE/OS and ONL volumes | Cirrus/Nidek segmentation | No | Standardised volumetric definitions; no atrophy staging |
| Thiele et al., 2020 | Outer retinal band reflectivity | IN•OCT nomenclature | No | Integrity biomarker not linked to atrophy definitions |
| Thiele et al., 2022 | rEZR longitudinal change | IN•OCT + volumetric DL | Partial | Excludes GA; CAM-consistent integrity concept |
| Trinh et al., 2021 | Photoreceptor integrity maps | IN•OCT nomenclature | No | Reflectivity-based PR integrity without atrophy labels |
| Trinh et al., 2022 | PR thickness topography | IN•OCT; Beckman AMD | No | High-density mapping supports CAM concepts |
| Trinh et al., 2022b | Whole-retina topography | IN•OCT 2014 | No | Technical atlas rather than disease staging |
| van Romunde et al., 2019 | ELM/EZ integrity | Conventional SD-OCT band naming | No | Manual grading predates CAM |
| Wang et al., 2019 | PR/RPE thickness vs FAF | CAM Report 3 referenced | Partial | Demonstrates correspondence without strict CAM staging |
| Weber et al., 2022 | FLIO signatures of SDD | Zweifel SDD + CAM cRORA | Yes | Late AMD defined using CAM structural criteria |
| Woronkowicz et al., 2020 | En-face EZ/ELM integrity | IN•OCT definitions | No | Quantitative integrity mapping without atrophy staging |
| Wu et al., 2025 | Early atrophic lesions | CAM Reports 3–6 | Yes | Proposes OCT endpoints for early trials |
| Wu et al., 2025 (lesion study) | Early atrophic OCT lesions | CAM Reports 3–6 | Yes | Proposes structurally and functionally robust CAM-based OCT endpoints |
| Wu et al., 2025 (quantitative) | Band-loss quantification | IN•OCT + CAM | Yes | Quantitative analogues of CAM-defined PR/RPE loss |
| Yang et al., 2021 | Drusen ultrastructure; EZ/RPE | IN•OCT; CAM-aware | Partial | Risk factors aligned with CAM progression |
| Yordi et al., 2022 | Bacillary detachment; EZ attenuation | Spaide–Curcio anatomy | No | Structural phenotype, not atrophy stage |
| Yordi et al., 2024 | EZ integrity in dry AMD | IN•OCT + ML-derived EZ–RPE | Yes | EZ metrics framed as CAM-relevant early endpoint |
| Yoshida et al., 2025 | GA prediction | CAM-consistent concepts | Partial | EZ precedes RPE collapse |
| Yoshida et al., 2025 | GA progression prediction | Spaide–Curcio; DL segmentation | Partial | Confirms CAM principle that EZ loss precedes RPE collapse |
| Zhuang et al., 2024 | PR damage in nAMD | IN-OCT / Spaide | No | Structural PR loss without atrophy staging |
| **Abbreviations:** AMD, age-related macular degeneration; AREDS, Age-Related Eye Disease Study; ART, automatic real-time (OCT image averaging); BM, Bruch’s membrane; CAM, Classification of Atrophy Meetings; cRORA, complete retinal pigment epithelium and outer retinal atrophy; DL, deep learning; EDI, enhanced depth imaging; ELM, external limiting membrane; ETDRS, Early Treatment Diabetic Retinopathy Study; EZ, ellipsoid zone; EZL, ellipsoid zone loss; FAF, fundus autofluorescence; GA, geographic atrophy; HFL, Henle fibre layer; HRF, hyperreflective foci; HT, hypertransmission; iAMD, intermediate age-related macular degeneration; iRORA, incomplete retinal pigment epithelium and outer retinal atrophy; IS, inner segment; IZ, interdigitation zone; ML, machine learning; MP, microperimetry; MNV, macular neovascularisation; OCT, optical coherence tomography; OCTA, optical coherence tomography angiography; ONL, outer nuclear layer; OPL, outer plexiform layer; ORL, outer retinal layer; ORT, outer retinal tubulation; OS, outer segment; PR, photoreceptor; QC, quality control; RORA, retinal pigment epithelium and outer retinal atrophy; RPD, reticular pseudodrusen; RPE, retinal pigment epithelium; SD-OCT, spectral-domain optical coherence tomography; SDD, subretinal drusenoid deposits; SHRM, subretinal hyperreflective material; SRF, subretinal fluid; VA, visual acuity. | | | | |

## Supplementary Table S5 — Müller Cell / Gliosis Evidence (Exploratory)

**Purpose.** This table summarises exploratory OCT evidence implicating Müller-cell–associated structures—primarily the external limiting membrane (ELM), adjacent photoreceptor layers, and reflectivity-based surrogates—in retinal integrity, functional preservation, and atrophy progression in AMD. Findings are hypothesis-generating and not yet standardised for trial endpoints.

| **Study (Year)** | **Müller / ELM-Related Metric** | **Method** | **Key Observation** | **Interpretative Implication** |
| --- | --- | --- | --- | --- |
| Abraham et al., 2022 | ELM–RPE thickness (photoreceptor layer) | Multilayer segmentation (ETDRS) | Responders had thicker PR layer | ELM-anchored PR integrity supports functional gain |
| Bell et al., 2024 | Implicit ELM anchoring (EZ–RPE) | Multilayer AI segmentation | Stable EZ–RPE metrics across devices | ELM is a reliable anatomical reference, not a biomarker here |
| Birner et al., 2025 | EZ thickness (ELM–IDZ interval) | DL segmentation | EZT strongly correlated with function | ELM-anchored EZ thickness reflects PR viability |
| Bogunović et al., 2017 | ONL / ORB thickness (ELM boundary) | Graph-theoretic segmentation | Thinner ONL/ORB → drusen regression risk | PR–Müller-adjacent layers involved in drusen dynamics |
| Borrelli et al., 2019 | OS/EZ relative to ELM | Manual tracing | Abnormal OS response after bleaching | Outer retinal stress, not direct Müller metric |
| Borrelli et al., 2020 | EZ reflectivity (ELM-anchored) | En-face reflectivity mapping | Reduced EZ reflectivity beyond drusen | Diffuse PR dysfunction; Müller role indirect |
| Cedro et al., 2023 | ELM disruption score | Manual grading (0–3) | ELM disruption correlated with BCVA decline | ELM integrity reflects structural reserve |
| Cheung et al., 2024 | EZ curvature / continuity | Manual + quantitative curvature | EZ distortion predicts progression | Subtle PR–ELM interface distortion measurable |
| Choi et al., 2025 | ELM loss as part of iRORA | CAM-based grading | PR degeneration present at iRORA onset | ELM loss integral to earliest atrophy |
| Cicinelli et al., 2024 | ONL thinning; BM–ELM dynamics | Central OCT metrics | ONL thinning predicts foveal involvement | PR nuclear loss dominates functional risk |
| Clemens et al., 2015 | EZ integrity (qualitative) | Manual grading | EZ loss → reduced sensitivity | PR/ELM complex central to early dysfunction |
| Corvi et al., 2023 | ELM/EZ disruption in CAM | Manual B-scan grading | En-face methods miss ELM loss | ELM assessment requires dense B-scans |
| Coulibaly et al., 2023 | EZL / RPEL ratio | AI segmentation | PR loss precedes RPE loss | Upstream PR–ELM degeneration |
| Ehlers et al., 2024 | PR–RPE complex loss | Automated OCT-GA | PR loss detectable before FAF GA | OCT captures early PR/Müller-linked damage |
| El Ghazi et al., 2024 | EZ integrity vs cone density | SD-OCT + HMM | Lower cone density where EZ disrupted | PR degeneration reflects metabolic stress |
| Erb et al., 2025 | EZ integrity index (EZII) | Manual continuity mapping | Central vs parafoveal EZ relates to VA/LLVA | Spatial PR loss patterns |
| Etheridge et al., 2021 | PR / ONL thickness | Layer maps | PR thinning predicts BCVA | Photoreceptor soma loss dominates |
| Farinha et al., 2021 | PRL & ONL thinning | Layer thickness maps | SDD → accelerated outer retinal loss | Müller–PR unit vulnerable in SDD |
| Fasih-Ahmad et al., 2024 | IZ integrity | IZ boundary discernibility | IZ best reflects rod dysfunction | Retinoid transfer zone affected early |
| Flores et al., 2023 | EZ integrity; HRF | Manual grading | EZ loss predicts progression | PR-level degeneration prognostic |
| Flynn et al., 2018 | EZ integrity | Locus-based grading | EZ loss linked to DA delay | PR dysfunction precedes atrophy |
| Frank-Publig et al., 2025 | ELM band clarity (HR vs SD OCT) | Device comparison | HR-OCT improves ELM visibility | Resolution limits confound ELM loss |
| Futterknecht et al., 2025 | EZ/IZ loss + HT lesions | DL lesion detection + MP | Severe focal sensitivity loss | Müller–PR complex failure at focal lesions |
| Gallagher et al., 2022 | OSL thickness (ELM→EZ/IZ) | Volumetric segmentation | OSL thinning tightly coupled to RPE loss | PR outer segments degenerate with RPE |
| Goerdt et al., 2024 | ELM band visibility (HR-OCT) | High-resolution OCT band scoring | ELM visibility declines before EZ loss | Müller–ELM disruption may precede overt PR degeneration |
| Heckenlaible et al., 2025 | ELM stability vs EZ decline | Volumetric DL segmentation | ELM preserved while EZ reflectivity declines | Müller scaffold remains intact despite PR metabolic failure |
| Heiferman et al., 2015 | SDD proximity to ELM | Manual SDD mapping | SDD associated with altered ELM contour | Müller–RPE metabolic interface stressed in SDD |
| Ho et al., 2018 | EZ/ELM integrity vs sensitivity | Manual grading + tablet perimetry | Loss of EZ/ELM—not fluid—drives sensitivity loss | Structural PR–Müller integrity dominates function |
| Hong et al., 2022 | ELM disruption at GA margins | Manual OCT + OCTA | ELM disruption precedes choriocapillaris loss | Müller–PR compromise occurs early in GA expansion |
| Itoh et al., 2016 | ELM-referenced EZ–RPE volume | Volumetric EZ slab analysis | EZ attenuation detected before RPE loss | Early PR/Müller-linked degeneration measurable |
| Kalra et al., 2022 | ELM loss within GA (DL) | Pixel-level CAM-aligned segmentation | ELM loss tightly co-localises with cRORA | Müller-cell disruption integral to complete atrophy |
| Kalra et al., 2023 | EZ At-Risk zone (ELM intact) | DL segmentation | Large zones of EZ loss with preserved ELM | Potential therapeutic window before Müller collapse |
| Kar et al., 2024 | PR texture changes adjacent to ELM | Radiomics (ELM-anchored ROIs) | Texture changes precede thickness loss | Subtle PR stress before Müller/ELM breakdown |
| Liermann et al., 2025 | rEZR (EZ/ELM reflectivity) | Peak reflectivity analysis | ELM stable; EZ declines | rEZR isolates PR mitochondrial dysfunction |
| Mahmoudi et al., 2024 | ELM disruption width | Manual annotation | HR-OCT improves ELM reliability | ELM disruption measurable with adequate resolution |
| Mai et al., 2024 | ELM integrity within EZ loss | DL EZ/RPE loss maps | ELM preserved in early EZ loss regions | Müller cells may maintain structural scaffold early |
| Mai et al., 2025 | EZ/RPE loss ratio (ELM reference) | Volumetric AI segmentation | EZ loss exceeds RPE loss over time | Upstream PR–Müller injury |
| Müller et al., 2021 | EZ integrity (qualitative) | Manual grading | Variable EZ loss without consistent ELM grading | Highlights need for explicit Müller/ELM metrics |
| Pfau et al., 2019 | Residual HFL+ONL at GA edge | OCT labelling | Residual nuclei support function | Müller/PR remnants retain partial function |
| Pfau et al., 2020–2022 | ELM descent; ONL thinning | DL segmentation + z-scores | ELM marks hard atrophy edge | ELM loss reflects terminal PR/Müller failure |
| Prenner et al., 2025 | ELM loss area | IOWA segmentation (SD vs HR) | Less apparent loss on HR-OCT | Apparent ELM loss partly resolution-driven |
| Riedl et al., 2020 | ELM continuity | Manual grading | ELM disruption linked to poor recovery | Müller integrity influences reversibility |
| Riedl et al., 2024 | ONL thinning relative to ELM | Volumetric segmentation | ONL loss accelerates after ELM disruption | Müller–PR coupling failure marks transition |
| Rogala et al., 2015 | PR thinning over drusen (ELM-referenced) | Manual callipers | PR thinning proportional to drusen height | Müller-mediated metabolic stress hypothesis |
| Saeed et al., 2025 | ELM disruption | Manual OCT + MP | ELM predicts deep sensitivity loss | ELM integrity more specific than EZ |
| Sarici et al., 2022 | ELM-adjacent PR thickness | Automated segmentation | Thinner PR layer predicts sfGA | ELM-anchored PR loss prognostic |
| Sassmannshausen et al., 2018 | ELM stability vs function | Layer thickness z-scores | ELM more stable than EZ/ONL | Müller scaffold relatively preserved |
| Sassmannshausen et al., 2022 | rEZR stability (ELM reference) | Reflectivity ratio | ELM reflectivity stable across stages | Supports use of ELM as internal normaliser |
| Sassmannshausen et al., 2023 | HRF proximity to ELM | Manual HRF annotation | HRF cluster near ELM predicts progression | Müller activation / gliosis marker |
| Schaal et al., 2015 | ORT ELM hyperreflectivity | OCT–histology correlation | ELM persists without PRs | Müller cells form reflective scaffold |
| Schmitz-Valckenberg et al., 2023 | ELM disruption extent | Manual annotation | ELM disruption progresses over time | Early Müller–PR interface breakdown |
| Schweighofer et al., 2025 | ELM integrity vs MP repeatability | OCT + MP repeatability | Reduced ELM integrity worsens test–retest | Müller health affects functional reliability |
| Song et al., 2022 | DL-derived EZ/ELM integrity | CNN prediction model | EZ/ELM features improve outcome prediction | Müller–PR integrity adds prognostic value |
| Steinberg et al., 2016 | Partial outer retinal thickness | OPL–RPE slab | PR thickness correlates with rod sensitivity | Müller–PR metabolic unit |
| Sulzbacher et al., 2015 | Fluid vs EZ/ELM status | Manual OCT grading | EZ/ELM integrity outweighs fluid type | Müller–PR axis dominates function |
| Tekin et al., 2018 | ELM-adjacent ONL thickness | Volumetric OCT | Thinner ONL associated with biomarker changes | PR soma loss; Müller involvement indirect |
| Tepelus et al., 2017 | RPE+OS volume (ELM referenced) | Volumetric slabs | OS volume strongest predictor | Müller–PR outer segment coupling |
| Thiele et al., 2020 | ELM reflectivity stability | Raw OCT reflectivity | ELM reflectivity stable across AMD stages | Müller band provides internal reference |
| Thiele et al., 2022 | rEZR longitudinal change | ELM-referenced reflectivity | rEZR declines while ELM stable | Progressive PR mitochondrial dysfunction |
| Trinh et al., 2021 | ELM-adjacent reflectivity maps | DL reflectivity mapping | PR stress precedes thickness loss | Müller–PR metabolic disturbance |
| Trinh et al., 2022 | PR topography relative to ELM | High-density OCT mapping | Peripheral PR loss with intact ELM | Spatial dissociation of PR loss and Müller scaffold |
| van Romunde et al., 2019 | ELM integrity | Manual OCT grading | Preserved ELM predicts better VA | Müller integrity = functional reserve |
| Wang et al., 2019 | ELM/PR thickness vs FAF | Manual + automated analysis | Structural loss exceeds FAF-defined GA | OCT captures Müller–PR injury earlier |
| Weber et al., 2022 | SDD overlying ELM | Multimodal OCT–FLIO | Altered lifetimes where ELM distorted | Müller metabolic involvement in SDD |
| Woronkowicz et al., 2020 | ELM loss area | En-face mapping | ELM correlates with BCVA better than EZ | ELM may reflect functional reserve |
| Wu et al., 2025 | ELM disruption ≥500 µm | Manual CAM feature annotation | Predicts repeatable deep defects | ELM loss defines non-responding retina |
| Wu et al., 2025 | ELM loss ≥250–500 µm | CAM-based OCT annotation | Defines non-functional retina | Müller disruption as endpoint-level event |
| Yang et al., 2021 | EZ/ELM over drusen | Manual grading | Early ELM distortion over large drusen | Mechanical/metabolic stress at Müller junction |
| Yordi et al., 2022 | BLD and ELM displacement | Volumetric OCT | ELM displaced but recoverable | Reversible Müller stretch injury |
| Yordi et al., 2024 | Persistent EZ loss with intact ELM | Longitudinal AI maps | EZ recovers incompletely despite intact ELM | Müller scaffold ≠ PR recovery guarantee |
| Yoshida et al., 2025 | ELM role in GA prediction models | DL feature importance | ELM contributes modestly to prediction | EZ dominates, ELM secondary |
| Zhuang et al., 2024 | ELM damage adjacent to SHRM | 3D OCT tracing | SHRM predicts focal ELM disruption | Inflammatory/toxic injury to Müller–PR junction |
| **Abbreviations:** AMD, age-related macular degeneration; ART, automatic real-time (OCT image averaging); BCVA, best-corrected visual acuity; BLD, bacillary layer detachment; BM, Bruch’s membrane; CAM, Classification of Atrophy Meetings; cRORA, complete retinal pigment epithelium and outer retinal atrophy; DA, dark adaptation; DL, deep learning; ELM, external limiting membrane; ETDRS, Early Treatment Diabetic Retinopathy Study; EZ, ellipsoid zone; EZL, ellipsoid zone loss; FAF, fundus autofluorescence; GA, geographic atrophy; HFL, Henle fibre layer; HRF, hyperreflective foci; HT, hypertransmission; IRF, intraretinal fluid; IS, inner segment; IZ, interdigitation zone; LLVA, low-luminance visual acuity; ML, machine learning; MP, microperimetry; OCT, optical coherence tomography; OCTA, optical coherence tomography angiography; ONL, outer nuclear layer; OPL, outer plexiform layer; ORT, outer retinal tubulation; OS, outer segment; PR, photoreceptor; QC, quality control; RPE, retinal pigment epithelium; SD-OCT, spectral-domain optical coherence tomography; SDD, subretinal drusenoid deposits; SHRM, subretinal hyperreflective material; SRF, subretinal fluid; VA, visual acuity. | | | | |
